# Nitrogen-doped polycyclic aromatic hydrocarbons by a one-pot Suzuki coupling/intramolecular S_N_Ar reaction†

**DOI:** 10.1039/d2sc05409d

**Published:** 2022-11-29

**Authors:** Xiaoqi Tian, Kazutaka Shoyama, Frank Würthner

**Affiliations:** a Universität Würzburg, Institut für Organische Chemie Am Hubland Würzburg 97074 Germany wuerthner@uni-wuerzburg.de; b Universität Würzburg, Center for Nanosystems Chemistry (CNC) Theodor-Boveri-Weg Würzburg 97074 Germany

## Abstract

We report a new method for the synthesis of nitrogen-doped (N-doped) polycyclic aromatic hydrocarbons (PAHs) by a Suzuki coupling/intramolecular S_N_Ar cascade reaction. A one- or two-fold [3 + 3] naphtho-annulation of halogenated aniline was conducted under Suzuki–Miyaura cross-coupling conditions to yield a series of fully fused N-doped PAHs. In contrast to reported methods to synthesize pyridinic or pyrrolic nitrogen-doped PAHs, our method enables preparation of PAHs doped with graphitic nitrogen, for which few reports are known in the literature. The crystal structure as well as absorption, fluorescence and electrochemical properties of these N-doped PAHs were investigated, which demonstrated the capability of N-doping to adjust optical and electronic properties and alter the LUMO energy level.

## Abstract 2

We report a new method for the synthesis of N-doped PAHs by a Suzuki coupling/intramolecular S_N_Ar cascade reaction. N-doping was demonstrated to be an effective strategy to adjust optical and electrochemical properties, and the LUMO energy levels.

## Graphical abstract

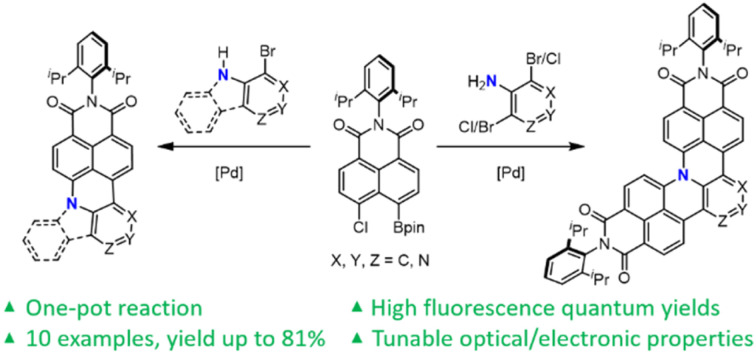

## Introduction

**Fig. 1:**
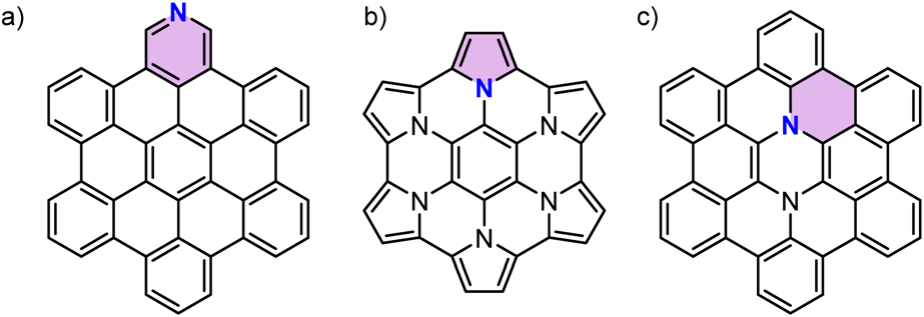
Chemical structures of the reported N-doped PAHs with different types of doped nitrogen atoms. For each structure an aromatic ring containing a specific type of doped nitrogen atom is highlighted by color. Substituents are omitted for clarity. (a) Pyridinic N-doped PAH.^5^ (b) Pyrrolic N-doped PAH.^6^ (c) Graphitic N-doped PAH.^7^

Nitrogen-doping (N-doping) of polycyclic aromatic hydrocarbons (PAHs) is an efficient strategy to tailor the optical and electrochemical properties of parent PAHs.^1,2^ Similar to N-doped graphene^3,4^ the type of nitrogen species in PAHs could also be distinguished as pyridinic nitrogen,^5^ pyrrolic nitrogen,^6^ and graphitic nitrogen^7^ depending on the site of the nitrogen atom (Fig. 1). The type of doped nitrogen atom greatly influences the properties of N-doped PAHs. While pyridinic nitrogen affords more electron-deficient PAHs, the other two give rise to more electron-rich PAHs due to an increase in the number of π-electrons. Furthermore, different from pyrrolic nitrogen that involves a five-membered ring, graphitic nitrogen can be seen as a direct replacement of a carbon atom in an aromatic six-membered ring by nitrogen, thereby adding one electron to the respective π-system. Interest in synthesizing PAHs doped with a specific type of nitrogen arises from the debate over the properties and reactivity of N-doped graphene with different types of doped nitrogen.^8–13^ Synthesis of N-doped PAHs with well-defined sizes and specific concentrations and positions of nitrogen atoms provides an opportunity to accurately investigate the effects of N-doping on graphitic carbon. However, N-doped PAHs reported in the literature have principally been limited to those containing pyridinic and pyrrolic nitrogen atoms which could be easily obtained by replacing benzene rings with pyridine or pyrrole.^1^ Few examples of graphitic N-doped PAHs have been reported, which required multiple-step syntheses.^7,14–19^ This scarcity of PAHs with graphitic nitrogen might be due to the lack of a synthetic strategy that can form six-membered rings around a nitrogen atom. One of the few examples of such methods has recently been reported by Lindner and co-workers,^18^ where a one-pot Buchwald–Hartwig cross-coupling/direct arylation reaction^20–22^ was used to synthesize a luminescent N-doped PAH for OLEDs. However, there have been no attempts at the development of a versatile synthetic method to access a series of graphitic N-doped PAHs.

**Scheme 1:**
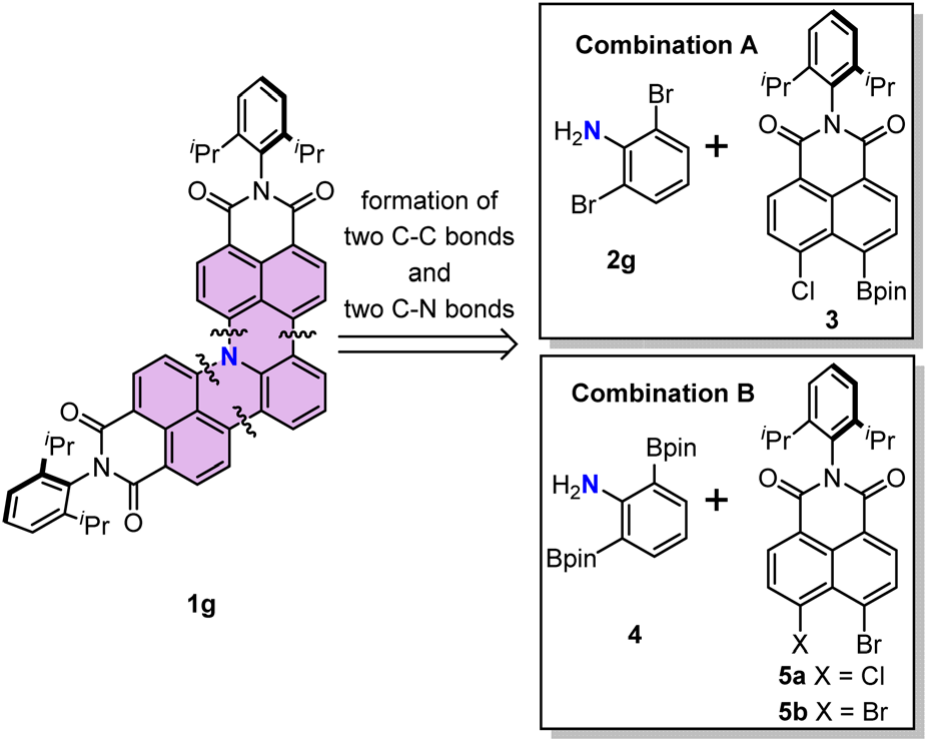
Retrosynthetic analysis and design of building blocks. The structure of 15*b*-azatribenzo[*de*,*hi*,*op*]tetracene is highlighted in color.

Here we introduce a synthetic method for graphitic N-doped PAHs and investigate their properties. Thus, we designed 15*b*-azatribenzo[*de*,*hi*,*op*]tetracene (Scheme 1) as naphthalene-bridged aniline and developed a cascade reaction for [3 + 3] naphtho-annulation of halogenated aniline. In order to stabilize the electron-rich N-doped π-scaffold imide substituents are installed to lower the oxidation potential. The optimized reaction conditions yielded a series of N-doped PAHs containing graphitic nitrogen. Furthermore, pyridinic or pyrrolic nitrogen could also be installed using substrates already containing such nitrogen. The synthesized N-doped PAHs exhibited high stability under ambient conditions and high fluorescence quantum yields. Thus, we anticipate that the new class of N-doped imide-functionalized PAHs provides an important extension of the structural and functional space of so-far explored imide-functionalized PAHs and nanographenes.^23,24^

## Results and discussion

We first describe the retrosynthetic analysis for the target molecule 1g. This molecule possesses one aniline and two naphthalimide moieties. Thus, 1g might be obtained from naphthalimide and an aniline derivative through formation of two C–C bonds and two C–N bonds (Scheme 1). We chose the Suzuki–Miyaura cross-coupling reaction for the formation of the two C–C bonds, because it has been developed as the most widely used method to construct a C–C bond.^25,26^ The Buchwald–Hartwig amination reaction or nucleophilic aromatic substitution reaction is a potential method to construct the desired C–N bond.^27,28^ Therefore, we decided to use readily available 2,6-dibromoaniline (2g) as the aniline coupling partner and *N*-(2,6-diisopropylphenyl)-5-chloro-naphthalene-1,8-dicarboximide-4-boronic acid (pinacol)ester (3) as a boron reagent to optimize the reaction conditions (Scheme 1, combination A). An alternative combination of aniline-2,6-diboronic acid bis(pinacol)ester (4) and *N*-(2,6-diisopropylphenyl)-4,5-dihalo-naphthalene-1,8-dicarboximide (5) (Scheme 1 combination B)^29,30^ was also examined.

**Scheme 2:**
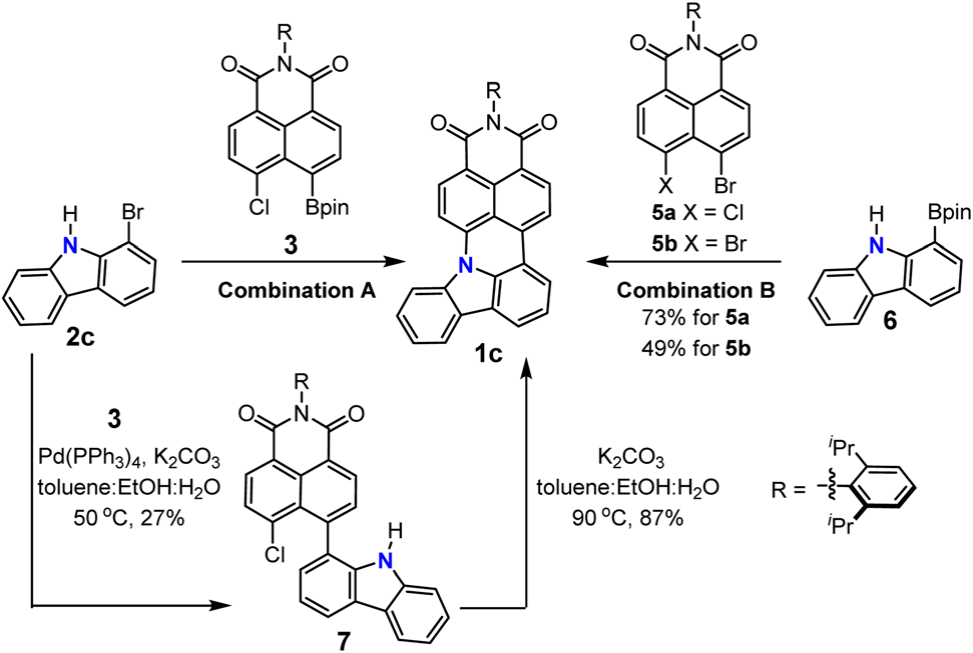
Reaction pathway towards 1c.

After preparation of building block 3 (details of synthesis described in the ESI†), the mono-annulation reaction with 2c was used as a model reaction to optimize the reaction conditions (Table 1 and Scheme 2). Considering the requirement of the formation of the C–C bond and C–N bond, we evaluated a dual catalyst system for both the Suzuki–Miyaura coupling and Buchwald–Hartwig amination reactions, *i.e.* Pd(PPh_3_)_4_ for C–C bond formation and Pd_2_(dba)_3_/(*t*-Bu)_3_P for amination (entries 1–3). Among the three tested conditions, only Pd_2_(dba)_3_/Pd(OAc)_2_ with P(*t*-Bu)_3_/P(Cy)_3_ as the ligand and NaO^*t*^Bu as the base in toluene could afford the desired product in 50% yield (entry 2). Because the yield was only moderate for the dual ligand system, we hypothesized that the combination of Suzuki coupling and intramolecular S_N_Ar cascade reactions might give higher yields. To our delight, reactions using four commonly used catalysts for Suzuki coupling and a base for both Suzuki coupling and S_N_Ar reactions all could afford the desired product (entries 4–7), with the reaction using Pd(PPh_3_)_4_/K_2_CO_3_ showing the best performance with 77% yield (entry 7). Subsequently, the influence of base was explored as well (entries 7–9). Among the used bases, K_2_CO_3_ is the most suitable one for this cascade reaction; weaker or stronger bases such as Na_2_CO_3_ or Cs_2_CO_3_ both lead to lower yields. Furthermore, the necessity of water and ethanol as co-solvents in this reaction was proved by the control reaction using toluene as the sole solvent, which afforded the desired product only in 11% yield (entry 10). We further applied the optimized conditions to combination B (Scheme 2), which uses boronic ester substituted aniline as the coupling partner. A comparable result was obtained when 6 and 5a were applied as the substrates (entry 11); however, the yield was decreased to 49% when 5b was used as the coupling partner (entry 12).

**Table 1:** Optimization of the reaction conditions for the Suzuki coupling/intramolecular S_N_Ar cascade reaction^a^

	Catalyst	Ligand	Base	Solvent	Yield^b^
1	Pd_2_(dba)_3_/Pd(PPh_3_)_4_	P(*t*-Bu)_3_	NaO*t*Bu	Toluene	—
2	Pd_2_(dba)_3_/Pd(OAc)_2_	P(*t*-Bu)_3_/P(Cy)_3_	NaO*t*Bu	Toluene	50
3	Xphos Pd G2/Pd(PPh_3_)_4_	—	NaO*t*Bu	Dioxane	—
4	Pd(dppf)Cl_2_	—	K_2_CO_3_	Toluene : EtOH : H_2_O	75
5	Pd(OAc)_2_	Sphos	K_3_PO_4_	*n*-Butanol : H_2_O	25
6	Xphos Pd G2	Xphos	K_2_CO3	Toluene : EtOH : H_2_O	61
7	Pd(PPh_3_)_4_	—	K_2_CO_3_	Toluene : EtOH : H_2_O	77
8	Pd(PPh_3_)_4_	—	Na_2_CO_3_	Toluene : EtOH : H_2_O	35
9	Pd(PPh_3_)_4_	—	Cs_2_CO_3_	Toluene : EtOH : H_2_O	60
10	Pd(PPh_3_)_4_	—	K_2_CO_3_	Toluene	11
11^c^	Pd(PPh_3_)_4_	—	K_2_CO_3_	Toluene : EtOH : H_2_O	73
12^d^	Pd(PPh_3_)_4_	—	K_2_CO_3_	Toluene : EtOH : H_2_O	49

a 2c (40 μmol, 1.0 equiv.), 3 (2.0 equiv.), catalyst (0.10 equiv.), ligand (0.20 equiv.), base (3.0 equiv.), solvent (toluene : EtOH : H_2_O 5 : 2 : 1, *n*-butanol : H_2_O 2 : 1), 90 °C, 16 h.

b Isolated yield.

c 6 and 5a as substrates.

d 6 and 5b as substrates. SPhos: 2-dicyclohexylphosphino-2′,6′-dimethoxybiphenyl, XPhos: 2-dicyclohexylphosphin-2′,4′,6′-triisopropylbiphenyl, PCy_3_: tricyclohexylphosphine, P(^*t*^Bu)_3_: tri(*tert*-butyl)phosphine, dppf: 1,1′-bis(diphenylphosphino)ferrocene, dba: dibenzylideneacetone.

The proposed reaction pathway of this cascade reaction is illustrated in Scheme 2. Because the applied reaction conditions are those for a typical Suzuki coupling reaction and not suitable for Buchwald–Hartwig amination, the first step of this cascade should be a common Suzuki coupling reaction to form the C–C bond. This could also be proved by a control experiment using the conditions in Table 1 entry 7 but at lower temperature and a shorter reaction time (50 °C for 4 hours), where the intermediate 7 could be isolated (the relatively low yield of 7 is due to the high reactivity of 7, which could convert to the final product 1c fast under the reaction conditions) and its structure was unambiguously confirmed by ^1^H and ^13^C NMR spectroscopy as well as high-resolution mass spectrometry. Thus, the second step of this cascade reaction should be a nucleophilic aromatic substitution reaction which benefits from the electron-withdrawing character of dicarboximide to increase the ability of the chloride substituent as the leaving group and also stabilize the Meisenheimer complex.^31^ This hypothesis was substantiated by the almost quantitative transformation of 7 to 1c under basic conditions in a toluene, ethanol, and water mixture without any palladium catalyst. These results support our reaction pathway for the N-doped PAHs *via* a Suzuki coupling/intramolecular S_N_Ar cascade reaction.

**Scheme 3:**
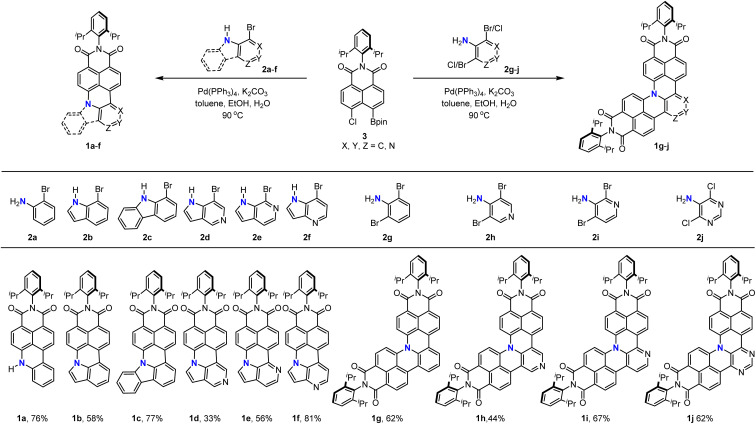
Substrate scope of the palladium-catalyzed cascade reaction for the synthesis of N-doped PAHs.

With the optimized reaction conditions in hand, we turned our attention to the substrate scope of this cascade annulation (Scheme 3). 2-Bromoaniline 2a, 7-bromoindole 2b, and 1-bromo-9*H*-carbazole 2c all could be coupled with 3 to afford the corresponding N-doped PAHs 1a–c in 58–77% yield. Moreover, azaindole with nitrogen at a different position could also be applied to this reaction, furnishing the products 1d–f in moderate to high yields. Finally, we used these reaction conditions for the synthesis of double-annulated products. N-doped PAHs 1g–i could be isolated in 44–67% yields when 2,6-dibromoaniline 2g, 3,5-dibromo-4-pyridinamine 2h, and 2,4-dibromo-3-pyridinamine 2i were applied. Encouraged by the above excellent results, we then extended this catalytic system to 5-amino-4,6-dichloropyrimidine 2j, which is a difficult substrate for the Suzuki coupling reaction due to coordination of the substrate onto the palladium catalyst and the lower reactivity of chlorine. The desired product 1j could be isolated in 62% yield, demonstrating the wide substrate scope of this synthetic method.

**Fig. 2:**
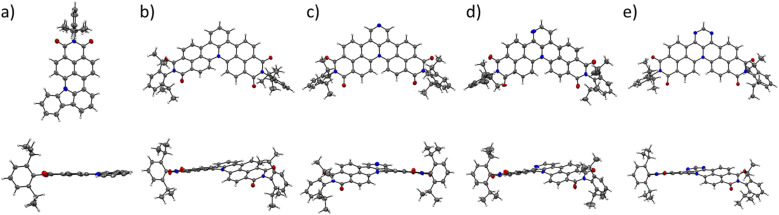
Solid-state molecular structures of (a) 1c, (b) 1g, (c) 1h, (d) 1i, and (e) 1j determined by single-crystal X-ray diffraction analysis. Thermal ellipsoids are set at 50% probability.

**Fig. 3:**
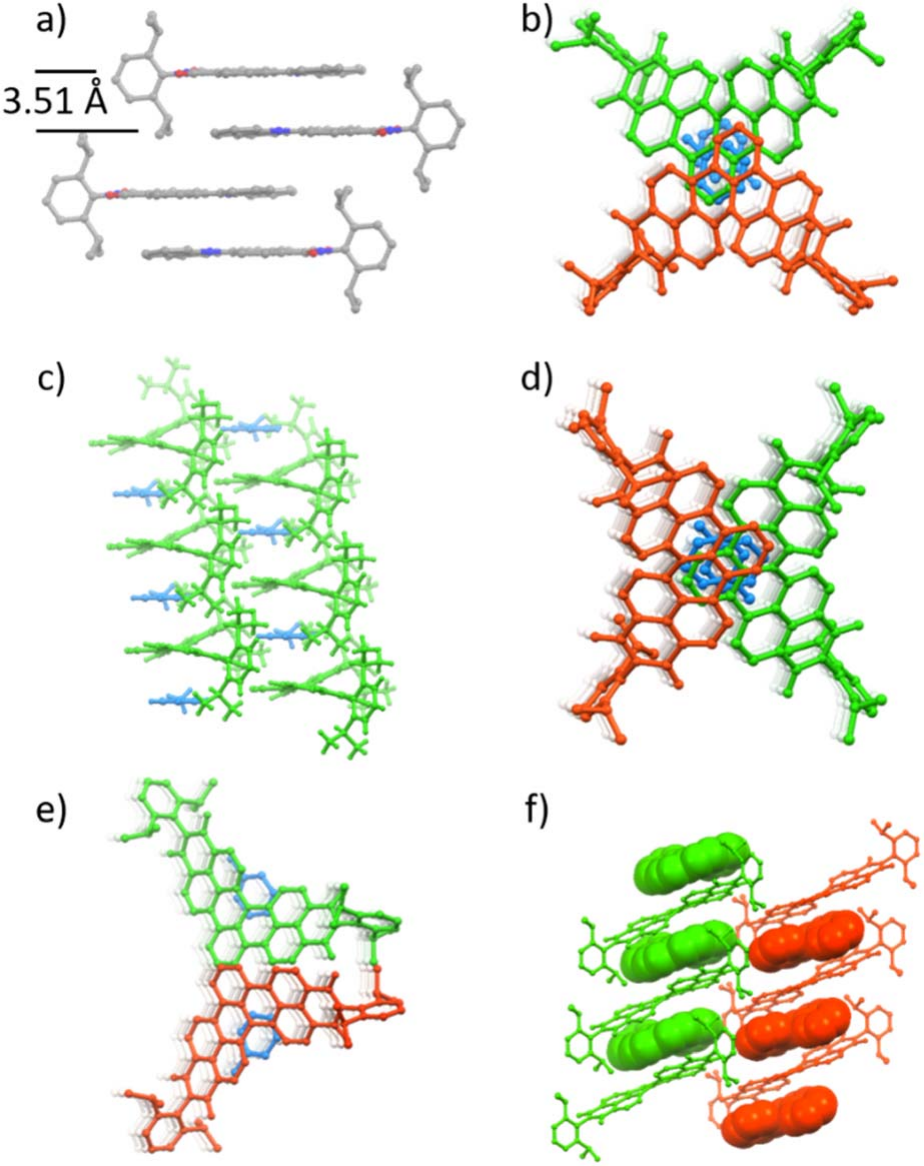
Packing arrangement of (a) 1c, (b) 1g, (c) 1h, (d) 1i, (e) 1j, and (f) 1g/[4]helicene. Blue: toluene, green: *M* configuration, and red: *P* configuration. Hydrogen omitted for clarity. For (f) guest [4]helicene is depicted by a space filling model.

All N-doped PAHs were fully characterized by ^1^H and ^13^C NMR spectroscopy as well as high-resolution mass spectrometry (ESI†). Furthermore, single crystals of 1c and 1g–j suitable for X-ray analysis were grown by slow diffusion of hexane or methanol into their dichloromethane or toluene solutions and thus their molecular structures could be unambiguously confirmed. N-doped PAHs 1c and 1j crystallize in a monoclinic crystal system with *P*2_1_/*n* and *P*2_1_/*c* space groups, respectively. Other bisimides 1g, 1h, and 1i crystallize in a triclinic crystal system with a *P*1̄ space group for 1g and 1i and *P*1̄ space group for 1h. Mono-annulated compound 1c is almost planar with a small torsion angle (5°, Fig. 2a). The solid-state packing arrangement of 1c (Fig. 3a) is characterized by tightly stacked head-to-tail antiparallel dimers with average π–π distances of approximately 3.51 Å. Twofold-annulated compounds 1g–j are nonplanar and possess a [4]helicene backbone with average torsion angles of 26.5°, 24.4°, 25.9°, and 22.6° in the inner helix, respectively (Fig. 2b–e). The Gibbs activation energies of enantiomerization (Δ*G*^‡^(*T*)) of 1g–i were calculated at the B3LYP/6-31G(d) level of theory to be 15.4, 15.1, and 15.2 kcal mol^−1^, respectively, while the Δ*G*^‡^(*T*) of 1j was significantly larger with 19.7 kcal mol^−1^. All these calculated activation energies were much larger than that of unsubstituted [4]helicene (4.5 kcal mol^−1^ calculated at the B3LYP/6-31G(d) level of theory).

Unlike the planar structure of mono-annulated compounds, which could form dimer packing motifs through π–π interactions (Fig. 3a), double-annulated compounds 1g–j did not show direct π–π interaction between molecules; instead one or two molecules of toluene stacked in between. Both 1g and 1i have almost the same packing structure, stacks of centrosymmetric head-to-head dimers comprising *M*- and *P*-configuration isomers (Fig. 3b and d). The chiral crystal structure of 1h was solved as racemic twins, where 1h and toluene solvate formed alternating columnar stacks with average π–π distances of 3.67 Å (Fig. 3c). There is only one molecule of 1h in the asymmetric unit of this chiral crystal with the *P*1 space group, which aligns this [4]azahelicene unit-containing 1h without any other symmetry operation than translational symmetry in the solid-state, giving this molecule the potential to be applied to nonlinear optical technologies (Fig. 3c).^32^ Bisimide 1j formed alternating columnar stacks with toluene, where two adjacent stacks are separated by a mirror plane (Fig. 3e). The packing structure of double-annulated compounds inspired us to explore their potential for the growth of co-crystals of these compounds with suitable guest molecules. Indeed, we succeeded in growing co-crystals of 1g with [4]helicene (Fig. 3f). Bisimide 1g formed a columnar stack with [4]helicene with the same configuration in the crystal structure, indicating the ability of this molecule to host guest molecules of the same chirality.

**Fig. 4:**
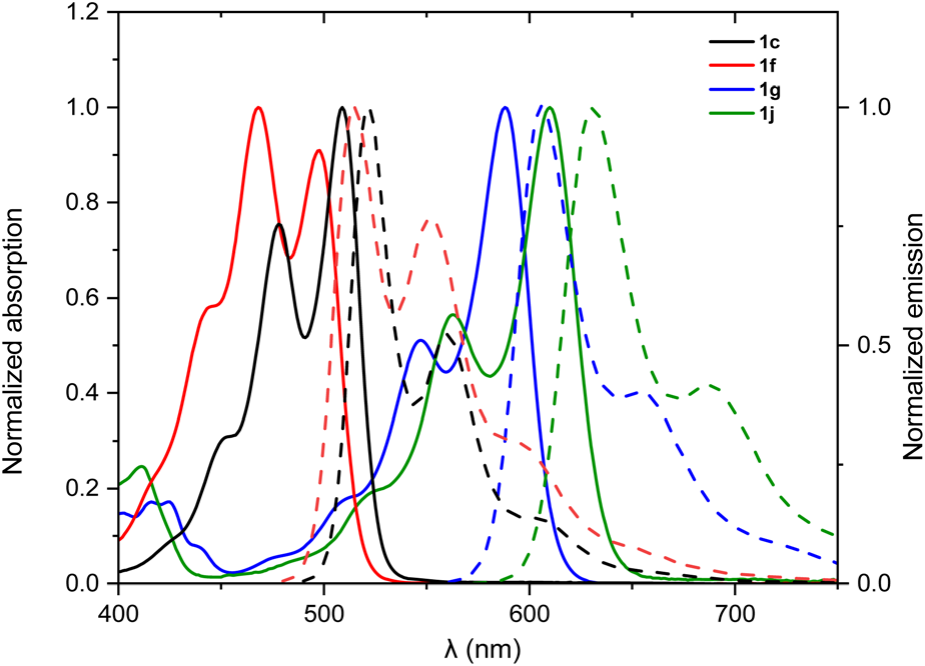
Absorption (solid line) and emission (dotted line) spectra of 1c, 1f, 1g, and 1j (chloroform solution, *c* ∼ 4 × 10^−6^ M for UV-vis, OD ∼ 0.05 for fluorescence at 293 K).

The optical properties of the new N-doped PAHs 1a–j were investigated by UV/vis absorption spectroscopy and fluorescence emission spectroscopy in chloroform solutions at room temperature (Fig. 4, S1–S3† and Table 2). All N-doped PAHs show absorption bands with clearly resolved vibronic structures and small Stokes shifts (490–720 cm^−1^), indicating relatively small changes in their geometry in ground and excited states. Mono-annulated compounds 1a–f exhibit similar absorption and emission spectral features with perylene monoimides (PMIs).^33^ Interestingly, mono-annulated compounds are also highly fluorescent with fluorescence quantum yields close to unity, thereby even surpassing the parent PMI (Table 2). 1a–c show very similar absorption (longest wavelength absorption maxima are located at 505 nm) and emission spectra (longest wavelength emission maxima are located at 522 nm) with different extinction coefficients due to different sizes of the aromatic π-system (Fig. S1†). The lowest energy absorption maxima of 1d–f are at 486, 504, and 497 nm, respectively, which are slightly hypsochromically shifted to the lowest energy absorption maxima of 1a–c (505–509 nm). This is in good accordance with their electrochemical energy gaps (*vide infra*). Fluorescence lifetime (FL) for mono-annulated compounds ranges from 5.93 to 7.73 ns. Double-annulated compounds 1g–j show a well-resolved S_0_–S_1_ transition at *λ*_abs_ = 588, 560, 598, and 610 nm, respectively, with a shoulder located in the higher energy region and a mirror-image fluorescence with *λ*_em_ of 607, 578, 617, and 631 nm, respectively. Pyrimidine-containing 1j exhibits the largest values for *λ*_abs_ and *λ*_em_ among these ten N-doped PAHs. The fluorescence quantum yields of these double-annulated compounds are 23–84%, which are smaller compared to those of the mono-annulated products. We attribute these low quantum yields to the enhanced intersystem crossing caused by their non-planar [4]helicene backbone.^34^ The FLs of 1i (6.49 ns) and 1j (6.47 ns) are similar to those of mono-annulated compounds; however the FLs of 1g (3.14 ns) and 1h (1.74 ns) are much smaller compared to those of mono-annulated compounds. The experimental absorption spectra of the newly synthesized N-doped PAHs are in good agreement with their TDDFT calculated spectra (Fig. S8–S10†). Overall, the absorption maxima of these ten N-doped PAHs could be changed from 486 nm (1d) to 610 nm (1j), demonstrating the capability of N-doping to adjust optical properties of PAHs.

**Table 2:** Optical properties of the N-doped PAHs

	*λ*_abs_ a (nm)	*λ*_em_ b (nm)	*ε* [M^−1^ cm^−1^]	*ϕ*_fl_ c [%]	*E*_HOMO_ d [eV]	*E*_LUMO_ d [eV]
1a	505	524	21 400	94	—	—
1b	508	521	35 700	99	−5.97	−3.40
1c	509	522	44 300	99	−5.95	−3.35
1d	486	500	31 200	99	−6.09	−3.47
1e	504	519	33 300	99	−6.12	−3.56
1f	497	515	25 000	99	−6.20	−3.58
1g	588	607	70 000	38	−5.93	−3.66
1h	560	578	59 500	23	−6.09	−3.74
1i	598	617	65 900	84	−6.01	−3.81
1j	610	631	63 400	64	−6.09	−3.96
PMI^e^	507	545	31 000	85	−6.10	−3.69

a Measured in chloroform (*c* ∼ 4.0 × 10^−6^ M).

b Measured in chloroform (OD ∼ 0.05).

c Determined using the absolute quantum yield method.

d Calculated using the equations *E*_LUMO_ = −[*E*(*M*/*M*^−^) + 5.15 eV] and *E*_HOMO_ = −[*E*(*M*/*M*^+^) + 5.15 eV], assuming that the energy level of Fc^+^/Fc with respect to the vacuum level is −5.15 eV.

e Data from ref. 32 and 34.

The redox properties of newly synthesized N-doped PAHs 1b–j were investigated by cyclic and square wave voltammetry (1a was insoluble for this investigation). The measurements were performed in dichloromethane at room temperature using tetrabutylammonium hexafluorophosphate as electrolyte and the ferrocenium/ferrocene (Fc^+^/Fc) redox couple as the internal standard. The voltammograms are displayed in Fig. S7† and the electronic properties are summarized in Table S2.† All measured mono-annulated compounds 1b–f showed one reduction and one oxidation process and double-annulated compounds 1g–j showed two reduction and two oxidation processes. The HOMO and LUMO energy levels of these N-doped PAHs were calculated based on their first oxidation/reduction waves and are summarized in Table 2. While the first reduction processes of all nine measured molecules were reversible, the first oxidation process was reversible only for 1c and 1g–1j. Other mono-annulated compounds 1b and 1d–f showed pseudo-reversible or irreversible first oxidation processes presumably due to the unstable α-double bond of the (aza)indole subunit. The HOMO energy levels of these N-doped PAHs are very similar to those of PMI;^35^ however, mono-annulated products show much higher LUMO energy levels compared with PMI, reflecting the electron-donating character of the doped graphitic/pyrrolic nitrogen. Azaindole annulated products 1d–f (−3.47 to −3.58 eV) exhibited lower LUMO energy levels compared with indole and carbazole annulated products 1b (−3.40 eV) and 1c (−3.35 eV) due to the electron-withdrawing character of azaindole. The LUMO energy levels of 1g–j were −3.66, −3.74, −3.81, and −3.96 eV, respectively, which are much lower compared with those of mono-annulated products due to the second dicarboximide. The order of the LUMO energy level was 1j < 1i < 1g, which could be explained by the fact that pyridine and pyrimidine are much more electron deficient compared to benzene. The LUMO energy levels of these N-doped PAHs clearly demonstrated the effect of different types of doped nitrogen atoms on the LUMO energy level.

## Conclusions

In summary, a new Suzuki coupling/intramolecular S_N_Ar cascade reaction was developed for rationally designed building blocks. Ten N-doped PAHs were efficiently synthesized by our approach in moderate to good yields, including four double-annulated compounds. This cascade reaction could be applied to form PAHs containing different types of nitrogen, such as graphitic, pyrrolic and pyridinic, demonstrating the versatility of this cascade reaction. The crystal structures of 1c and 1g–j were determined by single crystal X-ray diffraction analysis and their structures were unambiguously confirmed, where the torsion angles of these N-doped PAHs range from *ca.* 5° (mono-annulated products) to *ca.* 25° (double-annulated products). The optical properties and electrochemical properties of these N-doped PAHs were systematically investigated. Mono-annulated products 1a–f exhibited high fluorescence quantum yields (94–99%) and small Stokes shifts (490–720 cm^−1^). The absorption maxima of annulated products varied over 125 nm (0.52 eV), from 486 nm to 610 nm and the LUMO energy levels were between −3.35 eV and −3.96 eV, demonstrating the usefulness of N-doping to adjust optical and electronic properties. Furthermore, this cascade reaction might also be applicable for the synthesis of larger sized N-doped PAHs and would expand the scope of available N-doped PAHs.

## Experimental methods

### General procedure

2a–f (40.0 μmol, 1.00 equiv.), compound 3 (80.0 μmol, 2.00 equiv.), Pd(PPh_3_)_4_ (4.00 μmol, 0.100 equiv.), and potassium carbonate (0.120 mmol, 3.00 equiv.) were charged in a 15 mL Schlenk tube under an argon atmosphere. Then 0.8 mL toluene–EtOH–H_2_O (5 : 2 : 1) was added into the flask *via* a syringe. The reaction mixture was heated at 90 °C under intense stirring and an argon atmosphere overnight. After cooling to room temperature, the reaction mixture was diluted with dichloromethane, washed with water and brine, dried over anhydride sodium sulfate, and filtered. After removal of solvent, the crude product was purified by column chromatography on silica-gel and precipitation from dichloromethane/cyclohexane to afford the product. For the synthesis of double-annulated products 2g–j (40.0 μmol, 1.00 equiv.), compound 3 (0.160 mmol, 4.00 equiv.), Pd(PPh_3_)_4_ (8.00 μmol, 0.200 equiv.), and potassium carbonate (0.240 mmol, 6.00 equiv.) were applied.

## Data availability

All experimental procedures and spectroscopic data can be found in the ESI.†

## Author contributions

Xiaoqi Tian: investigation, methodology, writing. Kazutaka Shoyama: single crystal X-ray analysis writing, supervision. Frank Würthner: conceptualization, writing, supervision, funding acquisition.

## Conflicts of interest

There are no conflicts of interest to declare.

## Supplementary Material

SC-014-D2SC05409D-s001

SC-014-D2SC05409D-s002
