# Supplementary material for: Nitrogen-doped polycyclic aromatic hydrocarbons by a one-pot Suzuki coupling/intramolecular SNAr reaction
Source: Chem Sci. 2022 Nov 29;14(2):284–90. doi: 10.1039/d2sc05409d (PMC9811559; doi:10.1039/d2sc05409d)
Supplement: SC-014-D2SC05409D-s001 [file SC-014-D2SC05409D-s001.pdf]

Electronic Supplementary Material (ESI) for Chemical Science.
This journal is © The Royal Society of Chemistry 2022

Supporting Information

Nitrogen-Doped Polycyclic Aromatic Hydrocarbons by a One-Pot Suzuki Coupling/Intramolecular S_NAr Reaction

Xiaoqi Tian[a], Kazutaka Shoyama[a], and Frank Würthner[a,b,*]

[a] Institut für Organische Chemie, Universität Würzburg, Am Hubland, 97074 Würzburg, Germany

[b] Center for Nanosystems Chemistry (CNC), Universität Würzburg, Theodor-Boveri-Weg, 97074 Würzburg, Germany

Contents

1. Materials and methods 3
2. Synthesis 5
3. Optical properties 10
4. Electrochemical properties 15
5. Theoretical calculations 17
6. Single crystal X-ray analysis 21
7. NMR spectra of new compounds 27
8. Mass spectra 39
9. Cartesian Coordinates 43
10. References 75

1. Materials and methods

General remarks: Unless otherwise stated, all reactions were carried out using reagents obtained from commercial sources and used without further purification. All the reactions were carried out in dry Schlenk tubes under protection of nitrogen using standard Schlenk technique. Column chromatography was performed on silica-gel (particle size 0.040−0.063 mm).

NMR spectroscopy were recorded on Bruker Avance III HD 400 MHz and 600 MHz spectrometers. Chemical shift data for protons are reported in parts per million (ppm, δ scale) downfield from tetramethylsilane and referenced internally to the residual proton in the solvent ($CDCl_3$: δ 7.26 ppm, DMSO-d_6: δ 2.50). Chemical shift data for carbons are reported in parts per million (ppm, δ scale) downfield from tetramethylsilane, and referenced internally to the carbon resonance in the solvent ($CDCl_3$: δ 77.00, DMSO-d_6: δ 39.52). NMR data analysis are presented as following, s: singlet, d: doublet, t: triplet, m: multiplet, br: broad signal, coupling constant in Hertz (Hz), and integration.

High resolution mass spectra were measured on a Bruker Daltonics micrOTOF-QIII focus instrument for high-resolution ESI or a Bruker Daltonics ultrafleXtreme mass spectrometer using DCTB as a matrix for high-resolution MALDI. As a note, we like to mention that the often observed peak at 685.4 is attributable to Irganox, an additive of plastics that contaminates the samples upon dissolution from plastic vials for mass spectrometry.

Cyclic and square wave voltammetry were measured with a standard commercial electrochemical analyzer (EC epsilon; BAS Instruments, UK) with a three-electrode single-compartment cell. Tetrabutylammonium hexafluorophosphate (n-Bu_4NPF_6) was applied as supporting electrolyte, ferrocene (Fc) as an internal standard for the calibration of potentials, Ag/AgCl as reference electrode, Pt disc and Pt wire as working and auxiliary electrodes, respectively. Cyclic voltammetry (CV) and square wave voltammetry (SWV) were performed at a scan rate of 100 mV/s at room temperature. The redox potentials were referenced against the ferrocenium/ferrocene (Fc^+/Fc) redox couple.

UV/Vis absorption spectra were recorded on JASCO V-670 and V-770 spectrometers.

Fluorescence spectra and lifetime measurements were measured with an Edinburgh Instruments FLS980 spectrometer. Lifetimes were measured using EPL picosecond pulsed diode laser (479.7 nm and 505.8 nm) as a light source. Fluorescence quantum yields were determined relative to common fluorescence standards for optically dilute solutions ($A < 0.05$).[S1]

Theoretical calculations were performed by Gaussian 16 program[S2] at B3LYP/6-31G(d) level. Optimized ground state geometries were examined by frequency analysis to possess no negative frequency.

Single-crystal X-ray crystallography: Diffraction data for **1c**, **1g**, **1h**, and **1i**, were collected on a Bruker D8 Quest Kappa diffractometer with a PhotonII detector and multi-layered mirror monochromated CuKα radiation at 100 K. Diffraction data for **1g**·[4]helicene and **1j** were collected at the P11 beamline at DESY by a single 360° ϕ sweep scan at 100 K, and indexed, integrated and scaled using the XDS program package.[S3] The structures were

solved using direct methods using SHELXT,[S4] expanded with Fourier techniques and refined with SHELXL.[S5] All non-hydrogen atoms were refined anisotropically. Hydrogen atoms were included in the structure factor calculation at geometrically idealized positions. Disordered alkyl substituents were modelled with restraints using standard SHELXL commands ISOR and SIMU. Disordered solvent molecules found in solvent accessible voids were modelled with constraints using standard SHELXL commands DFIX, FLAT, SAME, SADI, SIMU, ISOR, and RIGU.

Verification of the crystal structure for **1g**·[4]helicene using PLATON[S6] software package generated two level-A alerts. One of them is due to low completeness of measured data, which was caused by a single 360° data collection at the P11 beamline in the synchrotron DESY. The other alert is due to high wR_2, which we attribute to relatively large thermal factor of this co-crystal and disorder in the solvent accessible voids. Due to pseudo-symmetry of this crystal structure around the C_2 axis of [4]helicene, PLATON[S6] also generated a B-level alert to indicate a higher symmetry of the crystal structure with an additional *n*-glide plane. With careful inspection of the structure we concluded that there is slight deviation of geometry around this [4]helicene moiety from the assumed *n*-glide plane and the lower symmetry $P\bar{1}$ is the true space group.

The crystal structure of **1i** was solved as 5-component twin. Domains and twin operations were determined by TwinRotMat implemented in PLATON[S6] and HKLF5 data were generated by the same program. A level-A alert indicating a high wR_2 index was generated by PLATON,[S6] which we attribute to large error of diffraction intensity due to twinning involving five domains.

2. Synthesis

Starting material **5b**[S7] and **6**[S8] were synthesized following the reported procedure.

Synthesis of *N*-(2,6-diisopropylphenyl)-4-bromo-5-chloronaphthalene-1,8-dicarboximide (5a)

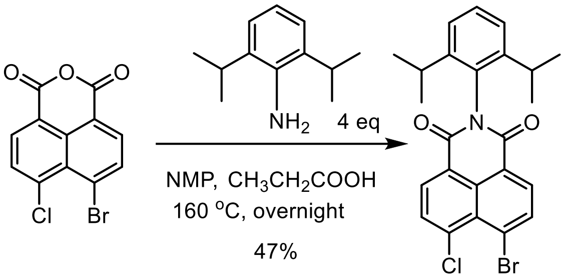

8 **5a**

This compound was synthesized following a reported procedure[S9] with minor modification.
4-Bromo-5-chloronaphtalene-1,8-dicarboxylic anhydride (**8**)[S9] (500 mg, 1.61 mmol, 1.00 equiv.), 2,6-diisopropylaniline (1.10 g, 6.44 mmol, 4.00 equiv.), and acetic acid (3 mL), *N*-methyl-2-pyrrolidone (3 mL) were charged in a flask. The reaction mixture was heated at 160 °C under intense stirring and argon atmosphere overnight. After cooling and addition of water the resulting precipitate was collected and further purified by silica-gel column chromatography (hexane:dichloromethane = 1:1) to give **5** (356 mg, 47%) as a yellow solid. ^{1}H NMR spectrum was in accordance with that reported in literature.[S9]

Synthesis of *N*-(2,6-diisopropylphenyl)-5-chloronaphthalene-1,8-dicarboximide-4-boronic acid (pinacol)-ester (3)

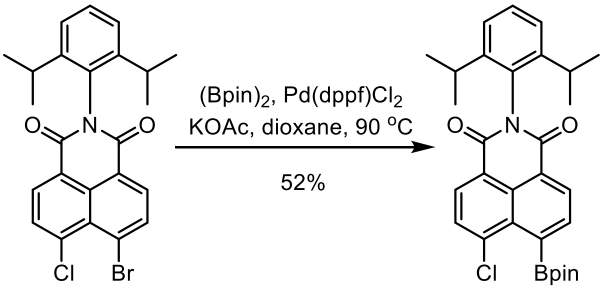

5a **3**

N-(2,6-Diisopropylphenyl)-4-bromo-5-chloronaphthalene dicarboximide (**5a**) (500 mg, 1.06 mmol, 1.00 equiv.), bis(pinacolato)diboron (404 mg, 1.60 mmol, 1.50 equiv.), [1,1'-bis(diphenylphosphino)ferrocene]dichloro-palladium(II) (36.6 mg, 50.0 μmol, 5.00 mol %), and potassium acetate (312 mg, 3.18 mmol, 3.00 equiv.) were charged in a 50 mL Schlenk tube under an argon atmosphere. Then anhydrous dioxane (20 mL) was added into the flask via a syringe. The reaction mixture was heated at 90 °C under intense stirring and argon atmosphere overnight. After cooled to room temperature, the reaction mixture was diluted with dichloromethane, washed with water and brine, dried over anhydride sodium sulfate, and filtered. After removal of solvent, the crude product was purified by column chromatography on silica-gel (*n*-hexane/dichloromethane 2:1) to afford the product as a yellow solid (285 mg, 52%).
m.p. 241–242 °C.

^{1}H NMR (400 MHz, $CDCl_3$), δ (ppm): 8.67 (d, *J* = 7.2 Hz, 1H), 8.57 (d, *J* = 7.9 Hz, 1H), 7.93 (d, *J* = 7.2 Hz, 1H), 7.88 (d, *J* = 7.9 Hz, 1H), 7.47 (t, *J* = 7.8 Hz, 1H), 7.32 (d, *J* = 7.8 Hz, 2H), 2.74–2.64 (m, 2H), 1.48 (s, 12H), 1.14 (d, *J* = 6.9 Hz, 12H).
^{13}C NMR (101 MHz, $CDCl_3$): δ = 163.8, 163.5, 145.6, 140.0, 133.1, 131.7, 131.6, 131.2, 130.5, 129.8, 129.6, 127.8, 124.0, 123.8, 121.9, 84.9, 29.1, 24.8, 23.9 ppm.
HRMS (ESI, pos. mode, $CHCl_3$/MeCN): m/z: 540.21044 $[M]^+$ (calcd. for $C_{30}H_{33}BClNNaO_4^+$: 540.20834).

Synthesis of 1a–f

General procedure: 2-bromoaniline or bromoindole (40.0 μmol, 1.00 equiv.), compound **3** (80.0 μmol, 2.00 equiv.), $Pd(PPh_3)_4$ (4.00 μmol, 0.100 equiv.), and potassium carbonate (0.120 mmol, 3.00 equiv.) were charged in a 15 mL Schlenk tube under an argon atmosphere. Then 0.8 mL toluene–EtOH–H_2O (5 : 2 : 1) was added into the flask via a syringe. The reaction mixture was heated at 90 °C under intense stirring and argon atmosphere for overnight. After cooled to room temperature, the reaction mixture was diluted with dichloromethane, washed with water and brine, dried over anhydride sodium sulfate, and filtered. After removal of solvent, the crude product was purified by column chromatography on silica-gel and precipitation from dichloromethane/cyclohexane to afford the product.

***N*-(2,6-Diisopropylphenyl)-7*H*-benzo[*kl*]acridine-3,4-dicarboximide (1a)**.
Cyclohexane/ethyl acetate (5:1), orange solid, 76% yield. m.p. > 350 °C
^{1}H NMR (400 MHz, DMSO-d_6) δ 12.12 (s, 1H), 8.46 (d, *J* = 8.1 Hz, 1H), 8.44–8.39 (m, 1H), 8.33 (d, *J* = 8.5 Hz, 1H), 8.15 (d, *J* = 8.3 Hz, 1H), 7.65–7.61 (m, 1H), 7.46–7.38 (m, 2H), 7.34 (ddd, *J* = 8.2, 7.0, 1.2 Hz, 1H), 7.30 (d, *J* = 7.7 Hz, 2H), 7.06 (d, *J* = 8.5 Hz, 1H), 2.60–2.54 (m, 2H), 1.05 (d, *J* = 6.9 Hz, 12H).
^{13}C NMR (101 MHz, DMSO-d_6): δ = 163.5, 163.1, 145.4, 144.5, 138.1, 137.6, 134.2, 133.1, 132.4, 132.0, 131.7, 128.6, 124.5, 123.5 (two signals), 120.1, 118.9, 117.3, 116.1, 113.6, 108.1, 106.1, 28.5, 23.6 ppm.
HRMS (ESI, pos. mode, $CHCl_3$/MeCN): m/z: 469.18599 $[M]^+$ (calcd. for $C_{30}H_{26}N_2NaO_2^+$: 469.18865).

***N*-(2,6-Diisopropylphenyl)-11b-aza-11b*H*-benzo[*hi*]aceanthrylene-8,9-dicarboximide (1b)**.
Cyclohexane/ethyl acetate (5:1), orange solid, 58% yield. m.p. > 350 °C
^{1}H NMR (400 MHz, $CDCl_3$) δ 8.68 (d, *J* = 7.9 Hz, 1H), 8.64 (d, *J* = 8.2 Hz, 1H), 8.16 (d, *J* = 8.0 Hz, 1H), 8.02 (d, *J* = 7.6 Hz, 1H), 7.86 (d, *J* = 3.5 Hz, 1H), 7.75 (dd, *J* = 7.8, 0.7 Hz, 1H), 7.52 (d, *J* = 8.3 Hz, 1H), 7.49–7.42 (m, 2H), 7.33 (d, *J* = 7.8 Hz, 2H), 6.92 (d, *J* = 3.4 Hz, 1H), 2.77–2.70 (m, 2H), 1.17 (d, *J* = 6.8 Hz, 12H).
^{13}C NMR (101 MHz, $CDCl_3$): δ = 164.0, 163.7, 145.6, 139.5, 135.9, 133.9, 133.1, 132.4, 132.0, 131.3, 129.3, 128.5, 124.7, 124.6, 124.0, 122.9, 122.1, 119.6, 119.2, 118.8, 117.0, 115.5, 109.8, 106.6, 29.1, 24.0 ppm
HRMS (ESI, pos. mode, $CHCl_3$/MeCN): m/z: 493.18974 $[M]^+$ (calcd. for $C_{32}H_{26}N_2NaO_2^+$: 493.18865).

***N*-(2,6-Diisopropylphenyl)-9b-aza-9b*H*-dibenzo[*a,hi*]aceanthrylene-6,7-dicarboximide (1c)**.
Cyclohexane/ethyl acetate (10:1), orange solid, 77% yield. m.p. > 350 °C
^{1}H NMR (400 MHz, $CDCl_3$), δ (ppm): 8.73 (d, *J* = 2.0 Hz, 1H), 8.71 (d, *J* = 1.5 Hz, 1H), 8.28 (d, *J* = 8.5 Hz, 1H), 8.17–8.13 (m, 3H), 8.08 (d, *J* = 8.3 Hz, 2H), 7.69–7.65 (m, 1H), 7.56–7.46 (m, 3H), 7.35 (d, *J* = 7.8 Hz, 2H), 2.81–2.74 (m, 2H). 1.21 (d, *J* = 2.0 Hz, 6H), 1.19 (d, *J* = 2.0 Hz, 6H)

^{13}C NMR (101 MHz, $CDCl_3$): δ = 164.1, 163.7, 145.6, 141.3, 138.6, 135.7, 135.5, 134.1, 133.0, 132.0, 131.3, 129.3, 128.0, 126.8, 124.6, 124.4, 124.0, 123.9, 123.3, 122.3, 121.6, 121.0, 119.6, 119.0, 116.2, 114.7, 114.5, 107.8, 29.1, 24.0 ppm

HRMS (ESI, pos. mode, $CHCl_3$/MeCN): m/z: 543.20185 $[M]^+$ (calcd. for $C_{36}H_{28}N_2NaO_2^+$: 543.20430).

N-(2,6-Diisopropylphenyl)-4,11b-diaza-11bH-benzo[hi]aceanthrylene-8,9-dicarboximide (1d)

Cyclohexane/ethyl acetate (2:1), orange solid, 33% yield. m.p. > 350 °C

^{1}H NMR (400 MHz, $CDCl_3$) δ 9.17 (s, 1H), 8.98 (s, 1H), 8.71 (d, *J* = 7.8 Hz, 1H), 8.66 (d, *J* = 8.2 Hz, 1H), 8.29 (d, *J* = 7.9 Hz, 1H), 7.88 (d, *J* = 3.5 Hz, 1H), 7.57 (d, *J* = 8.3 Hz, 1H), 7.51–7.45 (m, 1H), 7.34 (d, *J* = 7.7 Hz, 2H), 6.99 (d, *J* = 3.5 Hz, 1H), 2.72 (p, *J* = 6.8 Hz, 2H), 1.17 (d, *J* = 6.8 Hz, 12H).

^{13}C NMR (101 MHz, $CDCl_3$): δ = 163.7, 163.4, 145.6, 145.2, 138.8, 138.7, 136.9, 133.9, 133.6, 133.2, 131.7, 131.0, 129.5, 124.3, 124.0, 123.6, 123.1, 120.8, 117.7, 117.0, 115.3, 108.2, 107.7, 29.1, 24.0 ppm

HRMS (ESI, pos. mode, $CHCl_3$/MeCN): m/z: 472.20466 $[M]^+$ (calcd. for $C_{31}H_{26}N_3O_2^+$: 472.20195).

N-(2,6-Diisopropylphenyl)-5,11b-diaza-11bH-benzo[hi]aceanthrylene-8,9-dicarboximide (1e)

Cyclohexane/ethyl acetate (4:1), orange solid, 56% yield. m.p. > 350 °C

^{1}H NMR (400 MHz, $CDCl_3$) δ 8.76 (d, *J* = 7.8 Hz, 1H), 8.68 (d, *J* = 7.8 Hz, 1H), 8.64 (d, *J* = 8.2 Hz, 1H), 8.57 (d, *J* = 5.3 Hz, 1H), 7.96 (d, *J* = 3.4 Hz, 1H), 7.63–7.57 (m, 2H), 7.50–7.46 (m, 1H), 7.34 (d, *J* = 7.7 Hz, 2H), 6.88 (d, *J* = 3.3 Hz, 1H), 2.74 (p, *J* = 6.8 Hz, 2H), 1.18 (d, *J* = 6.9 Hz, 12H).

^{13}C NMR (101 MHz, $CDCl_3$): δ = 163.8, 163.5, 145.6, 144.6, 138.4, 137.8, 135.5, 133.5, 133.5, 133.4, 131.5, 131.0, 130.1, 129.5, 125.0, 124.1, 124.0, 121.7, 119.0, 118.0, 116.9, 108.4, 107.7, 29.1, 24.0 ppm

HRMS (ESI, pos. mode, $CHCl_3$/MeCN): m/z: 472.20405 $[M]^+$ (calcd. for $C_{31}H_{26}N_3O_2^+$: 472.20195).

N-(2,6-Diisopropylphenyl)-3,11b-diaza-11bH-benzo[hi]aceanthrylene-8,9-dicarboximide (1f)

Cyclohexane/ethyl acetate (4:1), orange solid, 81% yield. m.p. > 350 °C

^{1}H NMR (400 MHz, $CDCl_3$) δ 8.73 (d, *J* = 7.8 Hz, 1H), 8.72 (br, 1H), 8.68 (d, *J* = 8.2 Hz, 1H), 8.24 (d, *J* = 7.8 Hz, 1H), 8.07 (d, *J* = 3.5 Hz, 1H), 7.76 (d, *J* = 5.1 Hz, 1H), 7.62 (d, *J* = 8.3 Hz, 1H), 7.50–7.46 (m, 1H), 7.34 (d, *J* = 7.6 Hz, 2H), 7.10 (d, *J* = 3.5 Hz, 1H), 2.71 (p, *J* = 6.8 Hz, 2H), 1.18 (d, *J* = 6.8 Hz, 12H).

^{13}C NMR (101 MHz, $CDCl_3$): δ = 163.6, 163.4, 148.3, 147.7, 145.5, 138.7, 134.0, 133.4, 132.9, 131.6, 130.9, 129.5, 125.9, 125.0, 125.0, 124.1, 123.6, 122.2, 119.6, 116.6, 111.3, 110.1, 107.7, 29.1, 24.0 ppm

HRMS (ESI, pos. mode, $CHCl_3$/MeCN): m/z: 472.20349 $[M]^+$ (calcd. for $C_{31}H_{26}N_3O_2^+$: 472.20195).

Synthesis of 1g–j

General procedure: aminodibromoarene (40.0 μmol, 1.00 equiv.), compound **3** (0.160 mmol, 4.00 equiv.), $Pd(PPh_3)_4$ (8.00 μmol, 0.200 equiv.), and potassium carbonate (0.240 mmol, 6.00 equiv.) were charged in a 15 mL Schlenk tube under an argon atmosphere. Then 0.8 mL toluene–EtOH–H_2O (5 : 2 : 1) was added into the flask via a syringe. The reaction mixture was heated at 90 °C under intense stirring and argon atmosphere for overnight. After cooled to room temperature, the reaction mixture was diluted with dichloromethane, washed with water and brine, dried over anhydride sodium sulfate, and filtered. After removal of solvent, the crude product was purified by column chromatography on silica-gel and precipitation from dichloromethane/cyclohexane to afford the product.

N,N'-Bis(2,6-diisopropylphenyl)-15b-aza-15b*H*-tribenzo[*de,hi,op*]tetracene-3,4:12,13-bis(dicarboximide) (1g)

Cyclohexane/ethyl acetate (3:1), purple solid, 62% yield. m.p. > 350 °C

^{1}H NMR (400 MHz, $CDCl_3$) δ 8.78 (d, *J* = 7.9 Hz, 2H), 8.59 (d, *J* = 8.5 Hz, 2H), 8.43 (d, *J* = 8.5 Hz, 2H), 8.26 (d, *J* = 8.0 Hz, 2H), 8.21 (d, *J* = 8.0 Hz, 2H), 7.64 (t, *J* = 7.9 Hz, 1H), 7.49 (t, *J* = 7.8 Hz, 2H), 7.35 (d, *J* = 7.8 Hz, 3h), 2.78–2.70 (m, 3h), 1.26–1.11 (m, 23h).

^{13}C NMR (101 MHz, $CDCl_3$): δ = 163.8, 163.2, 145.6, 141.1, 135.1, 134.1, 133.4, 132.4, 130.9, 130.7, 129.5, 126.5, 126.2, 124.4, 124.1, 123.6, 120.4, 117.1, 116.9, 112.2, 29.1, 24.0 ppm

HRMS (ESI, pos. mode, $CHCl_3$/MeCN): m/z: 822.33197 $[M]^+$ (calcd. for $C_{54}H_{45}N_3NaO_4^+$: 822.33023).

N,N'-Bis(2,6-diisopropylphenyl)-8,15b-diaza-15b*H*-tribenzo[*de,hi,op*]tetracene-3,4:12,13-bis(di-carboximide) (1h)

Cyclohexane/ethyl acetate (3:1), red solid, 44% yield. m.p. > 350 °C

^{1}H NMR (400 MHz, $CDCl_3$) δ 9.35 (s, 2H), 8.80 (d, *J* = 7.9 Hz, 2H), 8.63 (d, *J* = 8.5 Hz, 2H), 8.43 (d, *J* = 8.5 Hz, 2H), 8.31 (d, *J* = 8.1 Hz, 2H), 7.54–7.46 (m, 2H), 7.35 (d, *J* = 7.8 Hz, 3h), 2.78–2.70 (m, 3h), 1.19 (d, *J* = 6.8 Hz, 23h).

^{13}C NMR (101 MHz, $CDCl_3$): δ = 163.6, 163.0, 147.1, 145.5, 140.3, 140.1, 133.4, 132.6, 131.7, 130.7, 129.6, 124.9, 124.1, 124.1, 121.3, 118.0, 117.8, 116.9, 112.2, 29.2, 24.0 ppm

HRMS (ESI, pos. mode, $CHCl_3$/MeCN): m/z: 801.34538 $[M]^+$ (calcd. for $C_{53}H_{45}N_4O_4^+$: 801.34353).

N,N'-Bis(2,6-diisopropylphenyl)-7,15b-diaza-15b*H*-tribenzo[*de,hi,op*]tetracene-3,4:12,13-bis(di-carboximide) (1i)

Cyclohexane/ethyl acetate (3:1), purple solid, 67% yield. m.p. 323–324 °C.

^{1}H NMR (400 MHz, $CDCl_3$) δ 8.86–8.73 (m, 3h), 8.63 (dd, *J* = 13.4, 8.5 Hz, 2H), 8.55 (d, *J* = 8.5 Hz, 1H), 8.49 (d, *J* = 8.5 Hz, 1H), 8.25 (d, *J* = 8.0 Hz, 1H), 7.98 (d, *J* = 5.2 Hz, 1H), 7.53–7.46 (m, 2H), 7.36 (d, *J* = 7.8 Hz, 3h), 2.77–2.71 (m, 3h), 1.19 (dd, *J* = 7.1, 1.9 Hz, 23h).

^{13}C NMR (101 MHz, $CDCl_3$): δ = 163.8, 163.6, 163.1, 162.9, 146.7, 145.6, 145.5, 141.8, 140.4, 140.0, 134.3, 133.5, 133.1, 132.8, 132.2, 131.6, 131.3, 130.8, 130.7, 130.7, 130.6, 129.9, 129.6, 129.6, 125.1, 125.0, 124.1, 124.1, 122.3, 121.9, 119.8, 118.4, 118.2, 117.4, 117.1, 112.3, 111.8, 29.2, 29.2, 24.0 ppm

HRMS (ESI, pos. mode, $CHCl_3$/MeCN): m/z: 823.32871 $[M]^+$ (calcd. for $C_{53}H_{44}N_4NaO_4^+$: 823.32548).

N,N'-Bis(2,6-diisopropylphenyl)-7,9,15b-triaza-15b*H*-tribenzo[*de,hi,op*]tetracene-3,4:12,13-bis(di-carboximide) (1j)

Cyclohexane/ethyl acetate (1:1), purple solid, 62% yield. m.p. > 350 °C.

^{1}H NMR (400 MHz, $CDCl_3$) δ 9.27 (s, 1H), 8.90–8.78 (m, 3h), 8.66 (d, *J* = 8.5 Hz, 2H), 8.60 (d, *J* = 8.5 Hz, 2H), 7.54–7.43 (m, 2H), 7.36 (d, *J* = 7.8 Hz, 3h), 2.77–2.70 (m, 3h), 1.19 (dd, *J* = 6.8, 0.9 Hz, 23h).

^{13}C NMR (101 MHz, $CDCl_3$) δ =163.53, 162.83, 155.17, 147.53, 145.51, 139.25, 133.32, 132.60, 132.13, 130.61, 130.59, 129.69, 129.16, 125.98, 124.15, 123.71, 120.82, 117.71, 111.72, 29.20, 24.01, 23.99 ppm

HRMS (ESI, pos. mode, $CHCl_3$/MeCN): m/z: 824.31699 $[M]^+$ (calcd. for $C_{52}H_{43}N_5NaO_4^+$: 824.32073).

Synthesis of *N*-(2,6-diisopropylphenyl)-4-(carbazol-1-yl)-5-chloronaphthalene-1,8-dicarboximide (7)

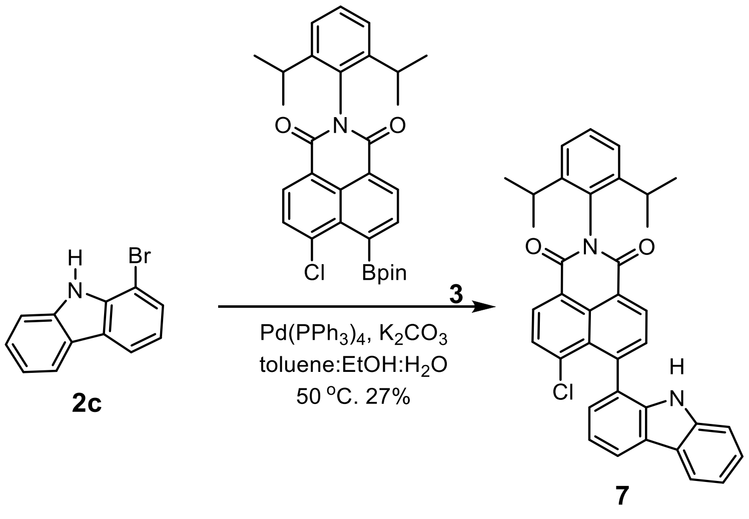

1-Bromo-9*H*-carbazole **2c** (50.0 mg, 0.200 mmol, 1.00 equiv.), compound **3** (210 mg, 0.400 mmol, 2.00 equiv.), $Pd(PPh_3)_4$ (24.0 mg, 20.0 μmol, 0.100 equiv.), and potassium carbonate (85.0 mg, 0.600 mmol, 3.00 equiv.) were charged in a 15 mL Schlenk tube under an argon atmosphere. Then 4 mL toluene–EtOH–H_2O (5 : 2 : 1) was added into the flask via a syringe. The reaction mixture was heated at 50 °C under intense stirring and argon atmosphere for four hours. After cooled to room temperature, the reaction mixture was diluted with dichloromethane, washed with water and brine, dried over anhydride sodium sulfate, and filtered. After removal of solvent, the crude product was purified by column chromatography on silica-gel (cyclohexane/ethyl acetate 10:1) and precipitation from dichloromethane/cyclohexane to afford the product 30 mg as yellow solid (27%).

m.p. 198–199 °C

^{1}H NMR (400 MHz, $CDCl_3$) δ 8.83 (d, J = 7.5 Hz, 1H), 8.64 (d, J = 7.9 Hz, 1H), 8.20–8.18 (m, 1H), 8.17–8.14 (m, 1H), 7.93 (d, J = 7.5 Hz, 1H), 7.81 (d, J = 7.9 Hz, 1H), 7.64 (s, 1H), 7.53–7.49 (m, 1H), 7.45–7.34 (m, 5H), 7.31–7.28 (m, 2H), 2.98–2.55 (m, 2H), 1.25–1.15 (m, 12H).

^{13}C NMR (101 MHz, $CDCl_3$) δ 163.61, 163.53, 145.60, 145.46, 142.63, 139.60, 139.51, 138.14, 132.35, 131.99, 131.93, 131.34, 130.62, 130.37, 129.73, 127.76, 126.17, 126.12, 124.58, 124.16, 124.09, 123.28, 123.26, 123.15, 122.13, 120.57, 120.37, 119.85, 119.49, 110.76, 29.25, 29.22, 23.99, 23.98, 23.96.

HRMS (ESI, pos. mode, $CHCl_3$/MeCN): m/z: 579.18304 $[M]^+$ (calcd. for $C_{36}H_{29}ClN_2NaO_2^+$: 579.18089).

Conversion of intermediate 7 to 1c

Compound **7** (8.50 mg, 0.150 mmol, 1.00 equiv.), potassium carbonate (6.50 mg, 0.450 mmol, 3.00 equiv.), were charged in a 15 mL Schlenk tube under an argon atmosphere. Then 0.4 mL toluene–EtOH–H_2O (5 : 2 : 1) was added into the flask via a syringe. The reaction mixture was heated at 90 °C under intense stirring and argon atmosphere for four hours. The reaction mixture was cooled to room temperature and 20 mL dichloromethane and 20 mL water was added. The mixture was extracted with dichloromethane. The combined organic layers were washed with water, dried over $MgSO_4$ and concentrated under reduced pressure. The product was dried under high vacuum to give **1c** (87%, NMR yield using $CHCl_2CHCl_2$ as an internal standard). ^{1}H NMR spectrum was in accordance with product identity of one-pot procedure.

3. Optical properties

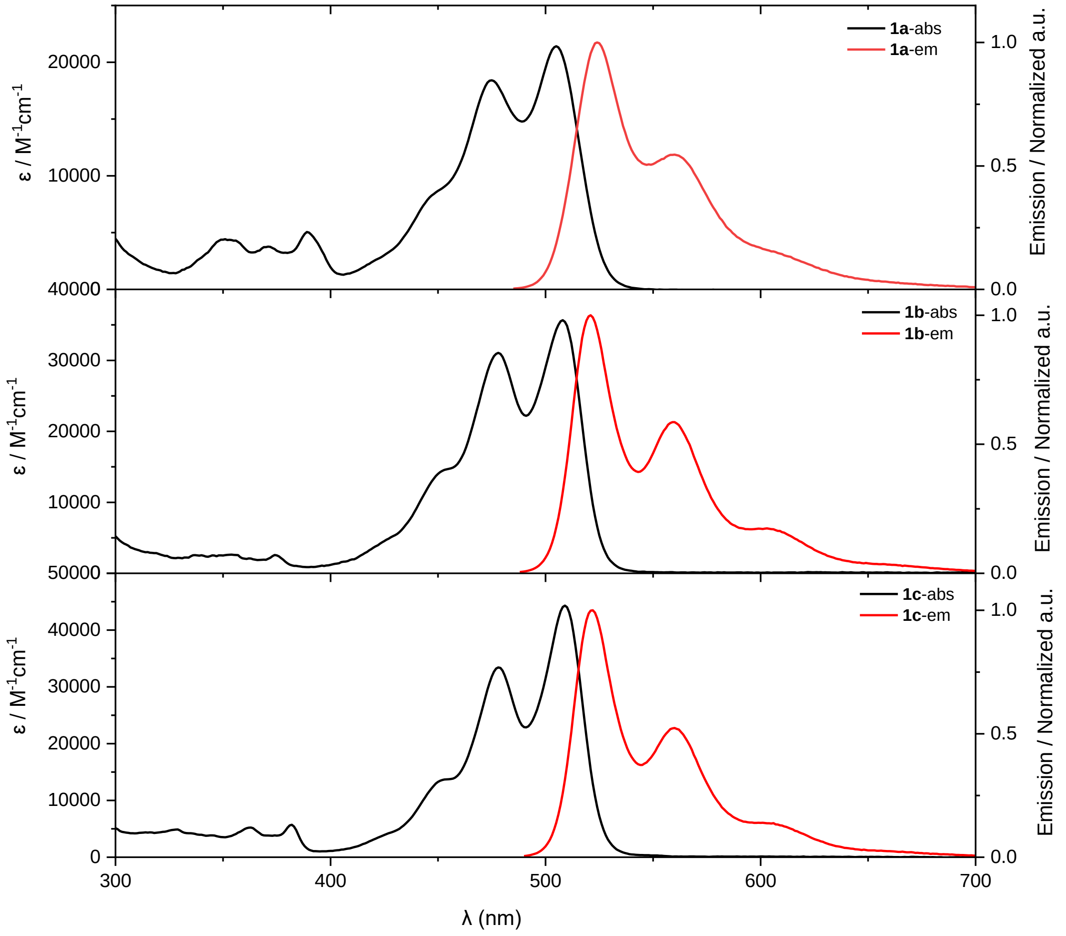

Figure S1. UV/Vis absorption (Black, $c = 4 \times 10^{-6}$ M) and emission spectra (Red, λ_{ex} = 475 nm for **1a**, 477 nm for **1b**, 478 nm for **1c**, OD ~ 0.05) of **1a**–**c** in chloroform at 293 K.

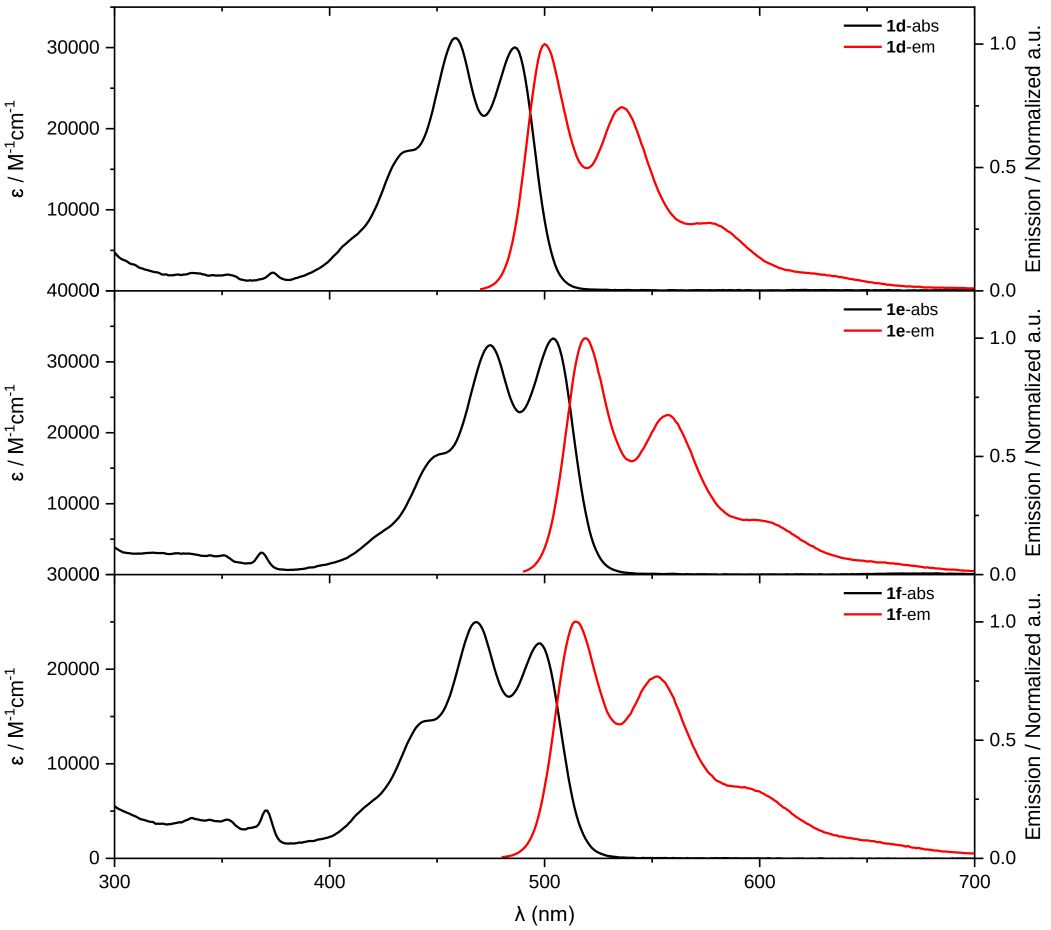

Figure S2. UV/Vis absorption (Black, $c = 4 \times 10^{-6}$ M) and emission spectra (Red, λ_{ex} = 458 nm for **1d**, 474 nm for **1e**, 468 nm for **1f**, OD ~ 0.05) of **1d**–**f** in chloroform at 293 K.

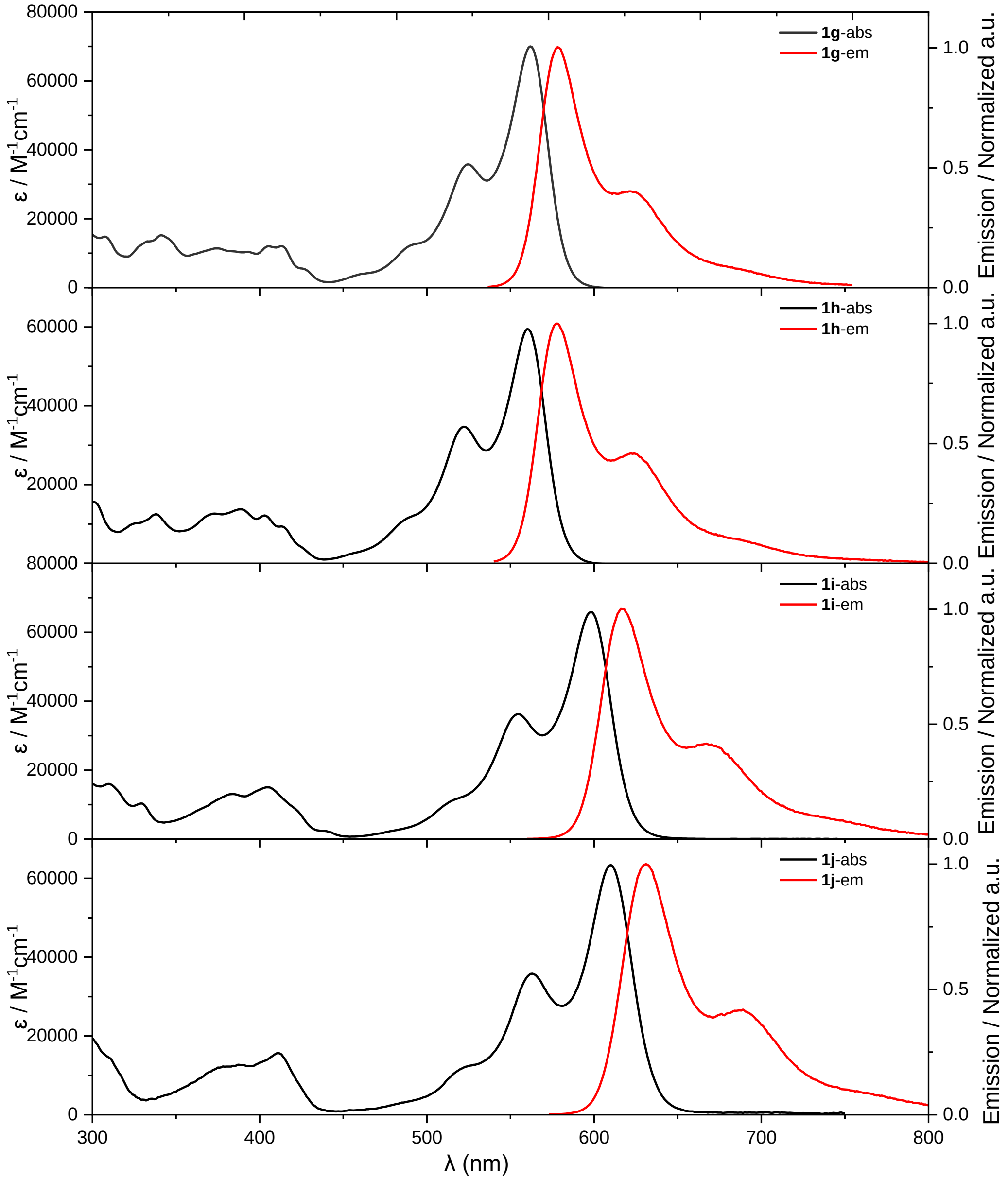

Figure S3.UV/Vis absorption (Black, c = 4 × 10^{-6} M) and emission spectra (Red, λ_{ex} = 547 nm for **1g**, 522 nm for **1h**, 555 nm for **1i**, 563 nm for **1j**, OD ~ 0.05) of **1g**–**j** in chloroform at 293 K.

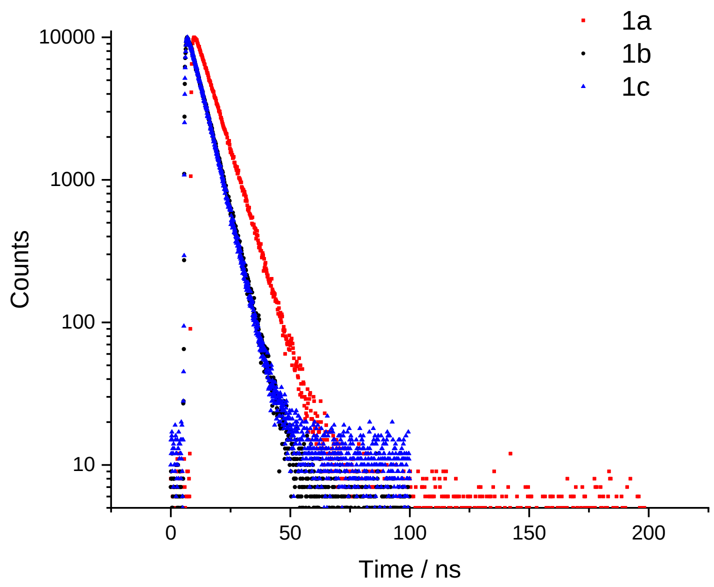

Figure S4. Fluorescence decay of **1a**–**c** (**1a**: red, λ_{ex} = 480 nm, λ_{em} = 524 nm. **1b**: black, λ_{ex} = 480 nm, λ_{em} = 521 nm. **1c**: blue, λ_{ex} = 480 nm, λ_{em} = 522 nm) in chloroform at 293 K.

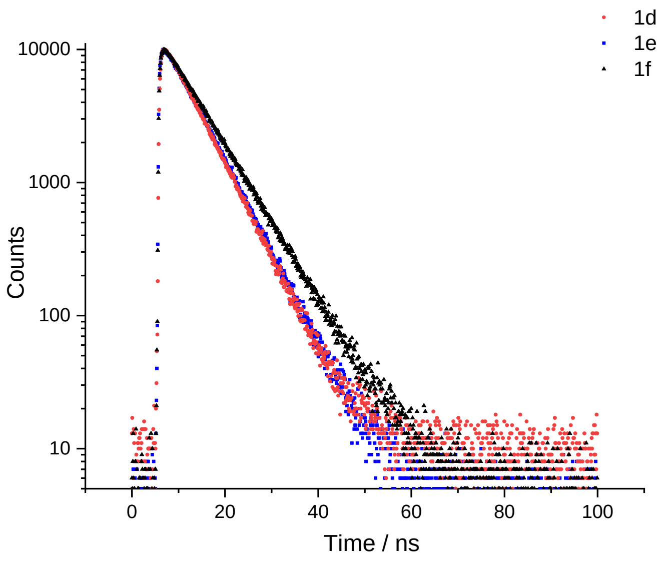

Figure S5. Fluorescence decay of **1d**–**f** (**1d**: red, λ_{ex} = 480 nm, λ_{em} = 500 nm. **1e**: blue, λ_{ex} = 480 nm, λ_{em} = 519 nm. **1f**: black, λ_{ex} = 480 nm, λ_{em} = 515 nm) in chloroform at 293 K.

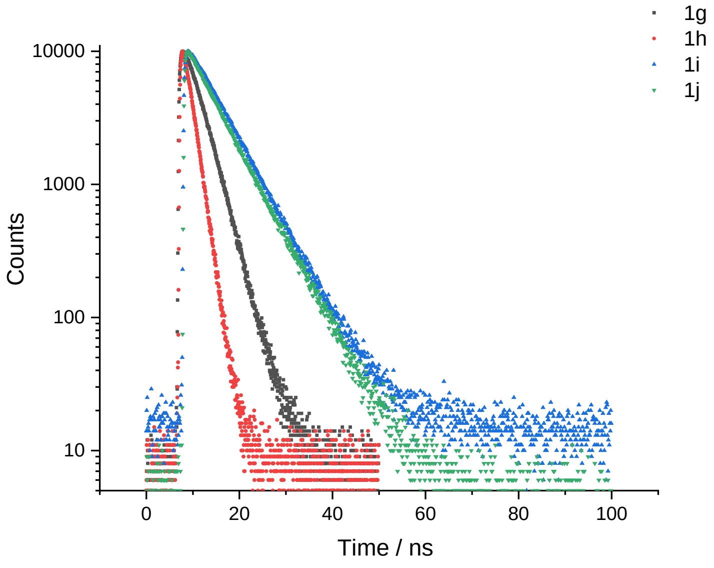

Figure S6. Fluorescence decay of **1g**–**j** (**1g**: black, λ_{ex} = 506 nm, λ_{em} = 607 nm. **1h**: red, λ_{ex} = 506 nm, λ_{em} = 578 nm. **1i**: blue, λ_{ex} = 506 nm, λ_{em} = 617 nm. **1j**: green, λ_{ex} = 506 nm, λ_{em} = 631 nm) in chloroform at 293 K.

Table S1. Summary of the Optical Properties of Compounds **1a**–**j.**

Compd.	$\lambda_{abs,}$ (nm)[a]	λ_{em} (nm)[a]	Stokes shift [cm^{-1}]	ε [M^{-1}cm^{-1}]	Φ_{fl} [%][b]	t [ns] (χ^2)[c]
**1a**	505	524	718	21400	94	7.73 (1.22)
**1b**	508	521	491	35700	99	6.06 (1.24)
**1c**	509	522	489	44300	99	5.93 (1.19)
**1d**	486	500	576	31200	99	6.14 (1.15)
**1e**	504	519	574	33300	99	6.34 (1.26)
**1f**	497	515	703	25000	99	7.46 (1.24)
**1g**	588	607	532	70000	38	3.14 (1.26)
**1h**	560	578	556	59500	23	1.74 (1.25)
**1i**	598	617	515	65900	84	6.49 (1.14)
**1j**	610	631	546	63400	65	6.47 (1.12)

[a] UV–vis absorption and fluorescence measurements were performed using chloroform solution ($c \sim 4 \times 10^{-6}$ M for UV–vis, ~1×10^{-6} M for fluorescence) at 293 K. Excitation wavelengths for steady-state fluorescence measurements are 475 nm for **1a**, 477 nm for **1b**, 478 nm for **1c**, 458 nm for **1d**, 474 nm for **1e**, 468 nm for **1f**, 547 nm for **1g**, 522 nm for **1h**, 555 nm for **1i**, and 563 nm for **1j**. [b] Absolute method using an integral sphere in chloroform solution. [c] The fit quality parameter.

4. Electrochemical properties

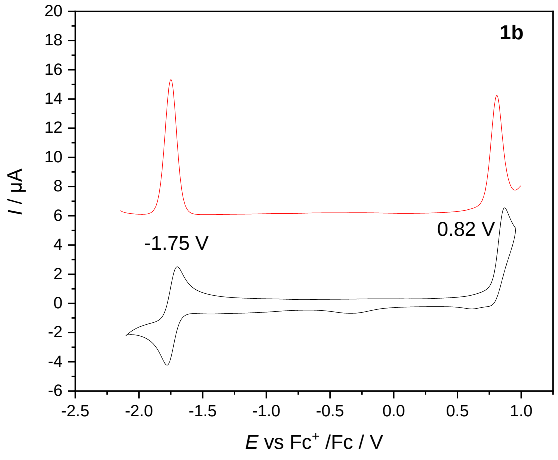

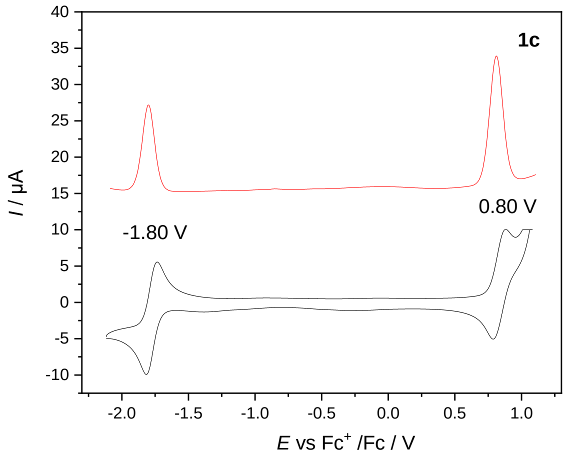

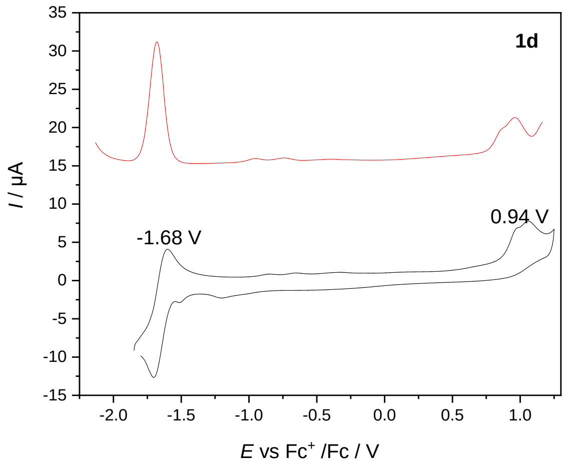

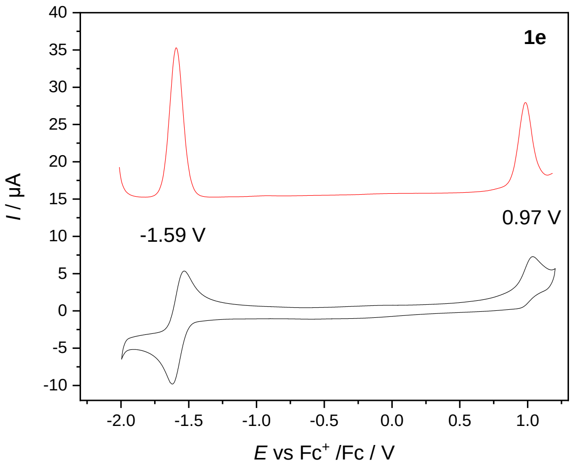

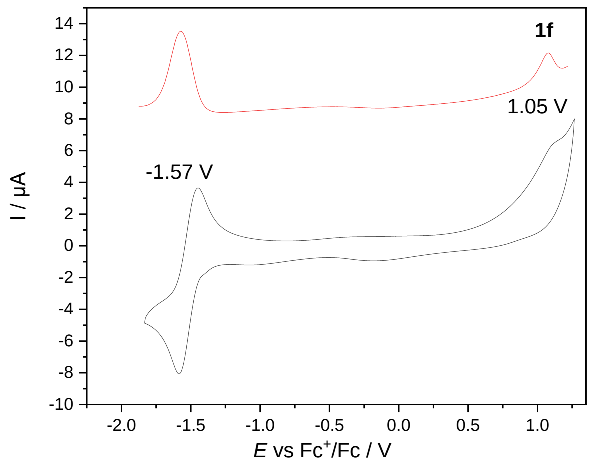

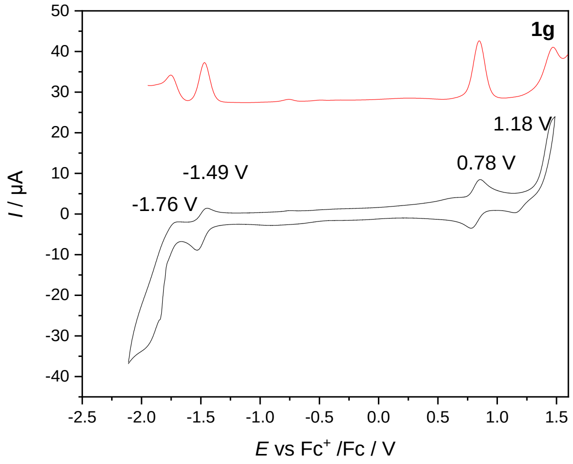

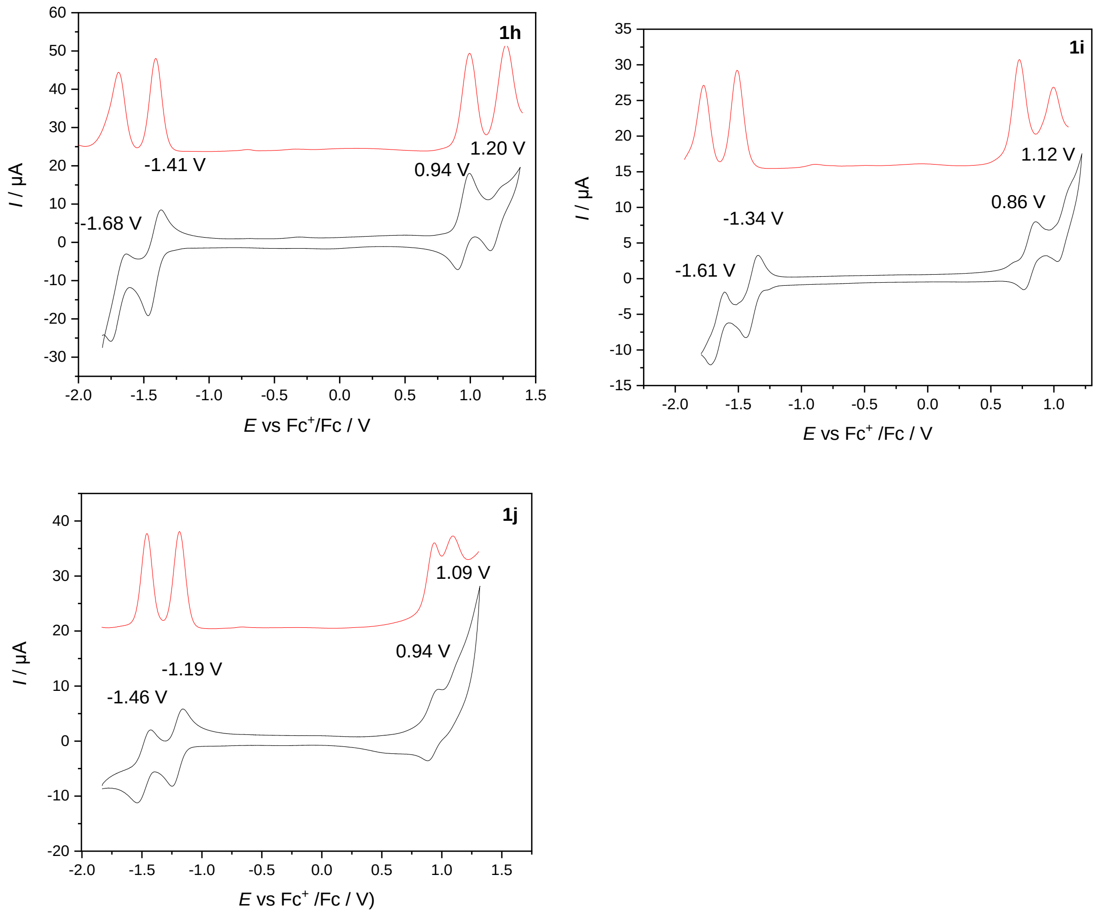

Figure S7. Cyclic (black line) and square (red line) voltammetry traces of **1b**–**j** in dichloromethane solutions ($c \sim 10^{-4}$ M) with TBAHFP (0.1 M) as a supporting electrolyte at room temperature (scan rate 100 mV s^{-1}).

Table S2. Summary of the Redox Properties of Compounds **1b**–**j.**

Compd.	E_{ox1} [V][a]	E_{ox2} [V][a]	E_{red1} [V][a]	E_{red2} [V][a]	E_{HOMO} [eV][b]	E_{LUMO} [eV][b]	E_{gap} [eV]
**1b**	0.82 [c]	n.d.	-1.75	n.d.	-5.97	-3.40	2.57
**1c**	0.80	n.d.	-1.80	n.d.	-5.95	-3.35	2.60
**1d**	0.94 [c]	n.d.	-1.68	n.d.	-6.09	-3.47	2.62
**1e**	0.97 [c]	n.d.	-1.59	n.d.	-6.12	-3.56	2.56
**1f**	1.05 [c]	n.d.	-1.57	n.d.	-6.20	-3.58	2.62
**1g**	0.78	1.18 [c]	-1.49	-1.78 [c]	-5.93	-3.66	2.27
**1h**	0.94	1.20 [c]	-1.41	-1.68	-6.09	-3.74	2.35
**1i**	0.86	1.12 [c]	-1.34	-1.61	-6.01	-3.81	2.20
**1j**	0.94	1.09 [c]	-1.19	-1.46	-6.09	-3.96	2.13
PMI[d]	0.95	n.d.	-1.46	-1.95	-6.10	-3.69	2.41

[a] Half-wave potentials were determined by cyclic voltammetry measured in dichloromethane (0.1 M TBAHFP) vs. Fc^+/Fc. [b] Calculated according to a literature known procedure using the experimentally determined redox potentials ($E_{LUMO} = -[E(M/M^-) + 5.15 \text{ eV}]$ and $E_{HOMO} = -[E(M/M^+) + 5.15 \text{ eV}]$) and the energy level of Fc^+/Fc with respect to the vacuum level (−5.15 eV). [c] pseudo-reversible or irreversible (half-wave potentials were determined by square wave voltammetry). [d] data from ref S10 and S11. n.d.: not determined.

5. Theoretical calculations

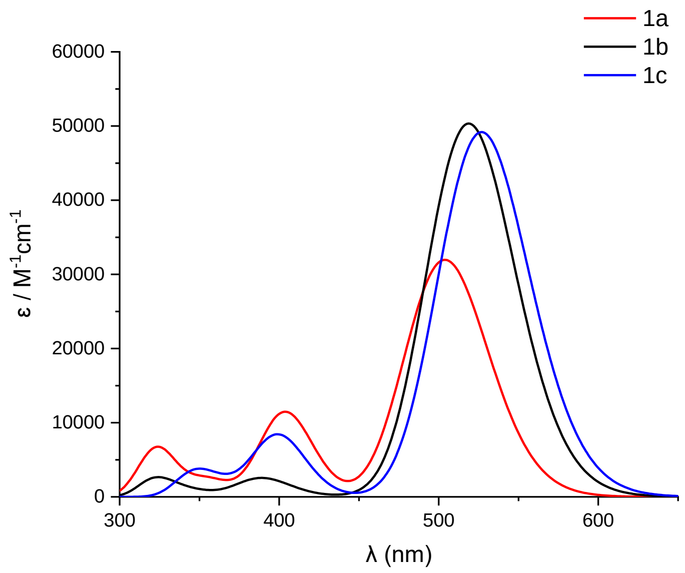

Figure S8. Calculated UV/vis spectra of **1a**–**c** at B3LYP/6-31G(d) (half width 0.15 eV, The calculated spectra were shifted by 0.43 eV towards lower energies).

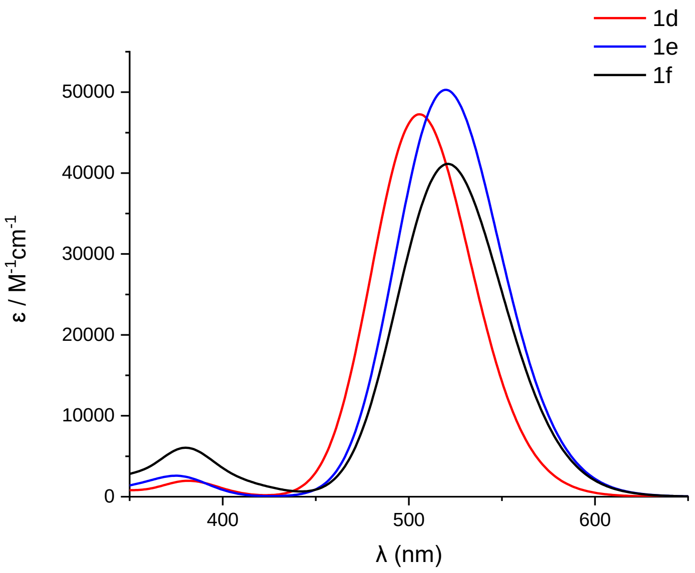

Figure S9. Calculated UV/vis spectra of **1d**–**f** at B3LYP/6-31G(d) level of theory (half width 0.15 eV, The calculated spectra were shifted by 0.43 eV towards lower energies).

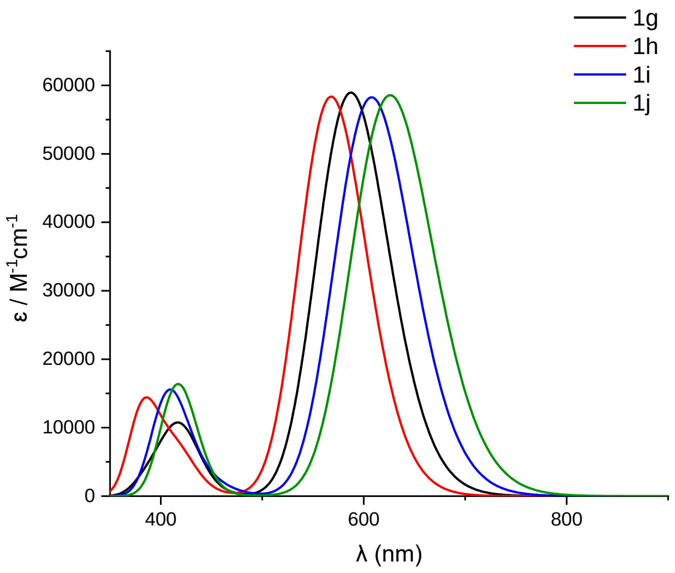

Figure S10. Calculated UV/vis spectra of **1g**–**j** at B3LYP/6-31G(d) level of theory (half width 0.15 eV, The calculated spectra were shifted by 0.2 eV towards lower energies).

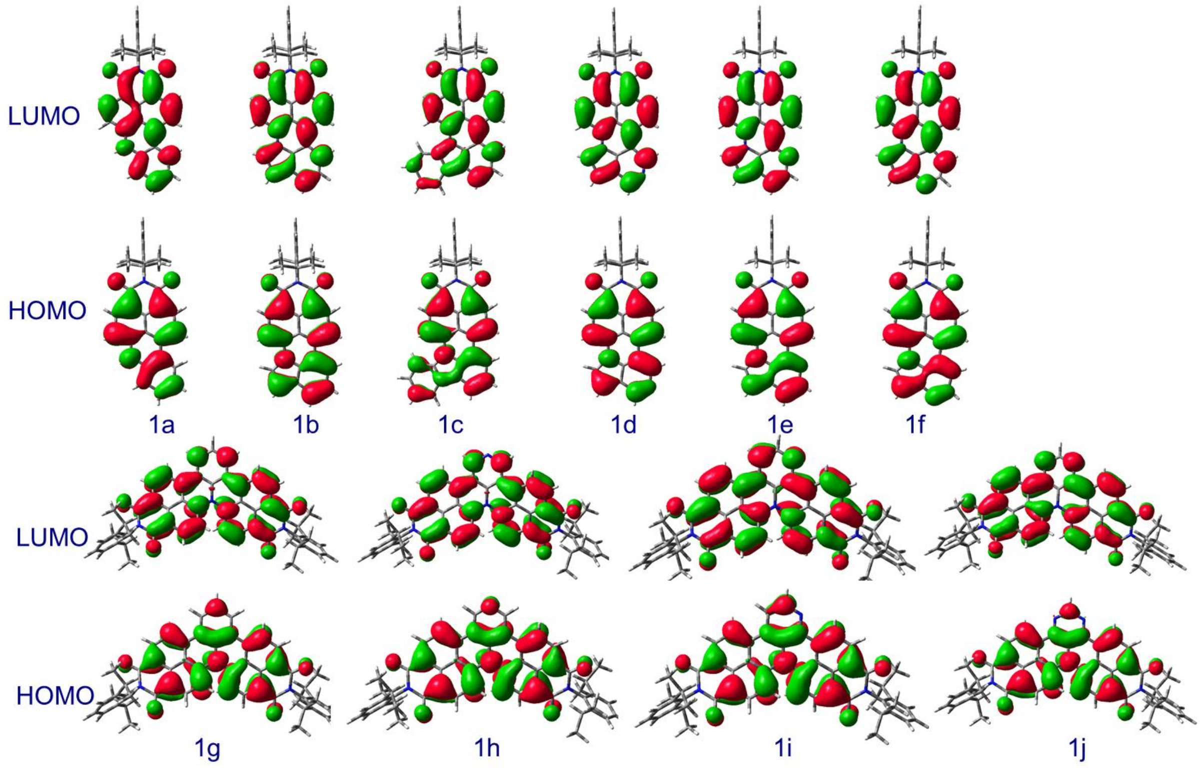

Figure S11. Calculated frontier orbitals (HOMO left; LUMO right) at B3LYP/6-31G(d) level of theory.

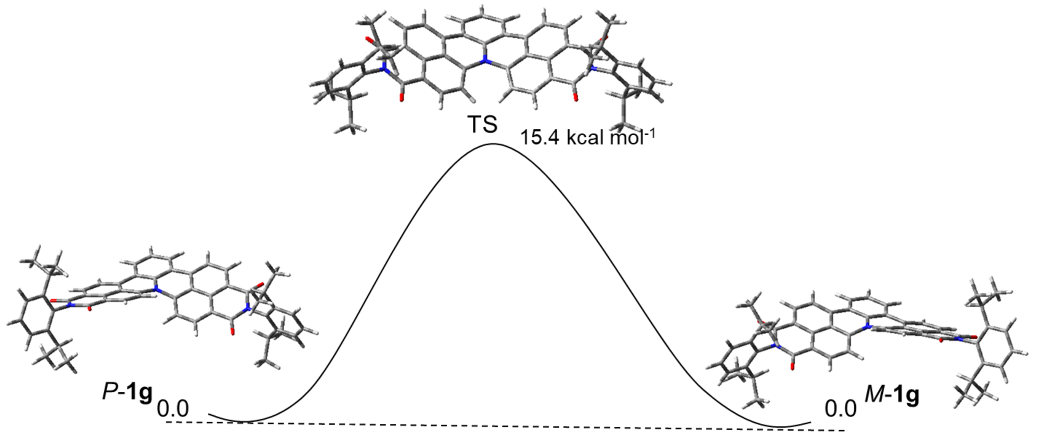

Figure S12. Activation barrier for the isomerization process between *P*-**1g** and *M*-**1g** in units of kcal/mol. The relative Gibbs free energy was calculated at the B3LYP/6-31G(d) level of theory at 298K.

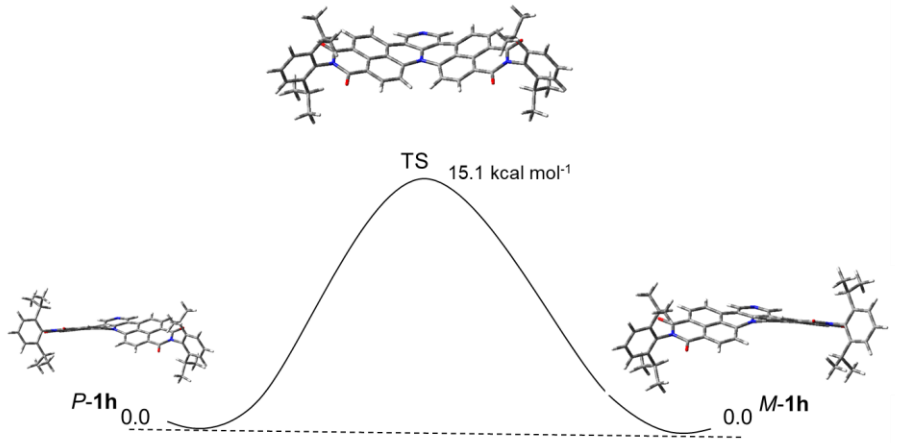

Figure S13. Activation barrier for the isomerization process between *P*-**1h** and *M*-1h in units of kcal/mol. The relative Gibbs free energy was calculated at the B3LYP/6-31G(d) level of theory at 298K.

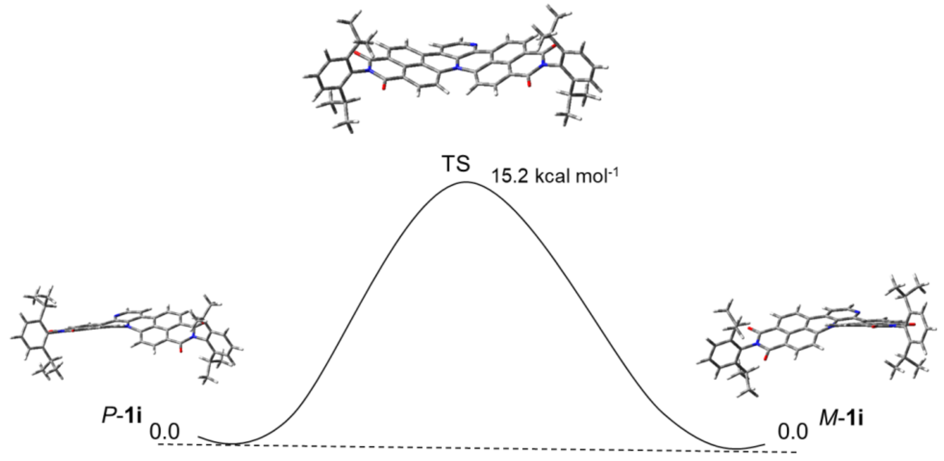

Figure S14. Activation barrier for the isomerization process between *P*-**1i** and *M*-**1i** in units of kcal/mol. The relative Gibbs free energy was calculated at the B3LYP/6-31G(d) level of theory at 298K.

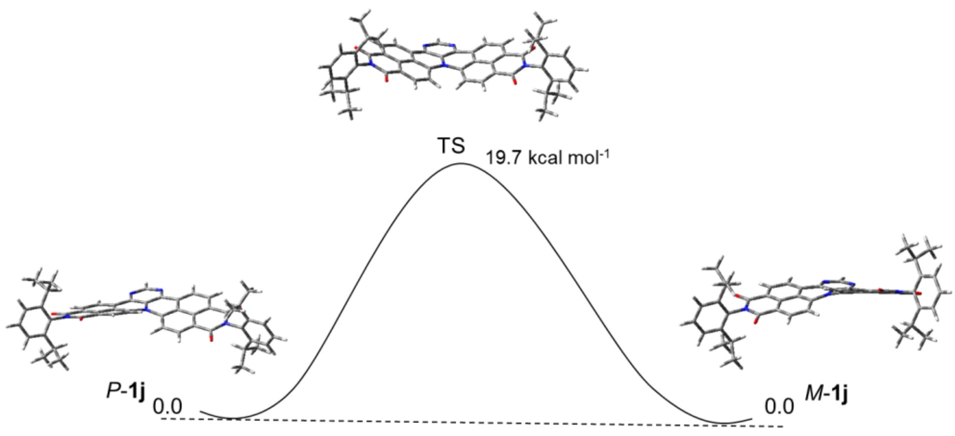

Figure S15. Activation barrier for the isomerization process between *P*-**1j** and *M*-**1j** in units of kcal/mol. The relative Gibbs free energy was calculated at the B3LYP/6-31G(d) level of theory at 298K.

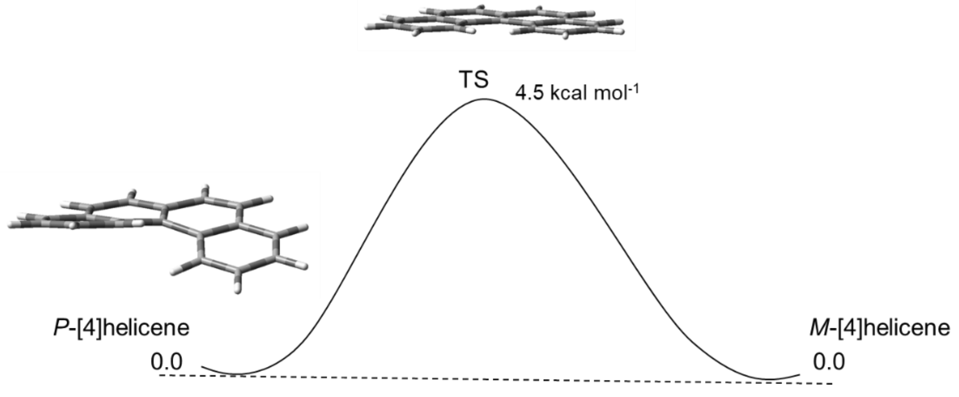

Figure S16. Activation barrier for the isomerization process between *P*-[4]helicene and *M*-[4]helicene in units of kcal/mol. The relative Gibbs free energy was calculated at the B3LYP/6-31G(d) level of theory at 298K.

6. Single crystal X-ray analysis

Table S3. Crystal data and structure refinement for **1c**.

CCDC Number	2190559	
Empirical formula	$C_{36}H_{28}N_2O_2$	
Formula weight	520.60	
Temperature	100(2) K	
Wavelength	1.54178 Å	
Crystal system	Monoclinic	
Space group	$P2_1/n$	
Unit cell dimensions	a = 8.2004(5) Å	α = 90°.
	b = 31.668(2) Å	β = 99.250(3)°.
	c = 10.2040(7) Å	γ = 90°.
Volume	2615.4(3) $Å^3$	
Z	4	
Density (calculated)	1.322 g/cm^3	
Absorption coefficient	0.644 mm^{-1}	
F(000)	1096	
Crystal size	0.339 × 0.049 × 0.027 mm^3	
Theta range for data collection	2.791 to 80.619°.	
Index ranges	$-10 \leq h \leq 10$, $-39 \leq k \leq 40$, $-12 \leq l \leq 9$	
Reflections collected	28140	
Independent reflections	5569 [R(int) = 0.0653]	
Completeness to theta = 67.679°	99.6%	
Absorption correction	Semi-empirical from equivalents	
Max. and min. transmission	0.7543 and 0.5764	
Refinement method	Full-matrix least-squares on F^2	
Data / restraints / parameters	5569 / 0 / 365	
Goodness-of-fit on F^2	1.152	
Final R indices [$I > 2\sigma(I)$]	R_1 = 0.0981, wR_2 = 0.2526	
R indices (all data)	R_1 = 0.1092, wR_2 = 0.2588	
Extinction coefficient	n/a	
Largest diff. peak and hole	0.741 and –0.267 e·$Å^{-3}$	

Table S4. Crystal data and structure refinement for **1g**.

CCDC Number	2190560	
Empirical formula	$C_{54}H_{45}N_3O_4$	
Formula weight	892.06	
Temperature	100(2) K	
Wavelength	1.54178 Å	
Crystal system	Triclinic	
Space group	$P\bar{1}$	
Unit cell dimensions	a = 16.9580(11) Å	α = 104.311(2)°.
	b = 17.1701(11) Å	β = 109.776(2)°.
	c = 17.8837(11) Å	γ = 90.172(2)°.
Volume	4726.6(5) $Å^3$	
Z	4	
Density (calculated)	1.254 g/cm^3	
Absorption coefficient	0.614 mm^{-1}	
F(000)	1888	
Crystal size	0.305 × 0.288 × 0.120 mm^3	
Theta range for data collection	2.668 to 80.556°.	
Index ranges	$-21 \le h \le 21$, $-21 \le k \le 21$, $-22 \le l \le 22$	
Reflections collected	20332	
Independent reflections	20332 [R(int) = 0.0433]	
Completeness to theta = 67.679°	99.9%	
Absorption correction	Semi-empirical from equivalents	
Max. and min. transmission	0.7543 and 0.5941	
Refinement method	Full-matrix least-squares on F^2	
Data / restraints / parameters	20332 / 24 / 1265	
Goodness-of-fit on F^2	1.061	
Final R indices [$I > 2\sigma(I)$]	R_1 = 0.0725, wR_2 = 0.1982	
R indices (all data)	R_1 = 0.0752, wR_2 = 0.1998	
Extinction coefficient	n/a	
Largest diff. peak and hole	0.427 and –0.323 e·Å^{-3}	

Table S5. Crystal data and structure refinement for **1h**.

CCDC Number	2190561	
Empirical formula	$C_{53}H_{44}N_4O_4$	
Formula weight	893.05	
Temperature	100(2) K	
Wavelength	1.54178 Å	
Crystal system	Triclinic	
Space group	*P*1	
Unit cell dimensions	a = 8.3457(3) Å	α = 95.160(2)°.
	b = 9.3455(3) Å	β = 99.293(2)°.
	c = 17.6411(6) Å	γ = 115.715(2)°.
Volume	1203.41(7) $Å^3$	
Z	1	
Density (calculated)	1.232 g/cm^3	
Absorption coefficient	0.610 mm^{-1}	
F(000)	472	
Crystal size	0.214 × 0.139 × 0.023 mm^3	
Theta range for data collection	2.580 to 80.575°.	
Index ranges	$10 \leq h \leq 10$, $11 \leq k \leq 11$, $22 \leq l \leq 21$	
Reflections collected	21931	
Independent reflections	8928 [R(int) = 0.0644]	
Completeness to theta = 67.679°	99.9%	
Absorption correction	Semi-empirical from equivalents	
Max. and min. transmission	0.7543 and 0.5839	
Refinement method	Full-matrix least-squares on F^2	
Data / restraints / parameters	8928 / 3 / 622	
Goodness-of-fit on F^2	1.055	
Final R indices [$I > 2\sigma(I)$]	R_1 = 0.0684, wR_2 = 0.1687	
R indices (all data)	R_1 = 0.0796, wR_2 = 0.1819	
Absolute structure parameter	0.3(2)	
Extinction coefficient	n/a	
Largest diff. peak and hole	0.491 and –0.311 $e \cdot Å^{-3}$	

Table S6. Crystal data and structure refinement for **1i**.

CCDC Number	2190562	
Empirical formula	$C_{53}H_{44}N_4O_4$	
Formula weight	893.05	
Temperature	100(2) K	
Wavelength	1.54178 Å	
Crystal system	Triclinic	
Space group	$P\bar{1}$	
Unit cell dimensions	a = 16.7768(15) Å	α = 104.872(6)°.
	b = 17.5515(16) Å	β = 90.047(5)°.
	c = 17.6585(16) Å	γ = 109.465(6)°.
Volume	4717.0(8) Å^3	
Z	4	
Density (calculated)	1.258 g/cm^3	
Absorption coefficient	0.622 mm^{-1}	
F(000)	1888	
Crystal size	0.150 × 0.096 × 0.022 mm^3	
Theta range for data collection	2.601 to 68.813°.	
Index ranges	$-20 \le h \le 20$, $-20 \le k \le 20$, $-21 \le l \le 21$	
Reflections collected	16662	
Independent reflections	16662 [R(int) = 0.1105]	
Completeness to theta = 67.679°	97.5%	
Absorption correction	Semi-empirical from equivalents	
Max. and min. transmission	0.7528 and 0.5530	
Refinement method	Full-matrix least-squares on F^2	
Data / restraints / parameters	16662 / 0 / 1247	
Goodness-of-fit on F^2	1.059	
Final R indices [$I > 2\sigma(I)$]	R_1 = 0.1799, wR_2 = 0.4843	
R indices (all data)	R_1 = 0.2027, wR_2 = 0.4979	
Extinction coefficient	n/a	
Largest diff. peak and hole	0.713 and –0.721 e·Å^{-3}	

Table S7. Crystal data and structure refinement for **1j**.

CCDC Number	2190556	
Empirical formula	$C_{52}H_{43}N_5O_4$	
Formula weight	894.05	
Temperature	100(2) K	
Wavelength	0.61992 Å	
Crystal system	Monoclinic	
Space group	$P2_1/c$	
Unit cell dimensions	a = 21.815(2) Å	α = 90°.
	b = 24.526(3) Å	β = 95.275(3)°.
	c = 8.429(2) Å	γ = 90°.
Volume	4490.7(13) Å^3	
Z	4	
Density (calculated)	1.322 g/cm^3	
Absorption coefficient	0.063 mm^{-1}	
F(000)	1888	
Crystal size	0.200 × 0.050 × 0.050 mm^3	
Theta range for data collection	0.818 to 27.869°.	
Index ranges	$-28 \le h \le 28$, $-36 \le k \le 36$, $-12 \le l \le 12$	
Reflections collected	79085	
Independent reflections	12553 [R(int) = 0.0416]	
Completeness to theta = 21.836°	99.1%	
Absorption correction	None	
Refinement method	Full-matrix least-squares on F^2	
Data / restraints / parameters	12553 / 0 / 622	
Goodness-of-fit on F^2	1.088	
Final R indices [$I > 2\sigma(I)$]	R_1 = 0.0536, wR_2 = 0.1517	
R indices (all data)	R_1 = 0.0647, wR_2 = 0.1642	
Extinction coefficient	n/a	
Largest diff. peak and hole	0.627 and –0.387 e·Å^{-3}	

Table S8. Crystal data and structure refinement for **1g** and [4]helicene.

CCDC Number	2190563	
Empirical formula	$C_{72}H_{57}N_3O_4$	
Formula weight	1120.34	
Temperature	100(2) K	
Wavelength	0.61991 Å	
Crystal system	Triclinic	
Space group	$P\bar{1}$	
Unit cell dimensions	a = 8.202(9) Å	α = 90.012(4)°.
	b = 14.428(5) Å	β = 85.889(11)°.
	c = 24.669(10) Å	γ = 90.023(14)°.
Volume	2912(3) Å^3	
Z	2	
Density (calculated)	1.278 g/cm^3	
Absorption coefficient	0.060 mm^{-1}	
F(000)	1184	
Crystal size	0.200 × 0.200 × 0.050 mm^3	
Theta range for data collection	1.231 to 28.045°.	
Index ranges	$-11 \le h \le 11$, $-18 \le k \le 18$, $-29 \le l \le 30$	
Reflections collected	51217	
Independent reflections	14090 [R(int) = 0.0548]	
Completeness to theta = 21.836°	90.2%	
Absorption correction	None	
Refinement method	Full-matrix least-squares on F^2	
Data / restraints / parameters	14090 / 137 / 823	
Goodness-of-fit on F^2	1.040	
Final R indices [$I > 2\sigma(I)$]	R_1 = 0.1799, wR_2 = 0.5023	
R indices (all data)	R_1 = 0.2304, wR_2 = 0.5420	
Extinction coefficient	n/a	
Largest diff. peak and hole	0.488 and –0.511 e·Å^{-3}	

7. NMR spectra of new compounds

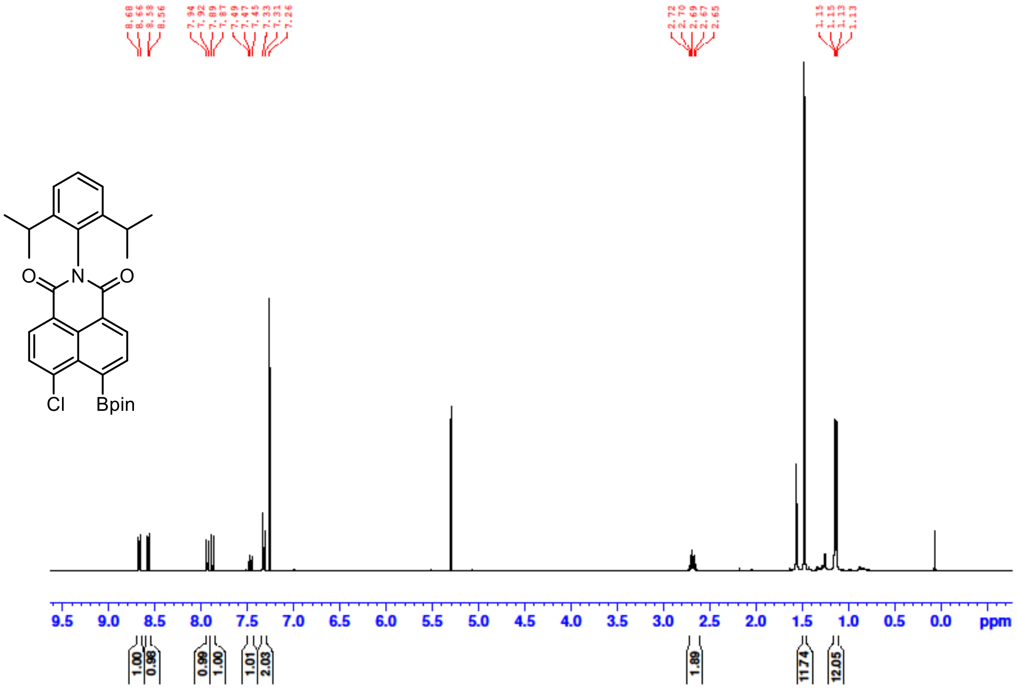

Figure S17. ^{1}H NMR (400 MHz) of **3** in $CDCl_3$ recorded at 298 K.

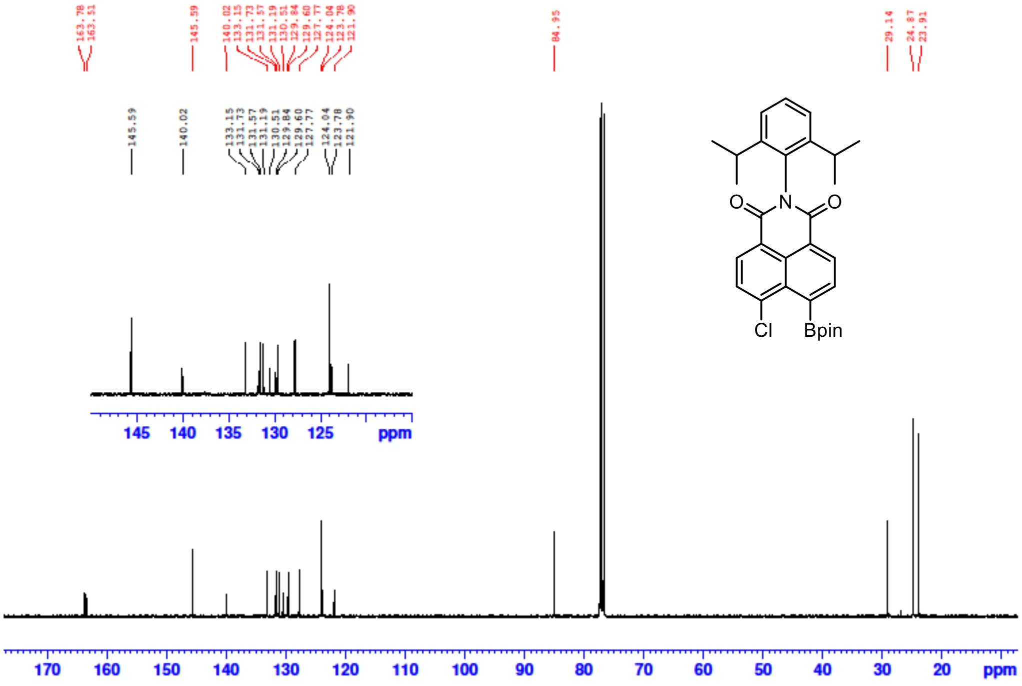

Figure S18. ^{13}C NMR (101 MHz) of **3** in $CDCl_3$ recorded at 298 K.

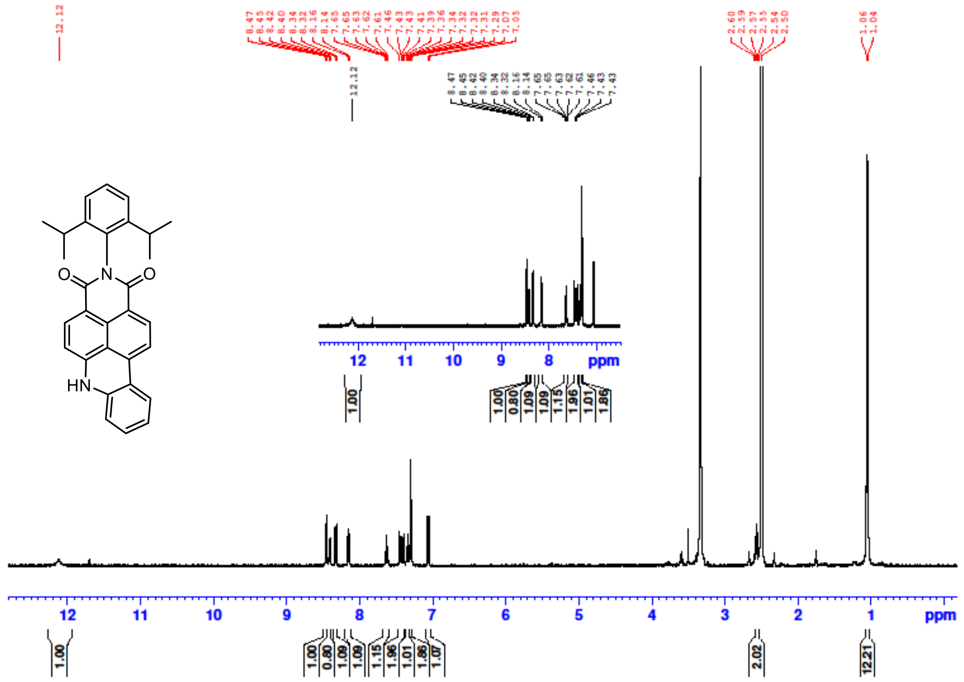

Figure S19. ^{1}H NMR (400 MHz) of **1a** in DMSO-d_6 recorded at 298 K.

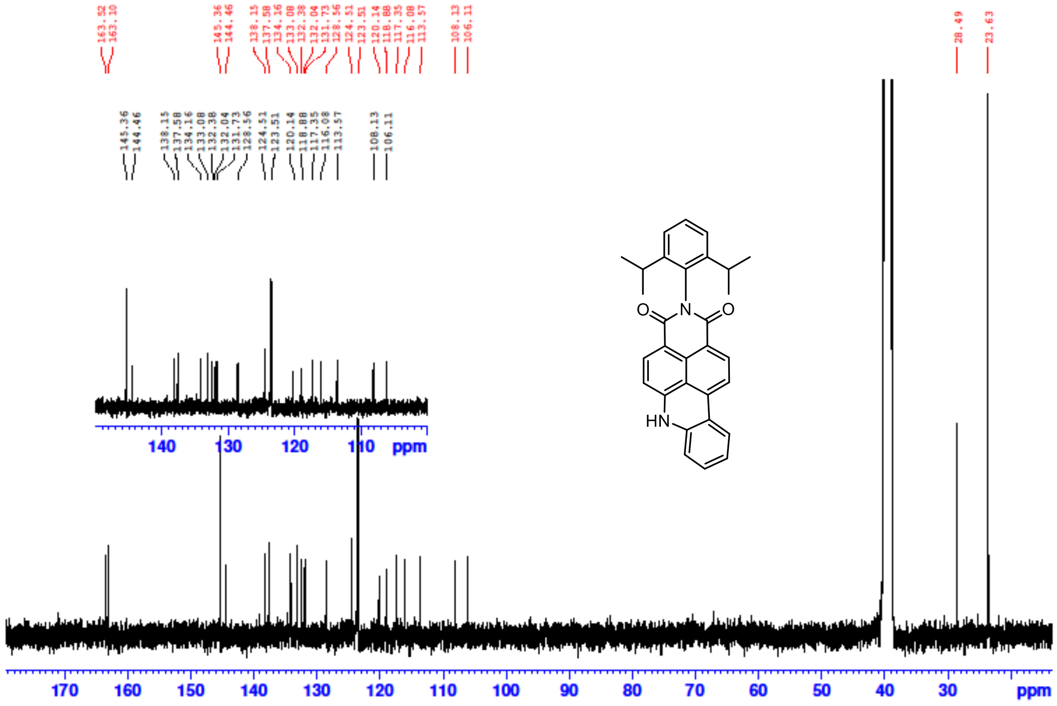

Figure S20. ^{13}C NMR (101 MHz) of **1a** in DMSO-d_6 recorded at 298 K.

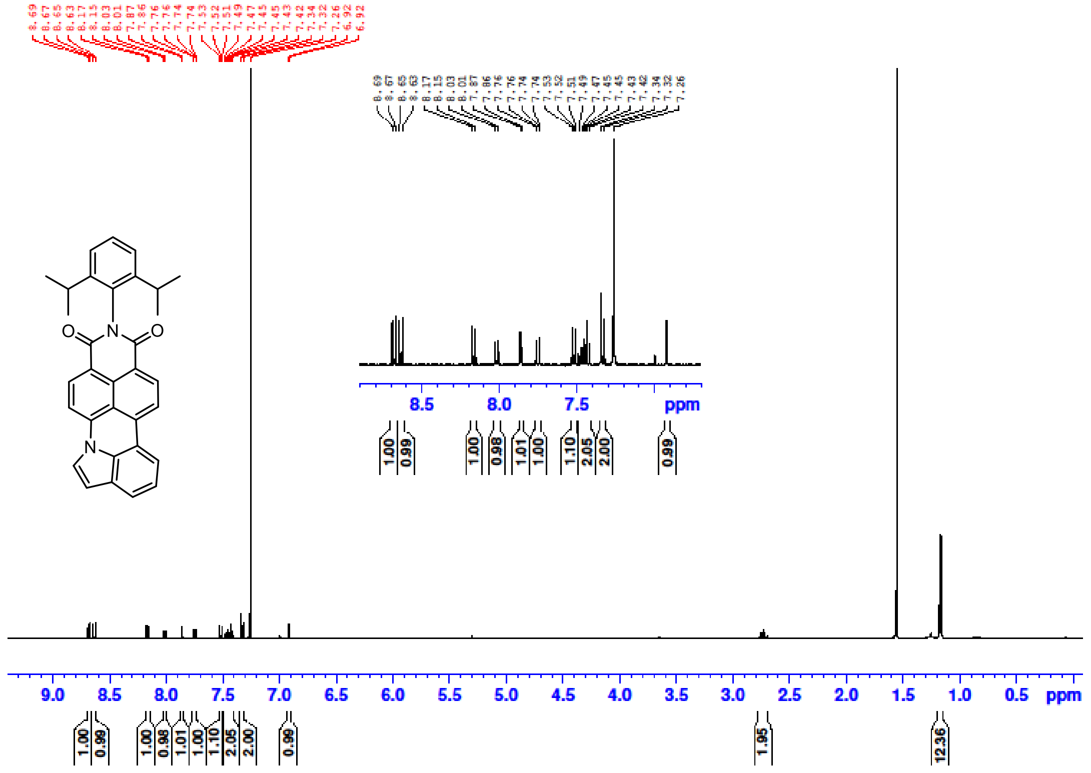

Figure S21. ^{1}H NMR (400 MHz) of **1b** in $CDCl_3$ recorded at 298 K.

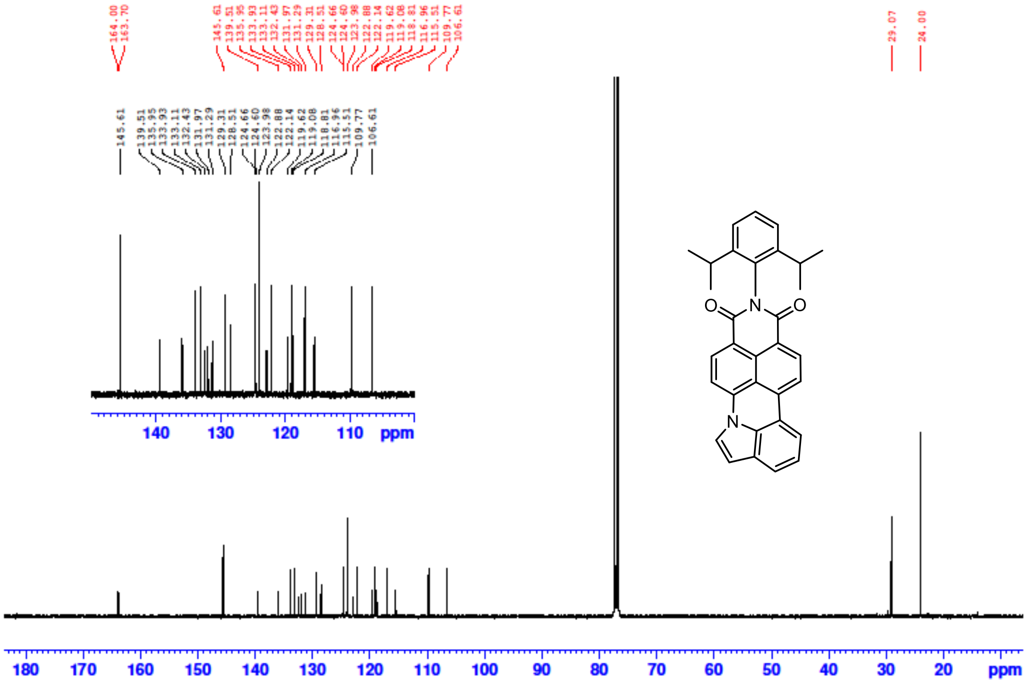

Figure S22. ^{13}C NMR (101 MHz) of **1b** in $CDCl_3$ recorded at 298 K.

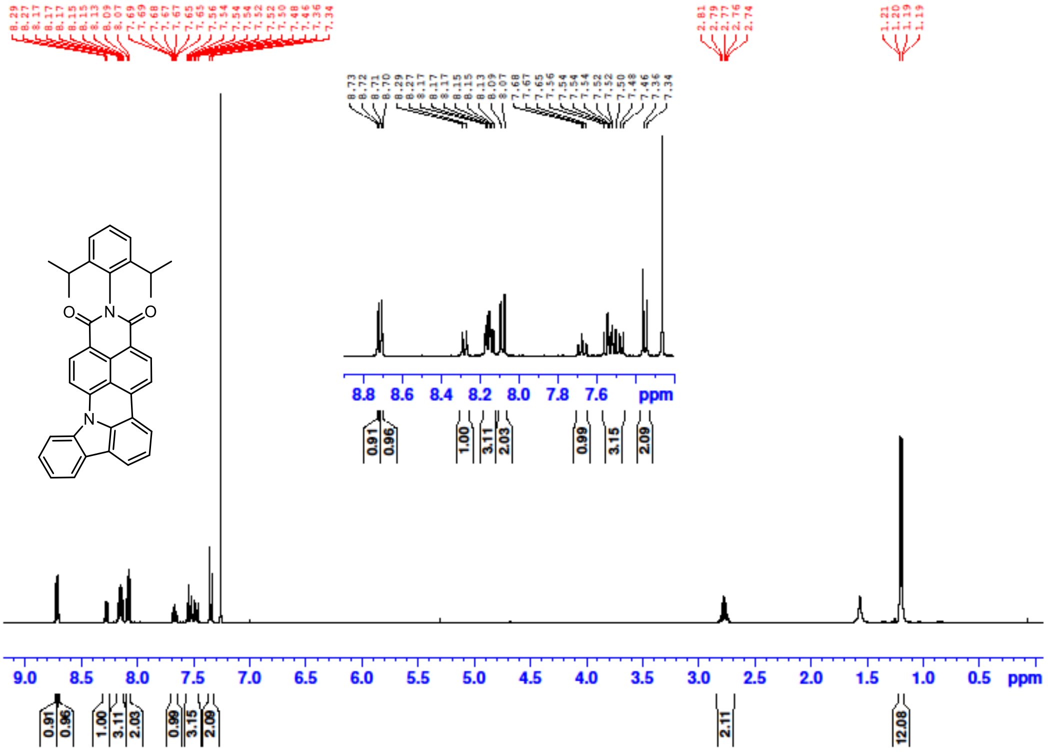

Figure S23. ^{1}H NMR (400 MHz) of **1c** in $CDCl_3$ recorded at 298 K.

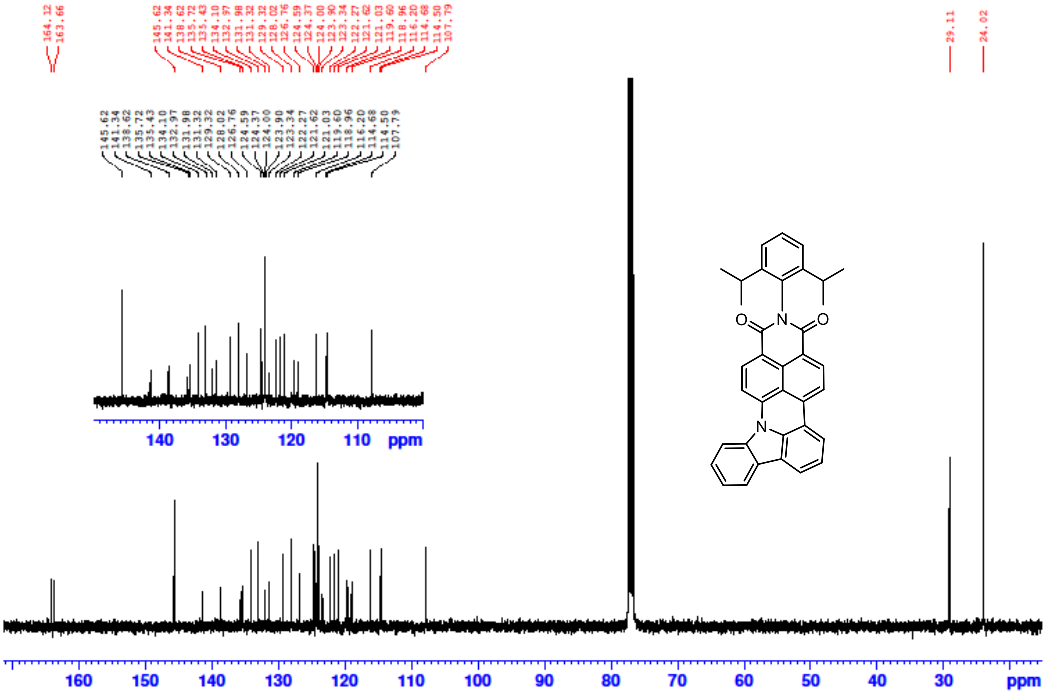

Figure S24. ^{13}C NMR (101 MHz) of **1c** in $CDCl_3$ recorded at 298 K.

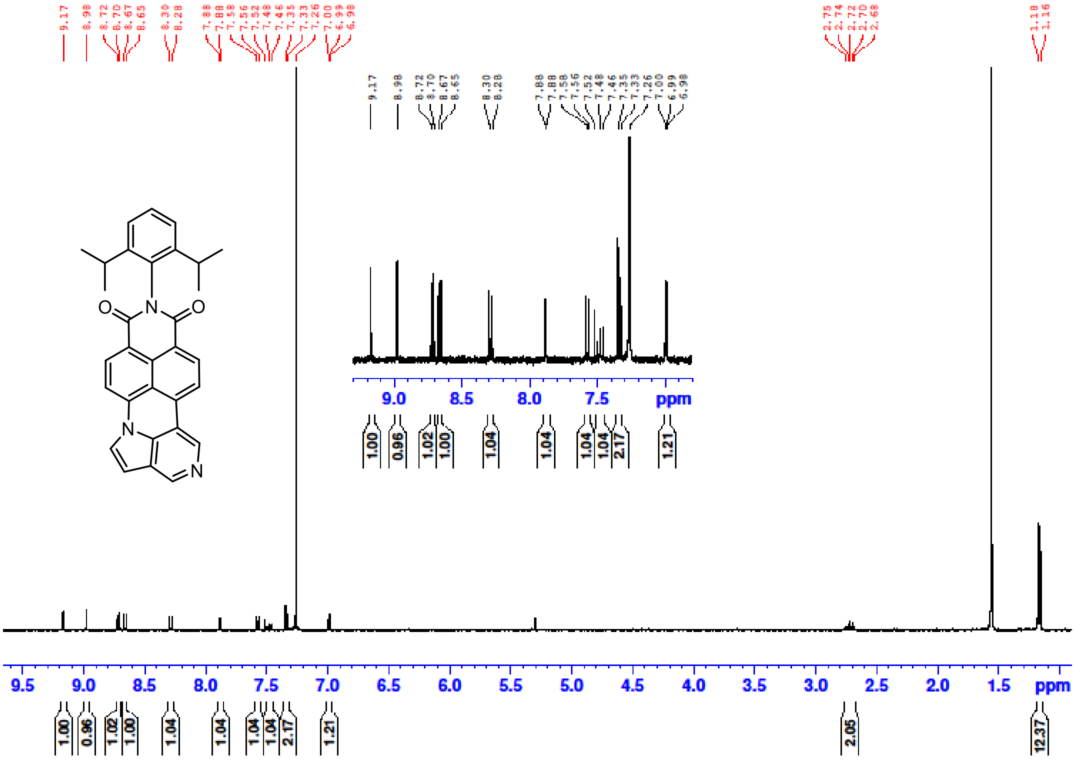

Figure S25. ^{1}H NMR (400 MHz) of **1d** in $CDCl_3$ recorded at 298 K.

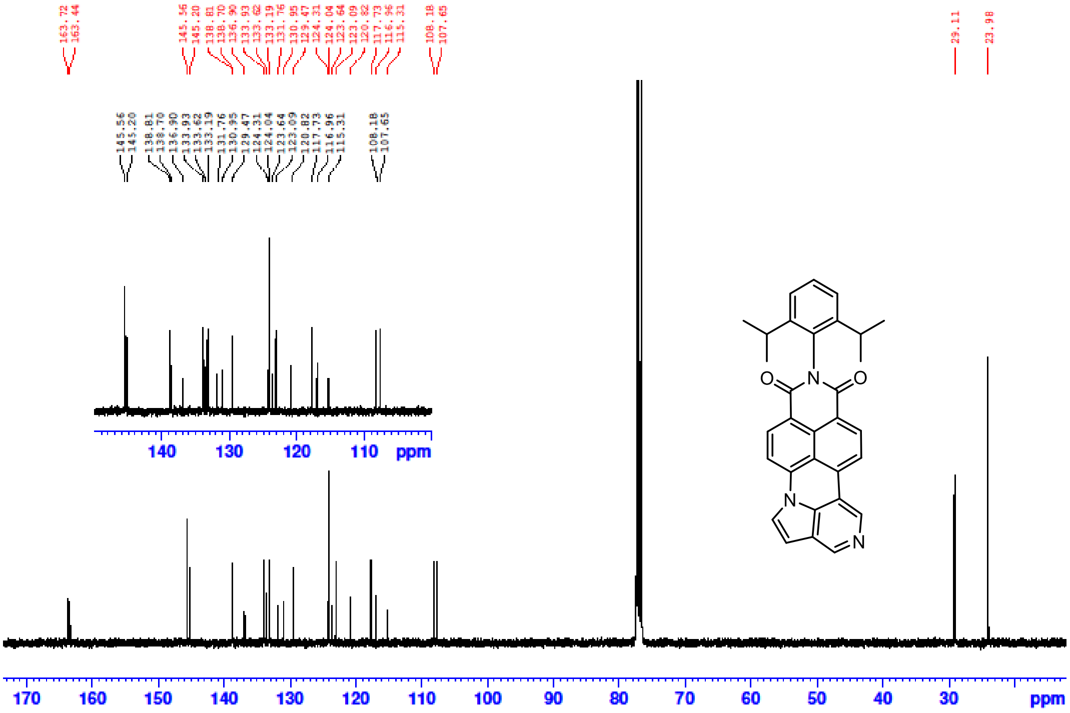

Figure S26. ^{13}C NMR (101 MHz) of **1d** in $CDCl_3$ recorded at 298 K.

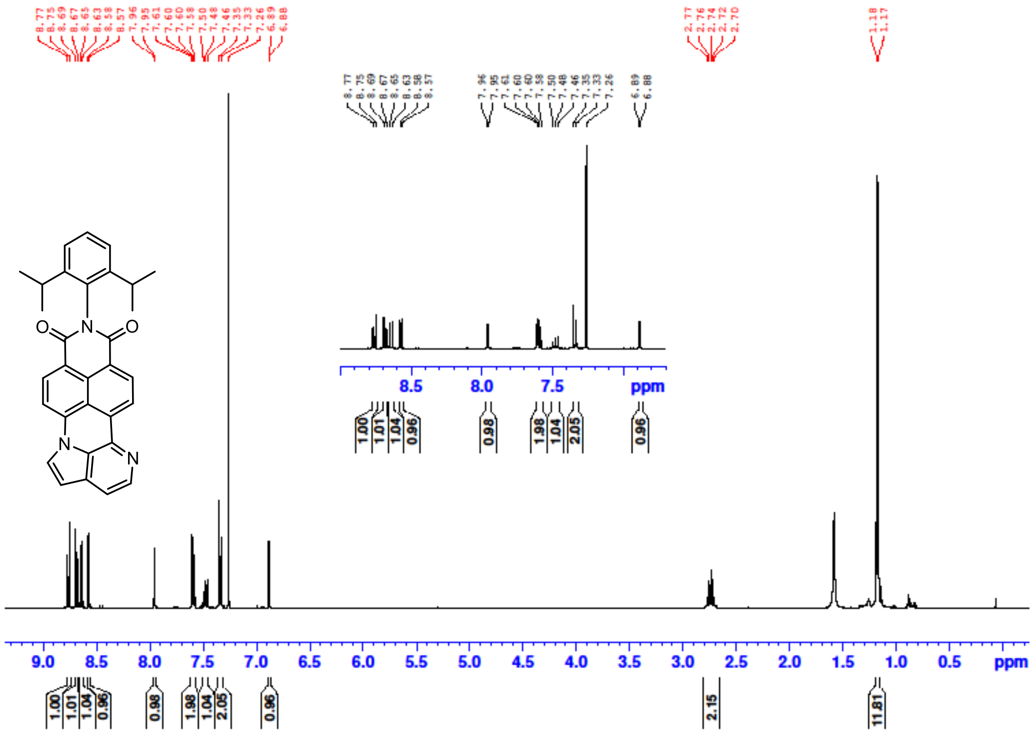

Figure S27. ^{1}H NMR (400 MHz) of **1e** in $CDCl_3$ recorded at 298 K.

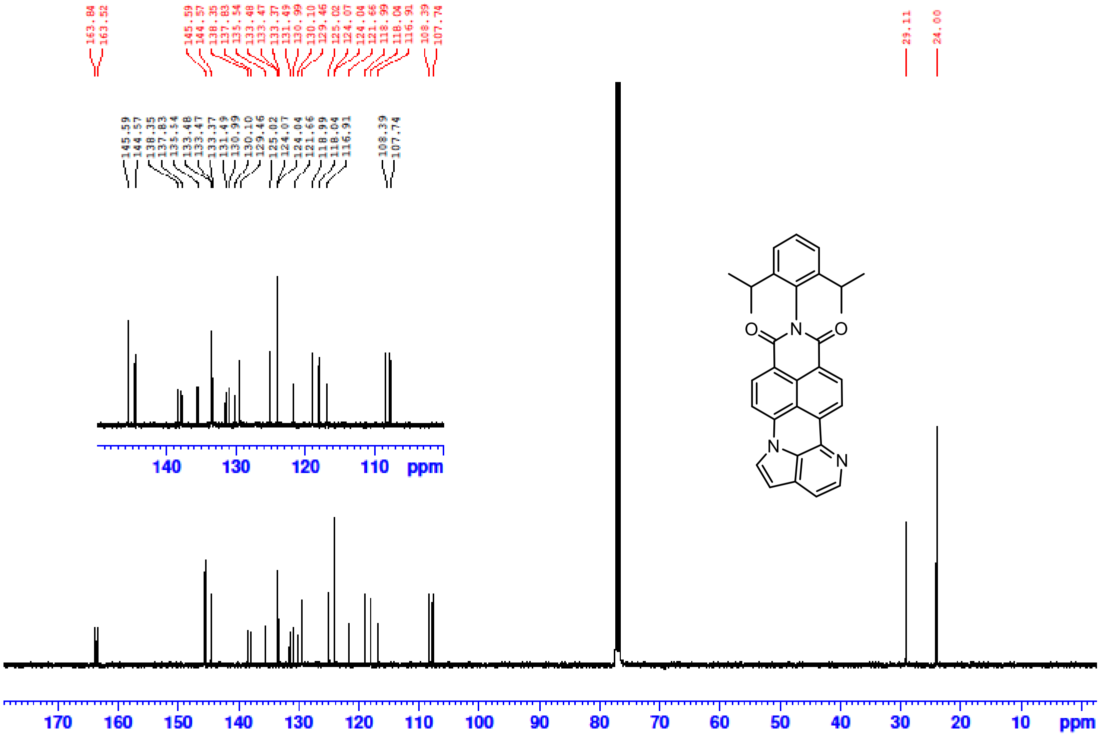

Figure S28. ^{13}C NMR (101 MHz) of **1e** in $CDCl_3$ recorded at 298 K.

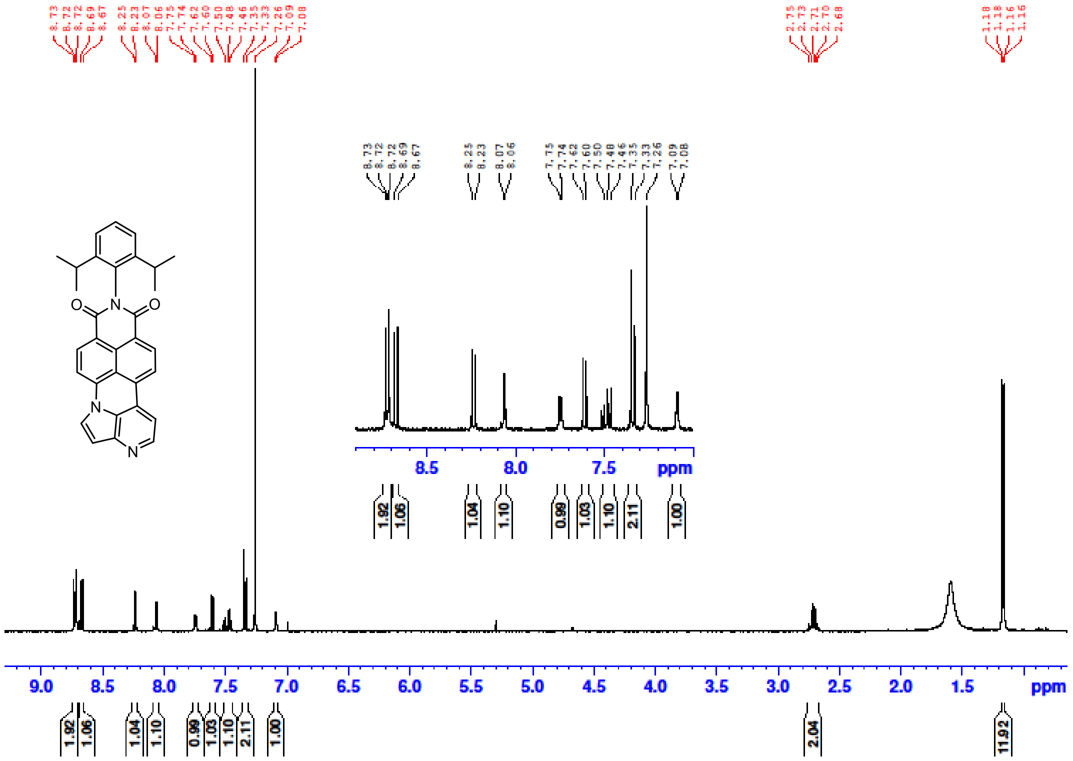

Figure S29. ^{1}H NMR (400 MHz) of **1f** in $CDCl_3$ recorded at 298 K.

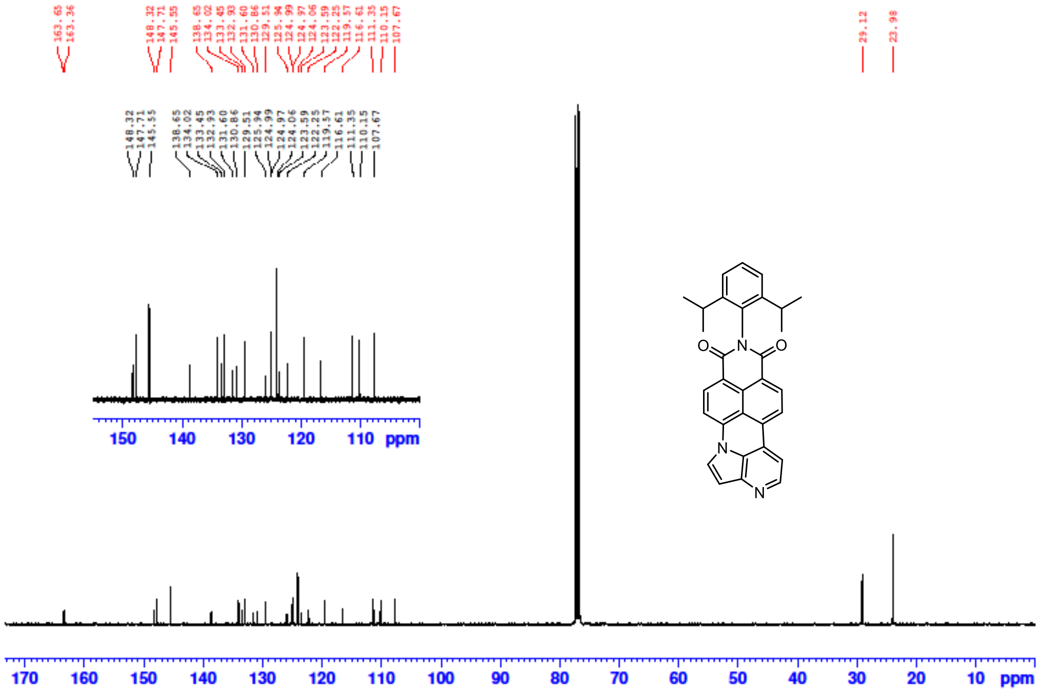

Figure S30. ^{13}C NMR (101 MHz) of **1f** in $CDCl_3$ recorded at 298 K.

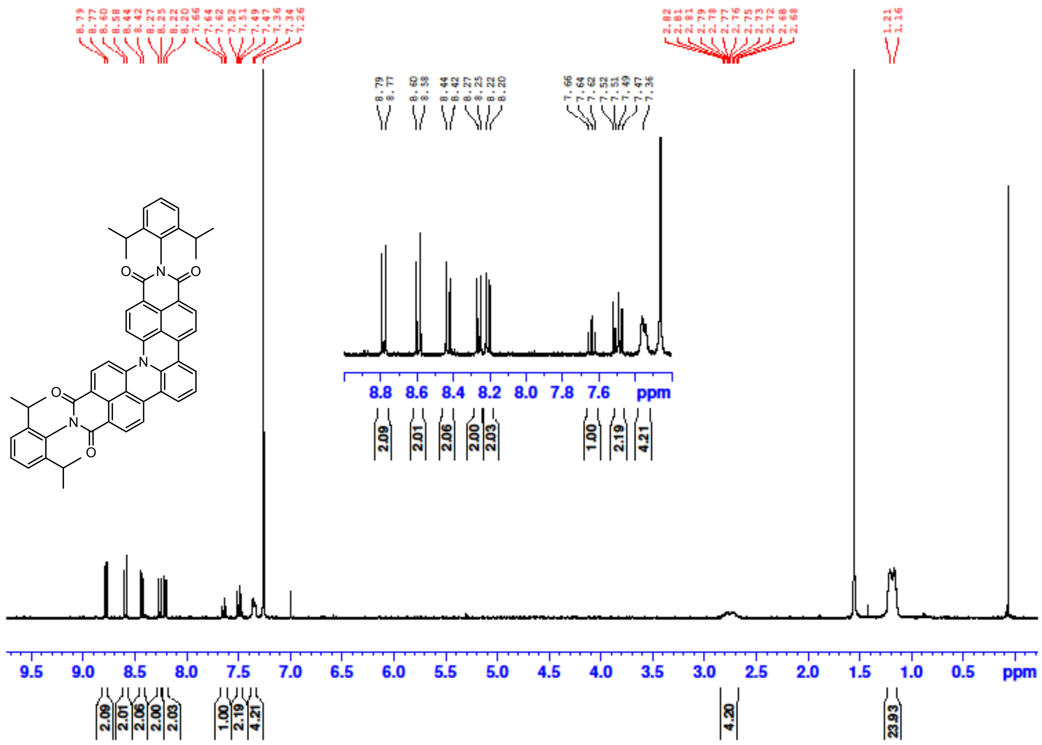

Figure S31. ^{1}H NMR (400 MHz) of **1g** in $CDCl_3$ recorded at 298 K.

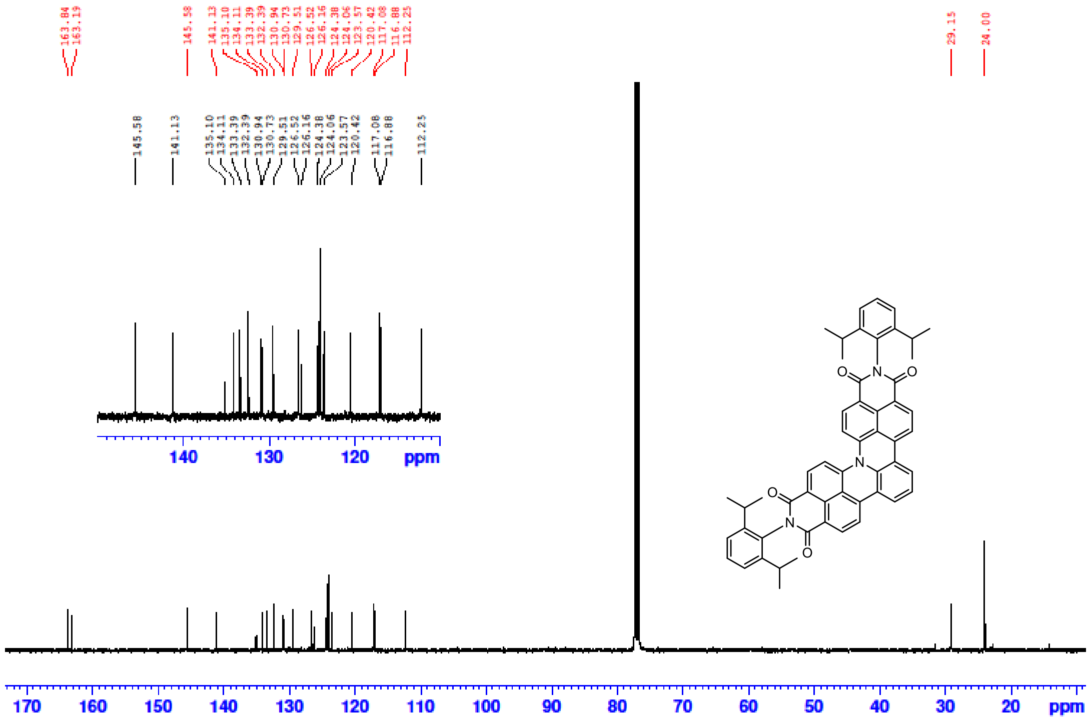

Figure S32. ^{13}C NMR (101 MHz) of **1g** in $CDCl_3$ recorded at 298 K.

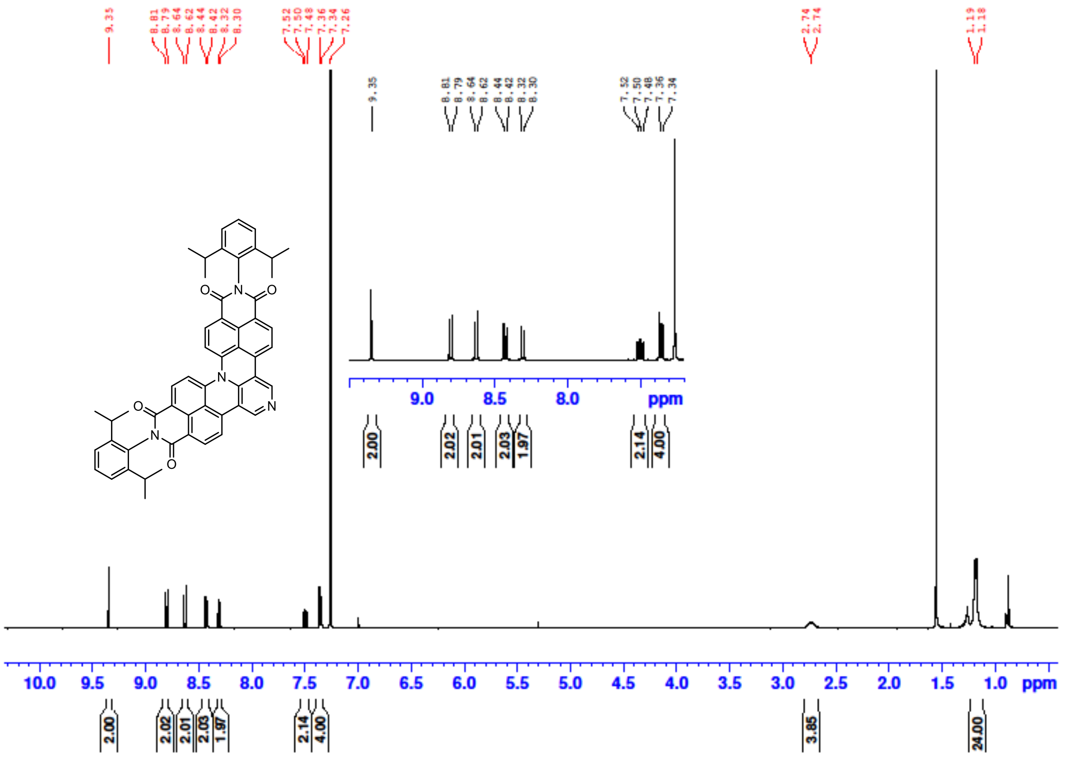

Figure S33. ^{1}H NMR (400 MHz) of **1h** in $CDCl_3$ recorded at 298 K.

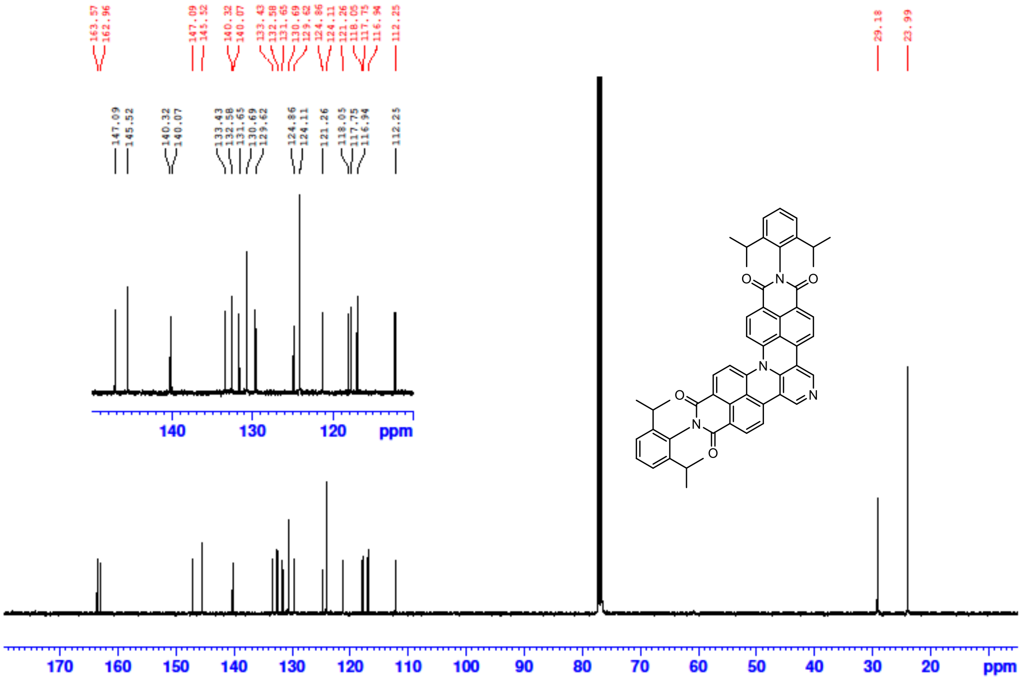

Figure S34. ^{13}C NMR (101 MHz) of **1h** in $CDCl_3$ recorded at 298 K.

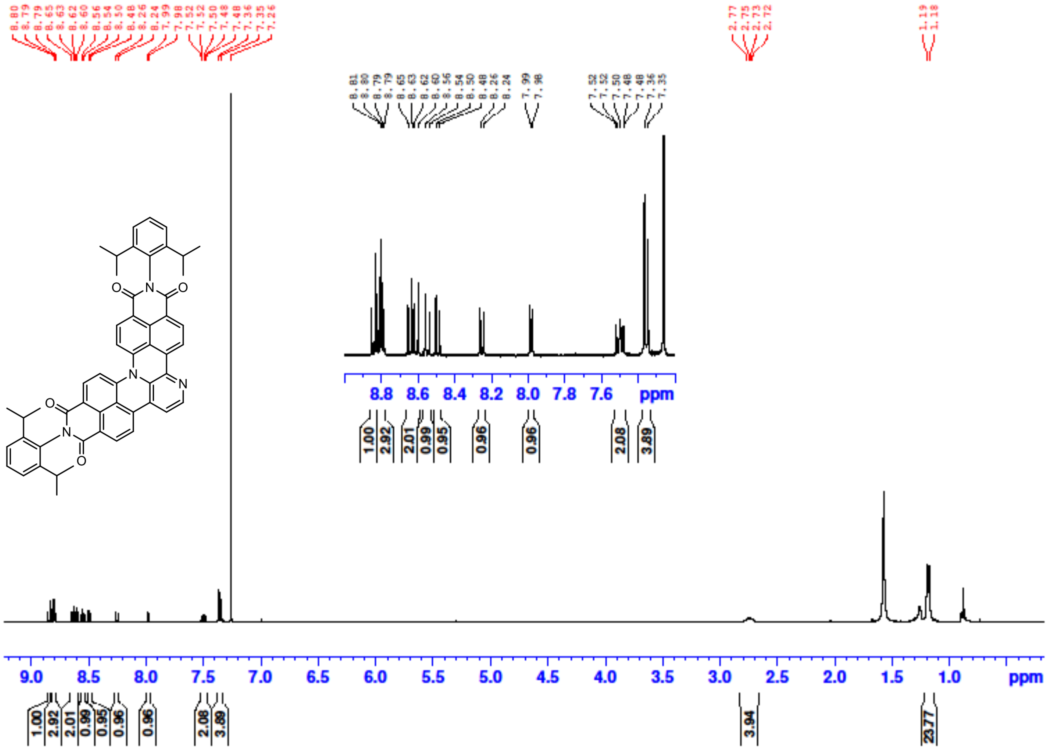

Figure S35. ^{1}H NMR (400 MHz) of **1i** in $CDCl_3$ recorded at 298 K.

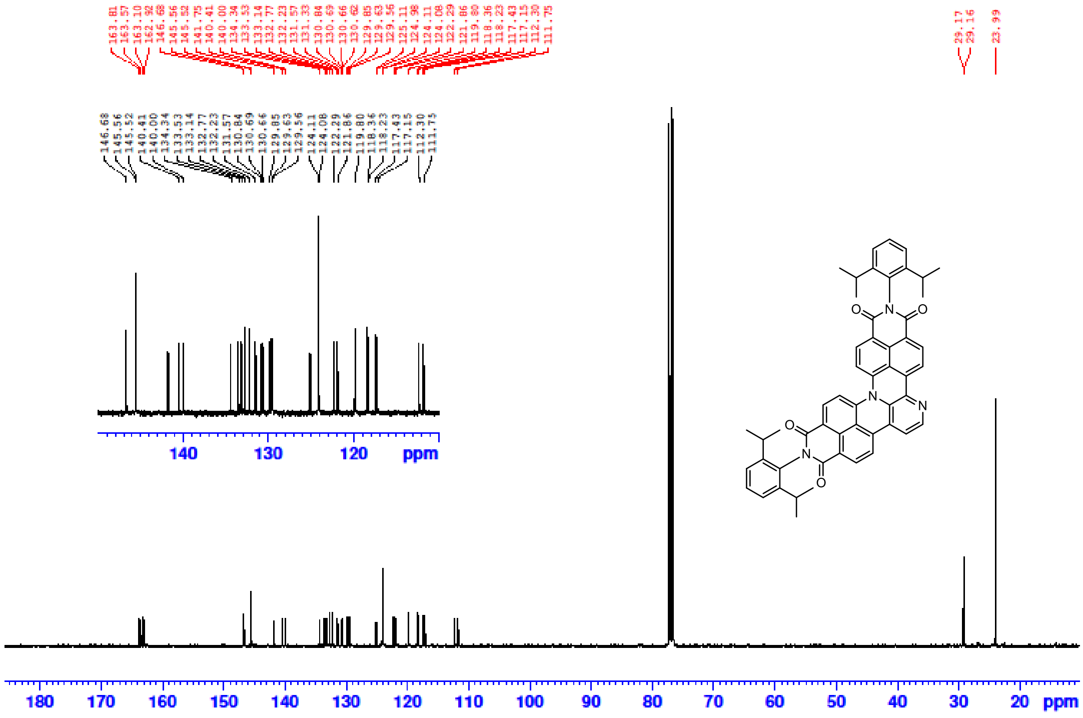

Figure S36. ^{13}C NMR (101 MHz) of **1i** in $CDCl_3$ recorded at 298 K.

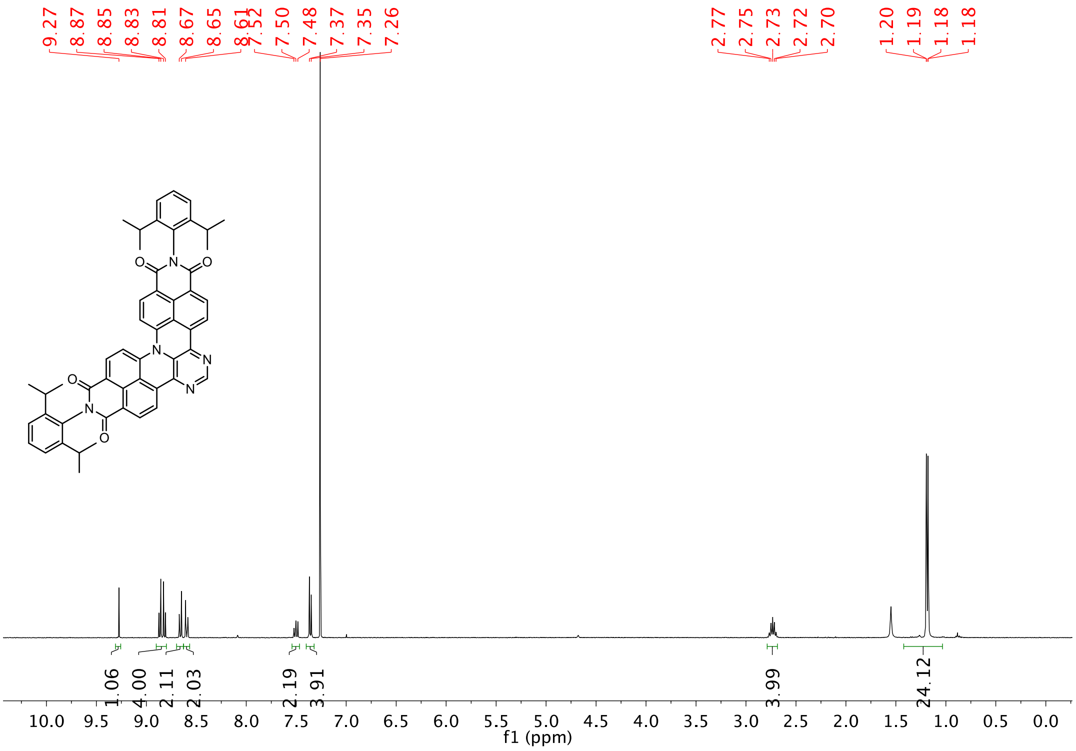

Figure S37. ^{1}H NMR (400 MHz) of **1j** in $CDCl_3$ recorded at 298 K.

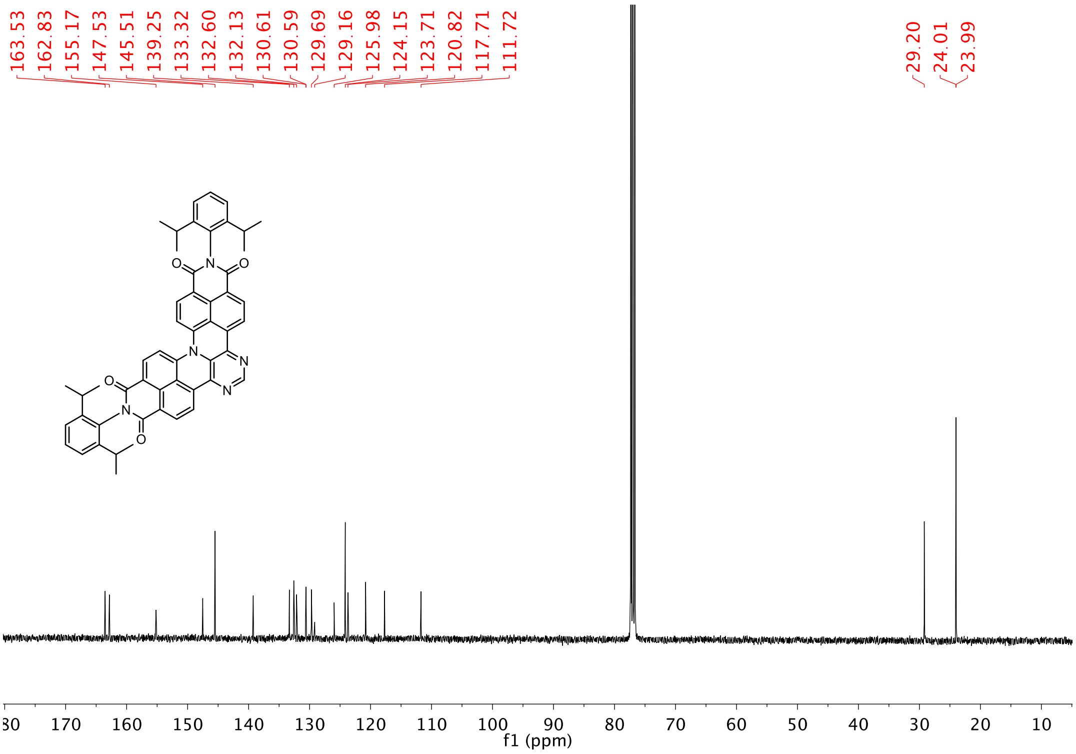

Figure S38. ^{13}C NMR (101 MHz) of **1j** in $CDCl_3$ recorded at 298 K.

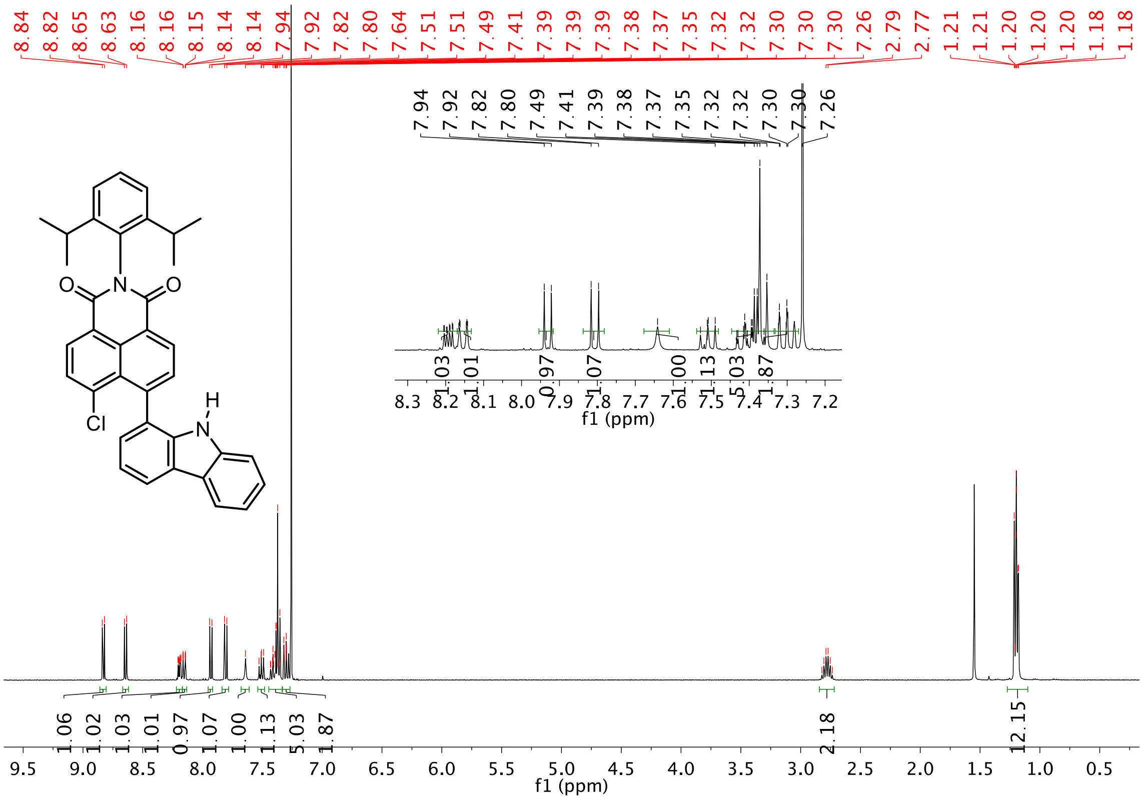

Figure S39. ^{1}H NMR (400 MHz) of **7** in $CDCl_3$ recorded at 298 K.

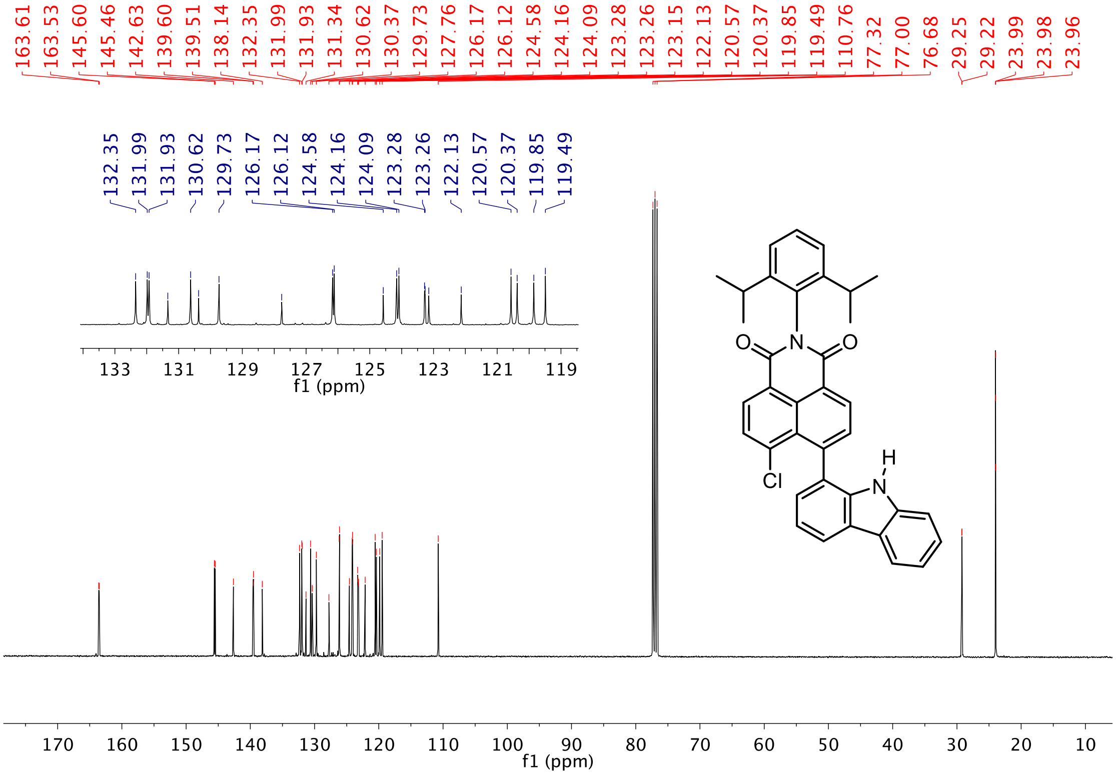

Figure S40. ^{13}C NMR (101 MHz) of **7** in $CDCl_3$ recorded at 298 K.

8. Mass spectra

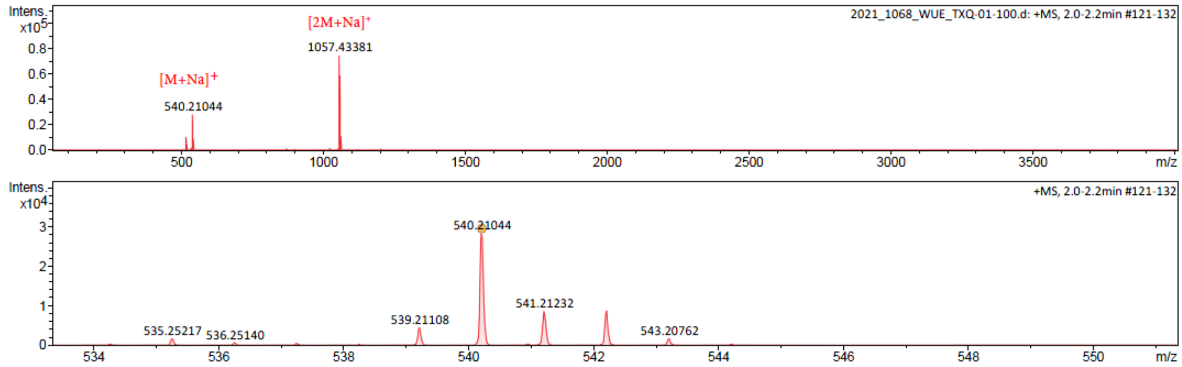

Meas. m/z	#	Ion Formula	m/z	err [ppm]	mSigma	# mSigma	Score	rdb	e⁻ Conf	N-Rule
540.21044	1	C30H33BClNNaO4	540.20834	-2.91	58.1	1	100.00	14.5	even	ok

Figure S41. HRMS spectrum (ESI-TOF) of compound **3**.

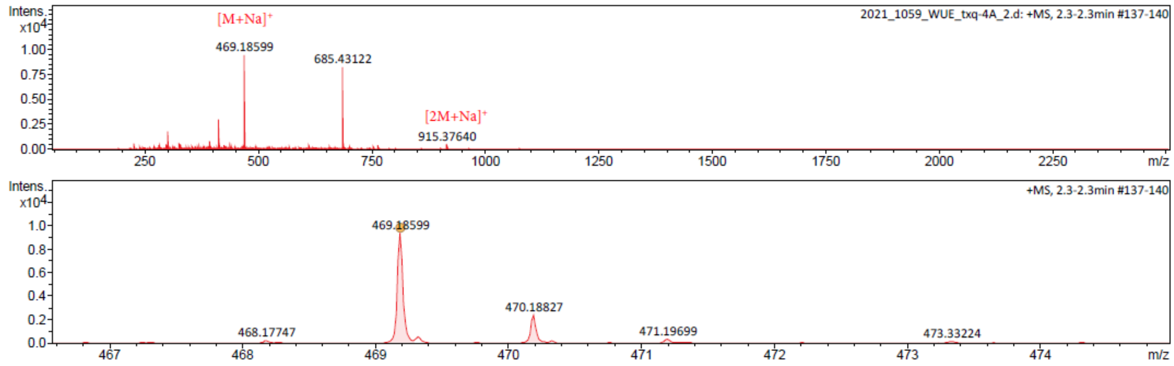

Meas. m/z	#	Ion Formula	m/z	err [ppm]	mSigma	# mSigma	Score	rdb	e⁻ Conf	N-Rule
469.18599	1	C30H26N2NaO2	469.18865	5.67	42.6	1	100.00	18.5	even	ok

Figure S42. HRMS spectrum (ESI-TOF) of compound **1a**.

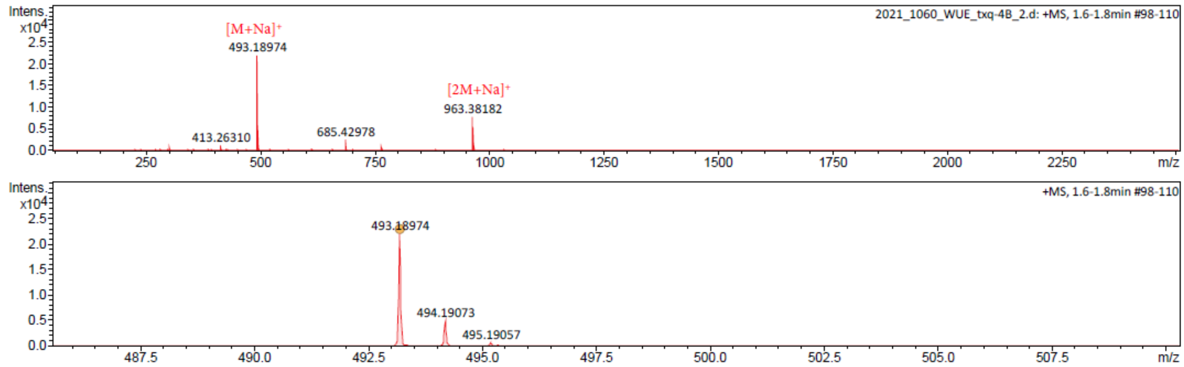

Meas. m/z	#	Ion Formula	m/z	err [ppm]	mSigma	# mSigma	Score	rdb	e⁻ Conf	N-Rule
493.18974	1	C32H26N2NaO2	493.18865	-2.21	66.9	1	100.00	20.5	even	ok

Figure S43. HRMS spectrum (ESI-TOF) of compound **1b**.

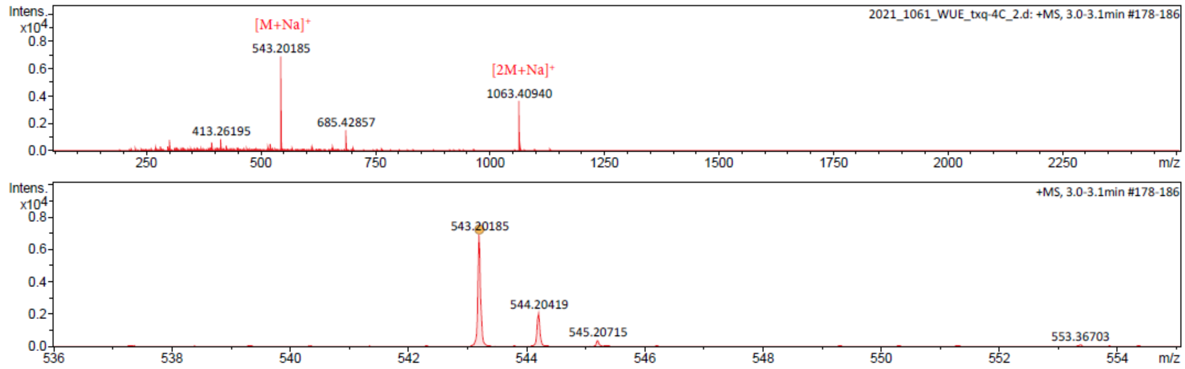

Meas. m/z	#	Ion Formula	m/z	err [ppm]	mSigma	# mSigma	Score	rdb	e⁻ Conf	N-Rule
543.20185	1	C36H28N2NaO2	543.20430	4.52	52.9	1	100.00	23.5	even	ok

Figure S44. HRMS spectrum (ESI-TOF) of compound **1c**.

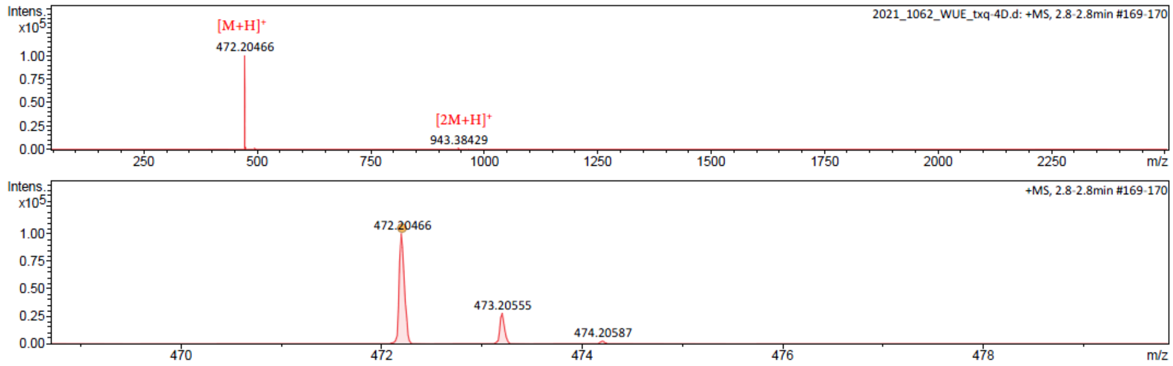

Meas. m/z	#	Ion Formula	m/z	err [ppm]	mSigma	# mSigma	Score	rdb	e⁻ Conf	N-Rule
472.20466	1	C31H26N3O2	472.20195	-5.72	40.7	1	100.00	20.5	even	ok

Figure S45. HRMS spectrum (ESI-TOF) of compound **1d**.

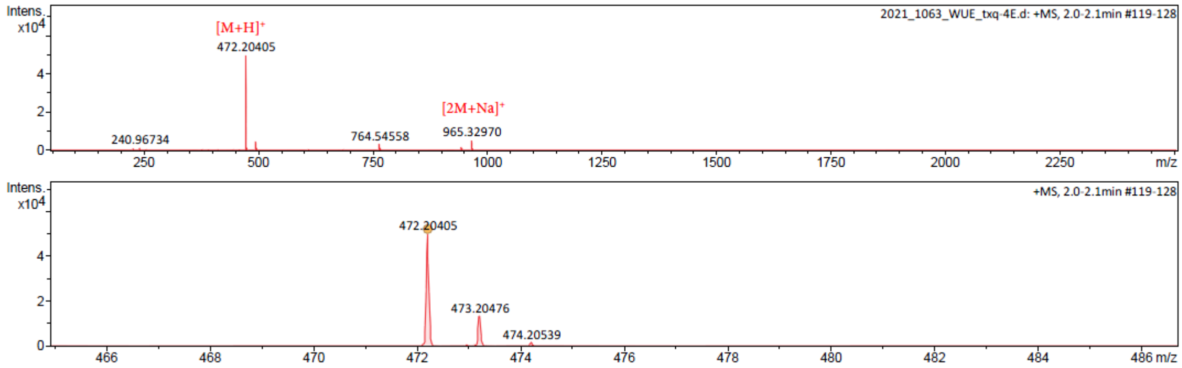

Meas. m/z	#	Ion Formula	m/z	err [ppm]	mSigma	# mSigma	Score	rdb	e⁻ Conf	N-Rule
472.20405	1	C31H26N3O2	472.20195	-4.44	44.7	1	100.00	20.5	even	ok

Figure S46. HRMS spectrum (ESI-TOF) of compound **1e**.

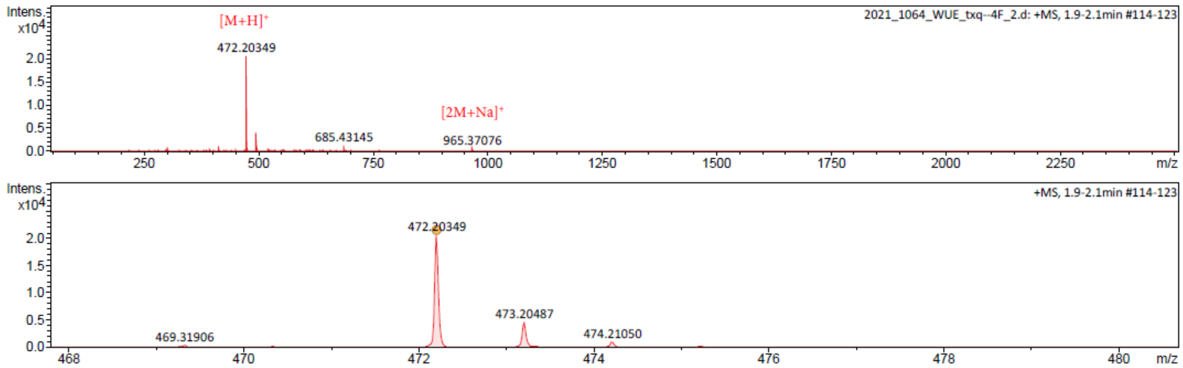

Meas. m/z	#	Ion Formula	m/z	err [ppm]	mSigma	# mSigma	Score	rdb	e¯ Conf	N-Rule
472.20349	1	C31H26N3O2	472.20195	-3.26	65.8	1	100.00	20.5	even	ok

Figure S47. HRMS spectrum (ESI-TOF) of compound **1f**.

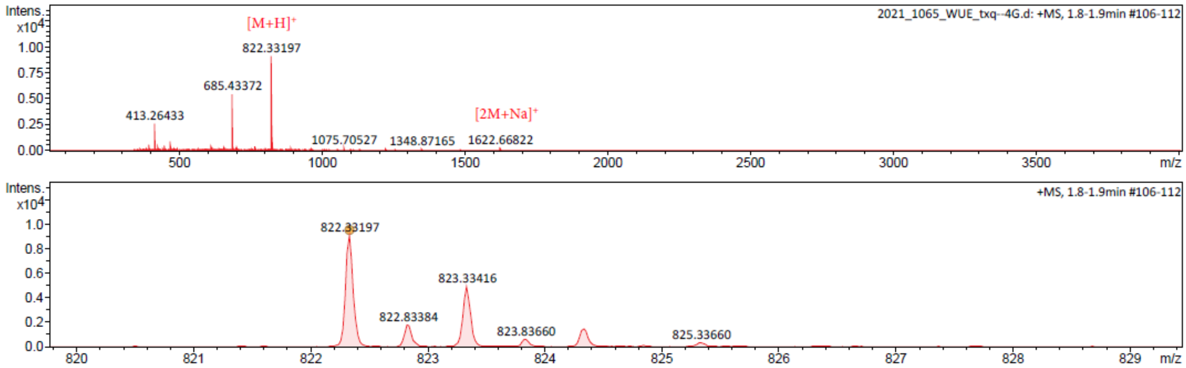

Meas. m/z	#	Ion Formula	m/z	err [ppm]	mSigma	# mSigma	Score	rdb	e¯ Conf	N-Rule
822.33197	1	C54H45N3NaO4	822.33023	-2.12	32.7	1	100.00	33.5	even	ok

Figure S48. HRMS spectrum (ESI-TOF) of compound **1g**.

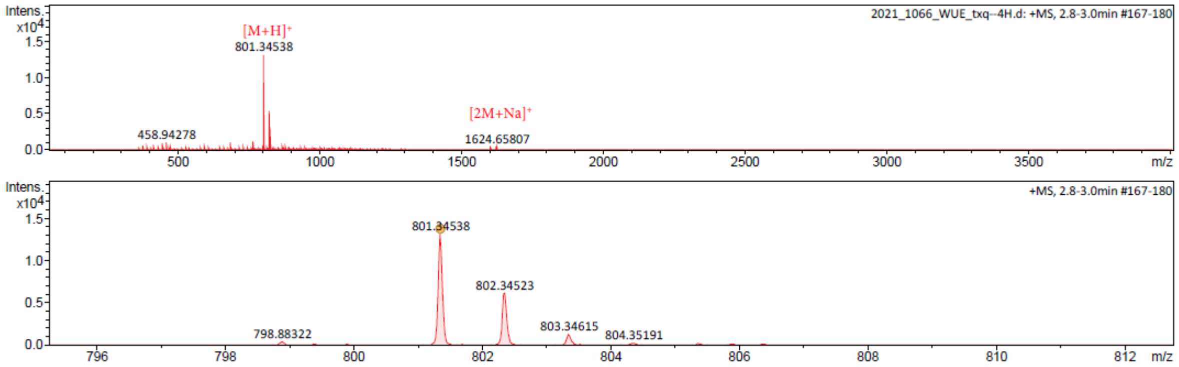

Meas. m/z	#	Ion Formula	m/z	err [ppm]	mSigma	# mSigma	Score	rdb	e¯ Conf	N-Rule
801.34538	1	C53H45N4O4	801.34353	-2.30	78.5	1	100.00	33.5	even	ok

Figure S49. HRMS spectrum (ESI-TOF) of compound **1h**.

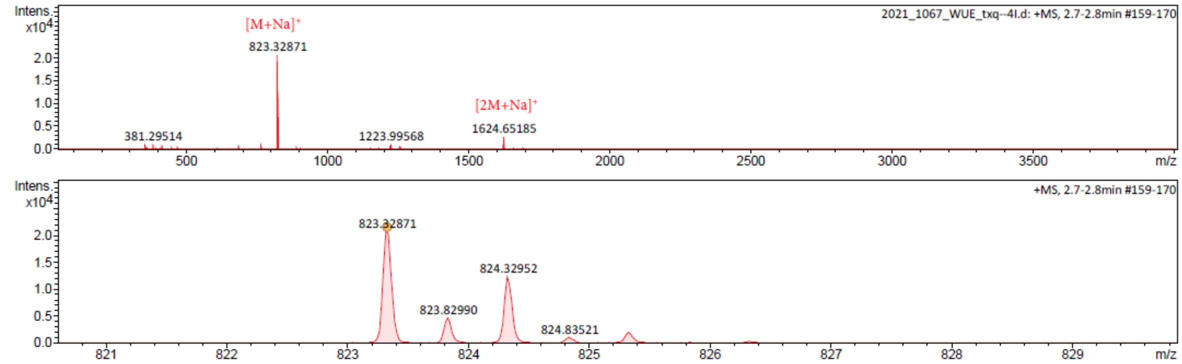

Meas. m/z	#	Ion Formula	m/z	err [ppm]	mSigma	# mSigma	Score	rdb	e⁻ Conf	N-Rule
823.32871	1	C53H44N4NaO4	823.32548	-3.93	45.8	1	100.00	33.5	even	ok

Figure S50. HRMS spectrum (ESI-TOF) of compound **1i**.

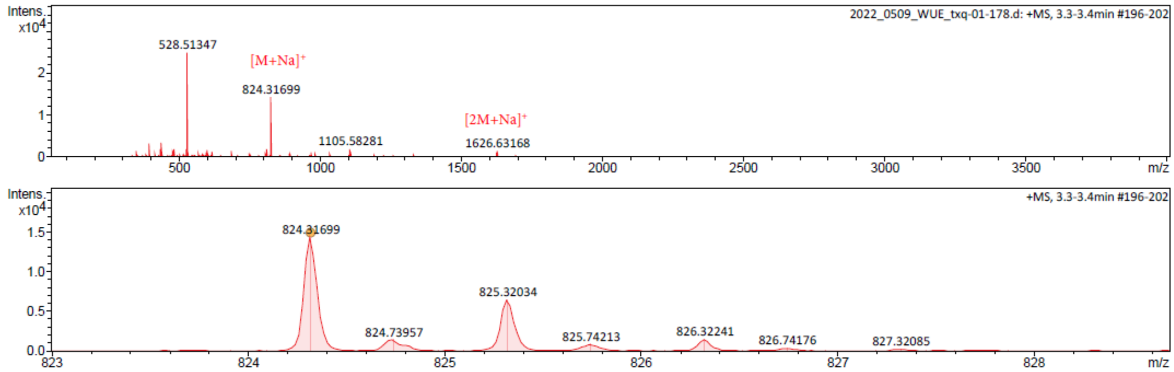

Meas. m/z	#	Ion Formula	m/z	err [ppm]	mSigma	# mSigma	Score	rdb	e⁻ Conf	N-Rule
824.31699	1	C52H43N5NaO4	824.32073	4.53	78.6	1	100.00	33.5	even	ok

Figure S51. HRMS spectrum (ESI-TOF) of compound **1j**.

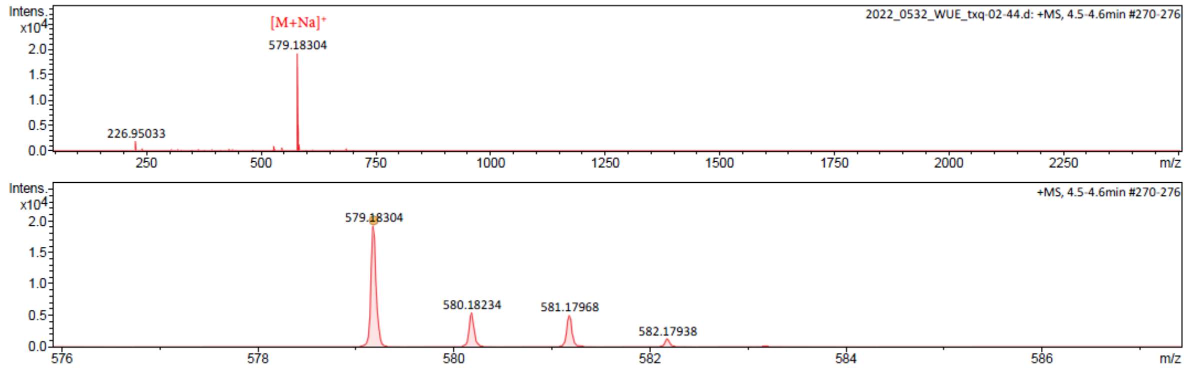

Meas. m/z	#	Ion Formula	m/z	err [ppm]	mSigma	# mSigma	Score	rdb	e⁻ Conf	N-Rule
579.18304	1	C36H29ClN2NaO2	579.18098	-3.56	87.0	1	100.00	22.5	even	ok

Figure S52. HRMS spectrum (ESI-TOF) of compound **7**.

9. Cartesian Coordinates

Table S9. Cartesian coordinates of geometry-optimized structure of **1a** (B3LYP/6-31G(d)).

Sum of electronic and thermal Free Energies = –1418.480205

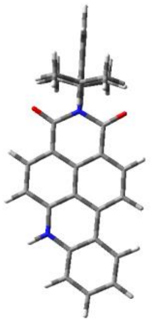

C	-6.70544000	-0.00142200	0.81036600
C	-5.31527400	-0.00103100	0.61119900
N	-4.46704300	-0.00255900	1.70598600
C	-7.56354700	0.00028100	-0.27887600
C	-7.04525900	0.00242500	-1.58066100
C	-5.67115200	0.00273200	-1.77620200
C	-4.76892200	0.00093300	-0.69575100
C	-3.31010000	0.00103400	-0.85513500
C	-2.50358200	-0.00050700	0.32428100
C	-3.09404400	-0.00235900	1.62307400
C	-1.08022000	-0.00041500	0.23029500
C	-0.28841400	-0.00187300	1.40565500
C	-0.90641100	-0.00350700	2.65204800
C	-2.29606200	-0.00377800	2.77060700
C	-2.66628800	0.00254900	-2.09667000
C	-1.27164900	0.00262600	-2.18745600
C	-0.47546600	0.00119700	-1.04940100
C	0.99601800	0.00124100	-1.18420200
N	1.73282400	-0.00015500	0.01710900
C	1.18081800	-0.00164400	1.31960500
O	1.56472600	0.00252300	-2.26884200
O	1.91217800	-0.00271300	2.30231800
C	3.18010000	0.00016800	-0.09035700
C	3.85654400	1.23143900	-0.13889000
C	5.25205900	1.20556500	-0.24267300
C	5.94666800	0.00084600	-0.29575900
C	5.25241700	-1.20421500	-0.24537500
C	3.85690700	-1.23078500	-0.14174500
C	3.12595300	2.56789400	-0.08239800
C	3.52355900	3.37235600	1.16975800
C	3.33623900	3.37803600	-1.37562900
C	3.12675400	-2.56758700	-0.08830600
C	3.33795200	-3.37512400	-1.38303800
C	3.52396800	-3.37438900	1.16244500
H	-7.09928900	-0.00306900	1.82421300
H	-4.88514900	-0.00387400	2.62679800
H	-8.63749900	-0.00005200	-0.11670500
H	-7.71415600	0.00390000	-2.43567300
H	-5.28639800	0.00444900	-2.79011200
H	-0.27664200	-0.00457400	3.53581800
H	-2.76344100	-0.00510700	3.75198200
H	-3.24492200	0.00365600	-3.01329300
H	-0.78028300	0.00376800	-3.15517400
H	5.80104900	2.14254600	-0.28211700
H	7.03075400	0.00106100	-0.37639200
H	5.80175600	-2.14089400	-0.28697000
H	2.05374300	2.36431000	-0.00382200
H	2.94706300	4.30388600	1.22560300
H	3.33288600	2.79225500	2.07866000
H	4.58683400	3.63996200	1.15549000
H	2.75701100	4.30896200	-1.34292100
H	3.01563900	2.80165200	-2.24971700
H	4.38998300	3.64722500	-1.51548600
H	2.05442200	-2.36453000	-0.00984900
H	2.75890400	-4.30623600	-1.35251700
H	3.01772200	-2.79709000	-2.25616000
H	4.39182700	-3.64382000	-1.52280600
H	2.94764300	-4.30614800	1.21623000
H	3.33278100	-2.79610100	2.07240300
H	4.58731100	-3.64173800	1.14816200

Table S10. Cartesian coordinates of geometry-optimized structure of **1b** (B3LYP/6-31G(d)).

Sum of electronic and thermal Free Energies = –1494.676374

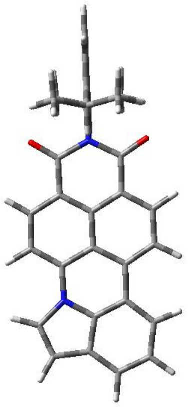

C	6.47977400	-1.22452900	1.37479400
C	5.21428900	-1.56750600	1.76004700
C	6.36636500	-0.26673200	0.29932000
C	4.98530100	-0.08314300	0.09337800
N	4.28318400	-0.87270100	0.97997600
C	7.24352800	0.46026900	-0.51714900
C	6.70316000	1.31794100	-1.48006600
C	5.31862000	1.47270600	-1.65358200
C	4.40888600	0.76178300	-0.85520400
C	2.94590500	0.79911500	-0.89676100
C	2.22977500	-0.03002500	0.03404500
C	2.88881300	-0.87841200	0.98640300
C	0.80187400	-0.02250300	0.02562900
C	0.07620400	-0.83336400	0.93567400
C	0.75688700	-1.63706000	1.83774600
C	2.15435900	-1.66493800	1.86907500
C	2.22356800	1.59256800	-1.78718100
C	0.82541200	1.59332700	-1.78802900
C	0.11278600	0.80144300	-0.89925600
C	-1.36628300	0.82986600	-0.93140900
N	-2.03159600	0.00412700	-0.00387700
C	-1.40055800	-0.83557000	0.93776800
O	-1.99811200	1.52713200	-1.71392300
O	-2.06404700	-1.52001500	1.70557400
C	-3.48326400	0.01748500	-0.01948900
C	-4.16120800	0.94592600	0.78930400
C	-5.56040500	0.93904600	0.75670900
C	-6.25632300	0.04309500	-0.04925700
C	-5.55999800	-0.86588000	-0.84014600
C	-4.16076500	-0.89860200	-0.84275200
C	-3.42954400	1.94008300	1.68338600
C	-3.71574400	3.39318400	1.25925900
C	-3.75425800	1.70266100	3.17047700
C	-3.42899100	-1.90770900	-1.71991900
C	-3.74818400	-3.35418100	-1.29666300
C	-3.72062000	-1.66979100	-3.21376700
H	7.39228500	-1.61043800	1.80801900
H	4.89001700	-2.25196400	2.52868900
H	8.32018500	0.36438900	-0.40963000
H	7.37425500	1.88588400	-2.11788000
H	4.95879700	2.15371900	-2.41824300
H	0.17743200	-2.24730100	2.52259900
H	2.66101800	-2.30269500	2.58462500
H	2.75124600	2.22331400	-2.49491100
H	0.26917700	2.21306200	-2.48368200
H	-6.11123300	1.64545800	1.37178100
H	-7.34323900	0.05316900	-0.06087200
H	-6.11046600	-1.56234000	-1.46679000
H	-2.35419800	1.77782300	1.56166400
H	-3.13644500	4.09262100	1.87427400
H	-3.44649900	3.55077200	0.20964900
H	-4.77606700	3.64574100	1.37822200
H	-3.17574200	2.38736200	3.80256500
H	-3.51149600	0.67517100	3.46090700
H	-4.81657200	1.87220500	3.38258900
H	-2.35356700	-1.76365300	-1.57793900
H	-3.16908200	-4.06569600	-1.89786000
H	-3.50207200	-3.51199100	-0.24142200
H	-4.81017700	-3.58902100	-1.43537700
H	-3.14123900	-2.36598500	-3.83236000
H	-3.45514300	-0.64744600	-3.50249400
H	-4.78119800	-1.82238100	-3.44634300

Table S11. Cartesian coordinates of geometry-optimized structure of **1c** (B3LYP/6-31G(d)).

Sum of electronic and thermal Free Energies = –1648.281459

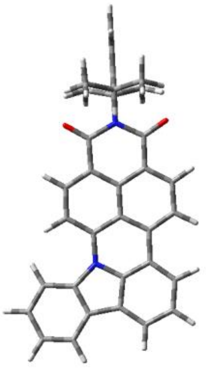

C	6.15163200	-3.37391200	0.15131000
C	7.33860300	-2.63063100	0.16960100
C	7.29349300	-1.23979500	0.12916800
C	6.05330600	-0.59961400	0.09140000
C	4.85068500	-1.35979700	0.11279600
C	4.89994800	-2.75688900	0.11797500
C	5.68403500	0.79952800	-0.01095300
C	4.27986100	0.82841300	-0.04168900
N	3.75684100	-0.45698800	0.06588700
C	6.37941500	2.00623900	-0.10175200
C	5.64776300	3.19232200	-0.22786600
C	4.24972700	3.18990500	-0.27204100
C	3.52083000	1.99081500	-0.18263900
C	2.06912800	1.83473500	-0.23051000
C	1.53812100	0.51581900	-0.04079000
C	2.37363600	-0.64156200	0.15453400
C	0.11956000	0.33743400	-0.02266200
C	-0.44027600	-0.94243600	0.21878000
C	0.39796200	-2.01651900	0.46550400
C	1.78827000	-1.87475900	0.44031500
C	1.19415000	2.90178000	-0.43402700
C	-0.18930200	2.71102400	-0.44311600
C	-0.73127800	1.45127500	-0.23489900
C	-2.20277500	1.29343400	-0.23317800
N	-2.69696000	-0.00426300	0.00081500
C	-1.90209200	-1.14536200	0.23683600
O	-2.96830600	2.22933500	-0.42382800
O	-2.42110200	-2.23463800	0.44319800
C	-4.13789400	-0.18058200	0.00487500
C	-4.78647600	-0.48454900	-1.20461700
C	-6.17615600	-0.64867100	-1.17536200
C	-6.89084200	-0.51608400	0.01133100
C	-6.22336700	-0.21533000	1.19484100
C	-4.83484700	-0.04101700	1.21756000
C	-4.03389800	-0.63531200	-2.52149000
C	-4.17925000	-2.05840200	-3.09306600
C	-4.46497400	0.43788300	-3.53928500
C	-4.13424700	0.28756300	2.53065400
C	-4.60949100	1.63848000	3.09832200
C	-4.29582000	-0.85325900	3.55319200
H	6.19734500	-4.45911800	0.15546600
H	8.29595700	-3.14224600	0.19961800
H	8.20994100	-0.65630800	0.11611300
H	4.01410200	-3.37237900	0.05961300
H	7.46508300	2.03012900	-0.07892700
H	6.17533200	4.13885200	-0.29856400
H	3.72655800	4.13484300	-0.37560700
H	-0.04962100	-2.98114500	0.68122100
H	2.39951700	-2.73040200	0.67872900
H	1.58899400	3.90021500	-0.58819900
H	-0.86715900	3.54284200	-0.60387200
H	-6.70447400	-0.88345400	-2.09544100
H	-7.96990800	-0.64744600	0.01385100
H	-6.78832100	-0.11371500	2.11749500
H	-2.96922500	-0.47844200	-2.32328300
H	-3.58488900	-2.16548600	-4.00864700
H	-3.83565000	-2.80429000	-2.36870900
H	-5.22157200	-2.28683000	-3.34523300
H	-3.87402100	0.35255000	-4.45937600
H	-4.32104900	1.44219200	-3.12744000
H	-5.52176800	0.33201600	-3.81184000
H	-3.06331700	0.38553500	2.32826200
H	-4.05385900	1.88703100	4.01075100
H	-4.45454300	2.44100700	2.36959800
H	-5.67537000	1.61514600	3.35445000
H	-3.73814400	-0.62738100	4.47028000
H	-3.92010300	-1.79637300	3.14296900
H	-5.34657200	-0.99916500	3.83053800

Table S12. Cartesian coordinates of geometry-optimized structure of **1d** (B3LYP/6-31G(d)).

Sum of electronic and thermal Free Energies = –1510.720786

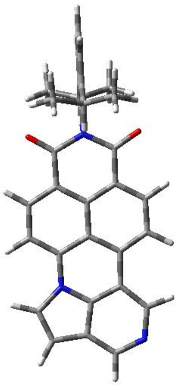

C	-6.49812800	-0.17606900	1.80477100
C	-5.23821000	-0.22729000	2.33334300
C	-6.36110900	-0.03665600	0.37307200
C	-4.97980700	-0.01190900	0.12222600
N	-4.29173100	-0.12687300	1.30399300
C	-7.18338900	0.07322600	-0.75747200
N	-6.68270700	0.19372300	-1.99311200
C	-5.34998800	0.21199700	-2.17696500
C	-4.40859600	0.11125200	-1.14038100
C	-2.94668000	0.11760800	-1.20230100
C	-2.23445200	-0.00384900	0.04188800
C	-2.89319900	-0.12786200	1.31265800
C	-0.80720700	-0.00298300	0.03212800
C	-0.08246000	-0.12172100	1.24609400
C	-0.76232200	-0.23899700	2.44767900
C	-2.16145000	-0.24276800	2.48833000
C	-2.22510300	0.23355500	-2.38809000
C	-0.82576000	0.23335300	-2.38605000
C	-0.11651800	0.11740000	-1.20000500
C	1.36410200	0.12093800	-1.24036100
N	2.02837400	0.00020800	-0.00406600
C	1.39609900	-0.12213900	1.25004100
O	1.99528900	0.22248500	-2.28310800
O	2.05591200	-0.22224500	2.27541000
C	3.48062100	0.00217600	-0.02303600
C	4.15792200	-1.22301600	-0.14946500
C	5.55712900	-1.19442700	-0.16561000
C	6.25293200	0.00613900	-0.06024800
C	5.55681500	1.20471100	0.06394500
C	4.15759800	1.22928600	0.08534400
C	3.42651600	-2.55508500	-0.26767600
C	3.71375500	-3.23486500	-1.61996600
C	3.75094200	-3.48133600	0.91975800
C	3.42595000	2.55922500	0.22436000
C	3.74530400	3.23660400	1.57067500
C	3.71814800	3.48910300	-0.96858000
H	-7.41745700	-0.23133400	2.37094800
H	-4.92647700	-0.32728800	3.36171200
H	-8.26887500	0.06504200	-0.67641900
H	-5.02658700	0.31272700	-3.21052100
H	-0.18346000	-0.32825000	3.36090700
H	-2.66944700	-0.33583500	3.44175800
H	-2.75276200	0.32574500	-3.33171500
H	-0.26771000	0.32380800	-3.31213400
H	6.10804500	-2.12598700	-0.26219300
H	7.33981400	0.00767900	-0.07494800
H	6.10743700	2.13786400	0.14578900
H	2.35096300	-2.35597900	-0.23244300
H	3.13448000	-4.16149600	-1.71339100
H	3.44567200	-2.57401900	-2.45089300
H	4.77412100	-3.49415200	-1.72260100
H	3.17233600	-4.41045000	0.84959400
H	3.50826800	-2.99351000	1.86959100
H	4.81313600	-3.75234800	0.94029800
H	2.35038100	2.35740500	0.21565800
H	3.16684400	4.16197100	1.68045800
H	3.49916400	2.57331400	2.40645100
H	4.80742400	3.49761000	1.64752300
H	3.13913500	4.41652300	-0.88167400
H	3.45288200	3.00311400	-1.91328800
H	4.77879200	3.76291800	-1.01521500

Table S13. Cartesian coordinates of geometry-optimized structure of **1e** (B3LYP/6-31G(d)).

Sum of electronic and thermal Free Energies = –1510.723858

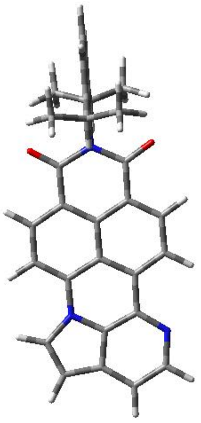

C	6.50869100	-0.01771100	1.80224400
C	5.24813400	-0.02276000	2.34157600
C	6.36475500	-0.00375700	0.36655600
C	4.98067800	-0.00116300	0.12739800
N	4.29573700	-0.01269200	1.32130200
C	7.17257000	0.00711900	-0.78056700
C	6.52911100	0.01927700	-2.02429500
N	5.19579600	0.02157600	-2.22223600
C	4.40820500	0.01128300	-1.13792800
C	2.94486200	0.01222100	-1.19221100
C	2.23732300	-0.00003400	0.05714500
C	2.89616700	-0.01269500	1.33378500
C	0.81117600	0.00020600	0.04231500
C	0.08417800	-0.01201000	1.26053800
C	0.76221500	-0.02397100	2.46912700
C	2.16188100	-0.02435200	2.51333700
C	2.23913500	0.02429300	-2.38957200
C	0.83885600	0.02461200	-2.39132400
C	0.12670900	0.01276100	-1.20002400
C	-1.35445500	0.01320400	-1.24305000
N	-2.02213400	0.00021900	-0.00184300
C	-1.39430100	-0.01218500	1.26031500
O	-1.98446200	0.02433700	-2.29115800
O	-2.05756800	-0.02240700	2.28842900
C	-3.47424100	-0.00006500	-0.02541000
C	-4.15198200	1.23140000	-0.02455500
C	-5.55113200	1.20438100	-0.04768300
C	-6.24658300	-0.00076900	-0.07086900
C	-5.55011300	-1.20556600	-0.07128200
C	-4.15095100	-1.23188800	-0.04866500
C	-3.42091700	2.56862200	0.00109400
C	-3.70792200	3.39048500	-1.26991100
C	-3.74584100	3.36101900	1.28163200
C	-3.41881300	-2.56876300	-0.04954800
C	-3.74233500	-3.38638700	1.21546000
C	-3.70600000	-3.36571000	-1.33619600
H	7.42999600	-0.02337800	2.36787900
H	4.94745300	-0.03282800	3.37846200
H	8.25688100	0.00631000	-0.72772500
H	7.13000200	0.02784400	-2.93103900
H	0.18174900	-0.03307200	3.38563400
H	2.66742000	-0.03386200	3.47284300
H	2.79869300	0.03341100	-3.31864900
H	0.28187300	0.03402600	-3.32244000
H	-6.10223100	2.14084100	-0.04768500
H	-7.33343300	-0.00105600	-0.08877800
H	-6.10045400	-2.14229700	-0.08952800
H	-2.34535400	2.36711800	0.01470400
H	-3.12905900	4.32202700	-1.26213400
H	-3.43874100	2.82363200	-2.16723200
H	-4.76836000	3.65889500	-1.34471600
H	-3.16746200	4.29241600	1.31256400
H	-3.50337000	2.77322300	2.17313600
H	-4.80816800	3.62784300	1.33097800
H	-2.34339200	-2.36677800	-0.03261300
H	-3.16365400	-4.31804000	1.22750800
H	-3.49926600	-2.81626100	2.11819000
H	-4.80455700	-3.65444200	1.26041300
H	-3.12614100	-4.29659900	-1.34724700
H	-3.43812400	-2.78094400	-2.22237300
H	-4.76619600	-3.63377600	-1.41548800

Table S14. Cartesian coordinates of geometry-optimized structure of **1f** (B3LYP/6-31G(d)).

Sum of electronic and thermal Free Energies = –1510.720309

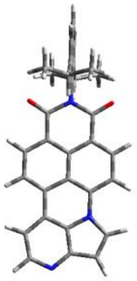

C	-6.50372000	0.27554700	1.77131400
C	-5.24545400	0.35823400	2.30228500
C	-6.35615900	0.05444600	0.35047200
C	-4.97078600	0.01705800	0.10958300
N	-4.28968300	0.20052400	1.28831700
N	-7.25067900	-0.09953500	-0.63857400
C	-6.72402600	-0.28920300	-1.85746300
C	-5.35094600	-0.33712600	-2.16580400
C	-4.40501900	-0.17779200	-1.14234200
C	-2.94060500	-0.18649100	-1.19830300
C	-2.23285800	0.00602400	0.03885200
C	-2.89456000	0.20244400	1.30089100
C	-0.80593700	0.00463400	0.03002600
C	-0.08104000	0.19199300	1.23478200
C	-0.76232900	0.37767700	2.42759400
C	-2.16091800	0.38406300	2.46775600
C	-2.22060000	-0.36956200	-2.37474700
C	-0.82035100	-0.36911400	-2.37255300
C	-0.11467900	-0.18569000	-1.19383200
C	1.36768700	-0.19154100	-1.23278000
N	2.03061500	-0.00122400	-0.00626800
C	1.39683600	0.19254000	1.23924900
O	1.99729900	-0.35223200	-2.26889100
O	2.05718300	0.35065300	2.25668300
C	3.48299200	-0.00378300	-0.02384400
C	4.16012300	-1.22257800	0.15469100
C	5.55935500	-1.19860500	0.13332600
C	6.25515500	-0.00871900	-0.05808600
C	5.55910800	1.18369100	-0.23203500
C	4.15985000	1.21266600	-0.21880300
C	3.42878200	-2.54283000	0.36761400
C	3.72076300	-3.53740800	-0.77203600
C	3.74857000	-3.14498600	1.74917500
C	3.42826800	2.53597800	-0.41092100
C	3.74640500	3.52340100	0.72805000
C	3.72158700	3.14489500	-1.79530700
H	-7.43149800	0.36070200	2.31883300
H	-4.93962200	0.51742600	3.32511400
H	-7.44398800	-0.41470300	-2.66362400
H	-5.04929000	-0.49742100	-3.19570300
H	-0.18400900	0.51880900	3.33461600
H	-2.66937800	0.53140400	3.41414600
H	-2.75003100	-0.51525800	-3.31087200
H	-0.26052400	-0.51193800	-3.29086400
H	6.11023700	-2.12533400	0.26880200
H	7.34204100	-0.01068800	-0.07154800
H	6.10978500	2.10852500	-0.38064300
H	2.35313600	-2.34202800	0.34827800
H	3.14165300	-4.45849300	-0.63372700
H	3.45563300	-3.10463600	-1.74234000
H	4.78135700	-3.81350900	-0.80339100
H	3.17030600	-4.06300700	1.90968400
H	3.50239800	-2.43698400	2.54740100
H	4.81073600	-3.40117400	1.84006100
H	2.35264900	2.33820800	-0.37059300
H	3.16789100	4.44722200	0.60637600
H	3.49924300	3.08591700	1.70095200
H	4.80844700	3.79549700	0.73948100
H	3.14227600	4.06501300	-1.93904400
H	3.45774800	2.44206200	-2.59248500
H	4.78220600	3.39947300	-1.90639800

Table S15. Cartesian coordinates of geometry-optimized structure of *P*-**1g** (B3LYP/6-31G(d)).

Sum of electronic and thermal Free Energies = –2549.415738

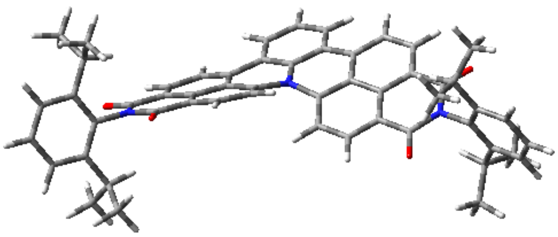

O	5.00569900	-2.42089400	-0.83606200
O	7.18714300	1.21739400	0.90046300
O	-7.18709700	1.21735700	-0.90089400
O	-5.00574900	-2.42088800	0.83585600
N	-0.00000200	1.57129200	-0.00013000
N	6.09883300	-0.60439700	0.03551800
N	-6.09889100	-0.60431300	-0.03554900
C	1.28144300	-0.40357100	-0.73418200
H	0.37997000	-0.89315500	-1.07863600
C	2.50125000	-1.08456600	-0.81885800
H	2.53907000	-2.08909100	-1.22730800
C	3.67949800	-0.50648200	-0.37189900
C	3.65936400	0.82655100	0.10059800
C	2.43336500	1.55504700	0.13688900
C	4.85347000	1.45626900	0.52795300
C	4.82192500	2.77356200	0.95971500
H	5.74747300	3.22704100	1.29896400
C	3.63247400	3.51180400	0.92553800
H	3.65441000	4.54929400	1.23972100
C	2.44124000	2.93493200	0.48525900
C	1.20280800	3.68352500	0.25935400
C	-0.00000900	2.98171900	-0.00004400
C	1.17683000	5.08566300	0.24184300
H	2.09607900	5.63613100	0.40635900
C	-0.00005800	5.78325700	0.00013700
H	-0.00005900	6.86875000	0.00020400
C	-1.17691700	5.08566500	-0.24165800
H	-2.09621000	5.63609300	-0.40607800
C	-1.20283200	3.68352200	-0.25935700
C	-2.44125000	2.93493300	-0.48534300
C	-2.43336900	1.55503600	-0.13703700
C	-3.63246000	3.51184400	-0.92563000
H	-3.65434600	4.54934700	-1.23978200
C	-4.82191800	2.77361700	-0.95985700
H	-5.74745000	3.22709800	-1.29914500
C	-4.85347300	1.45631600	-0.52811600
C	-3.65938100	0.82656400	-0.10077200
C	-3.67954000	-0.50649600	0.37166000
C	-2.50127400	-1.08463400	0.81848000
H	-2.53906500	-2.08919200	1.22685200
C	-1.28144200	-0.40367400	0.73377900
H	-0.37999000	-0.89338200	1.07810300
C	-1.22152200	0.88538000	0.20268100
C	1.22154400	0.88542700	-0.20292300
C	4.94273700	-1.27162400	-0.42271500
C	6.13811500	0.71937200	0.51669900
C	-6.13811000	0.71940500	-0.51696900
C	-4.94279600	-1.27160100	0.42251400
C	7.35038500	-1.34102000	0.00525400
C	8.14402600	-1.28028100	-1.15339300
C	9.34513600	-1.99874000	-1.15774100
H	9.97921600	-1.97128000	-2.03966500
C	9.73940700	-2.74674600	-0.05247700
H	10.67597800	-3.29792900	-0.07510000
C	8.93413900	-2.78913200	1.08166600
H	9.24911400	-3.37550000	1.94047500
C	7.72364300	-2.08866100	1.13562000
C	7.74116900	-0.47210000	-2.38137400
H	6.78309400	0.01256000	-2.16946200
C	7.52335100	-1.38244400	-3.60500000
H	6.77377800	-2.15052900	-3.38800400
H	8.45041200	-1.88802500	-3.90001500
H	7.17805800	-0.79371800	-4.46356000
C	8.75637900	0.64884100	-2.67481600
H	8.42106000	1.25580200	-3.52456700
H	9.74324900	0.24262300	-2.92599700
H	8.87271600	1.30487300	-1.80576000
C	6.86561700	-2.15617400	2.39363000
H	5.97032800	-1.54924700	2.22671600
C	6.38824700	-3.59418700	2.67201900
H	7.23051000	-4.26499900	2.87845600
H	5.84053400	-3.99490100	1.81262300
H	5.72537000	-3.61498400	3.54555100
C	7.59909900	-1.55140700	3.60597000
H	7.89983600	-0.51858000	3.40153800
H	8.50056300	-2.12296100	3.85650800
H	6.94761000	-1.55466300	4.48829300
C	-7.35050000	-1.34083000	-0.00521200
C	-7.72390500	-2.08845200	-1.13554200

C	-8.93450000	-2.78874400	-1.08152900
H	-9.24957900	-3.37510200	-1.94030700
C	-9.73973200	-2.74619700	0.05263500
H	-10.67638600	-3.29723800	0.07530300
C	-9.34532300	-1.99820500	1.15785800
H	-9.97936900	-1.97064100	2.03980400
C	-8.14411000	-1.27991800	1.15345000
C	-6.86582800	-2.15629200	-2.39349900
H	-5.97078000	-1.54892500	-2.22689200
C	-7.59941900	-1.55252800	-3.60625700
H	-7.90056500	-0.51969400	-3.40246300
H	-8.50062700	-2.12456400	-3.85660500
H	-6.94781900	-1.55604400	-4.48849600
C	-6.38791000	-3.59431600	-2.67095400
H	-5.72500700	-3.61545300	-3.54446000
H	-7.22995200	-4.26554400	-2.87695000
H	-5.84008600	-3.99425800	-1.81127200
C	-7.74099500	-0.47192700	2.38147400
H	-6.78334900	0.01339800	2.16914000
C	-8.75659600	0.64830900	2.67615000
H	-8.87395300	1.30468600	1.80748600
H	-8.42093200	1.25508200	3.52589900
H	-9.74307700	0.24152800	2.92794500
C	-7.52188800	-1.38276600	3.60452600
H	-6.77203400	-2.15032500	3.38666700
H	-8.44852200	-1.88900300	3.89976200
H	-7.17642600	-0.79429900	4.46319800

Table S16. Cartesian coordinates of geometry-optimized structure of *M*-**1g** (B3LYP/6-31G(d)).

Sum of electronic and thermal Free Energies = –2549.415733

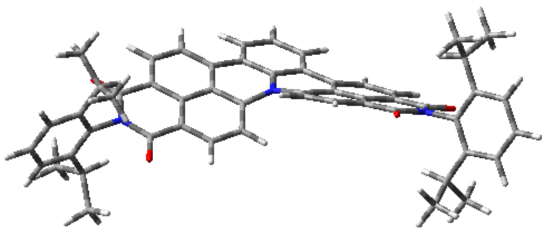

O	5.00568000	-2.42064600	0.83670500
O	7.18704200	1.21715300	-0.90097600
O	-7.18703100	1.21716800	0.90090900
O	-5.00566300	-2.42068800	-0.83664400
N	0.00000500	1.57136300	-0.00004000
N	6.09876200	-0.60442000	-0.03551400
N	-6.09874800	-0.60443500	0.03551600
C	1.28136900	-0.40350000	0.73406200
H	0.37985500	-0.89302900	1.07849600
C	2.50117100	-1.08445300	0.81890600
H	2.53899600	-2.08892900	1.22747700
C	3.67944500	-0.50640900	0.37192300
C	3.65933600	0.82656400	-0.10071800
C	2.43334400	1.55506500	-0.13707400
C	4.85343200	1.45621800	-0.52820000
C	4.82189900	2.77348200	-0.96003500
H	5.74743200	3.22691400	-1.29939100
C	3.63247000	3.51177100	-0.92581200
H	3.65444000	4.54925100	-1.24001700
C	2.44123800	2.93495900	-0.48545700
C	1.20281600	3.68357500	-0.25951400
C	0.00000300	2.98177800	-0.00006300
C	1.17686000	5.08571700	-0.24190900
H	2.09610800	5.63618000	-0.40644400
C	-0.00000100	5.78331100	-0.00010600
H	-0.00000200	6.86880400	-0.00012200
C	-1.17686000	5.08572100	0.24171900
H	-2.09610900	5.63618700	0.40623700
C	-1.20281200	3.68358000	0.25936600
C	-2.44123200	2.93496700	0.48533200
C	-2.43333400	1.55506300	0.13699300
C	-3.63246500	3.51179000	0.92567000
H	-3.65443700	4.54928000	1.23984400
C	-4.82189200	2.77349900	0.95991700
H	-5.74742700	3.22693900	1.29925800
C	-4.85342200	1.45622200	0.52812300
C	-3.65932400	0.82655700	0.10066100
C	-3.67942900	-0.50643100	-0.37193400
C	-2.50115300	-1.08448700	-0.81889900
H	-2.53897500	-2.08897700	-1.22743500
C	-1.28135400	-0.40352700	-0.73407900
H	-0.37983900	-0.89306600	-1.07849700
C	-1.22151300	0.88544900	-0.20277500
C	1.22152500	0.88545800	0.20271500
C	4.94268900	-1.27152700	0.42292800
C	6.13802500	0.71923100	-0.51707700
C	-6.13801300	0.71922900	0.51702600
C	-4.94266900	-1.27155700	-0.42290900
C	7.35033300	-1.34100500	-0.00510600
C	7.72381400	-2.08851600	-1.13548500
C	8.93437100	-2.78887500	-1.08143000
H	9.24953900	-3.37510100	-1.94026500
C	9.73946800	-2.74653400	0.05283600
H	10.67608600	-3.29763500	0.07554200
C	9.34497200	-1.99866800	1.15811400
H	9.97890600	-1.97127400	2.04014600
C	8.14381600	-1.28029000	1.15365400
C	6.86601500	-2.15592700	-2.39365600
H	5.97037800	-1.54955000	-2.22660800
C	6.38941800	-3.59404300	-2.67282000
H	5.84177800	-3.99548300	-1.81371300
H	7.23202900	-4.26434400	-2.87948900
H	5.72666900	-3.61472900	-3.54645000
C	7.59941700	-1.55022300	-3.60558100
H	6.94811600	-1.55343600	-4.48804500
H	8.50123400	-2.12120200	-3.85616400
H	7.89956100	-0.51733600	-3.40060700
C	7.74060600	-0.47243700	2.38173600
H	6.78312100	0.01315000	2.16927800
C	7.52102900	-1.38347500	3.60456000
H	8.44749400	-1.88998000	3.89987300
H	6.77104500	-2.15080800	3.38637200
H	7.17552900	-0.79510300	4.46328200
C	8.75637700	0.64752300	2.67687200
H	8.87409800	1.30404600	1.80836500
H	9.74270900	0.24049600	2.92884700
H	8.42062400	1.25420900	3.52664700
C	-7.35030900	-1.34103700	0.00514400
C	-8.14375700	-1.28045600	-1.15364700
C	-9.34489600	-1.99886400	-1.15807400

H	-9.97880300	-1.97157000	-2.04012700
C	-9.73941100	-2.74662500	-0.05273200
H	-10.67601600	-3.29774900	-0.07541100
C	-8.93435000	-2.78883200	1.08156400
H	-9.24953700	-3.37496900	1.94045300
C	-7.72380600	-2.08844800	1.13558500
C	-7.74055700	-0.47267100	-2.38177800
H	-6.78301400	0.01283600	-2.16939700
C	-8.75626400	0.64737000	-2.67683700
H	-8.87385700	1.30390900	-1.80832500
H	-9.74264900	0.24040900	-2.92871300
H	-8.42054400	1.25402200	-3.52665000
C	-7.52114400	-1.38372700	-3.60461400
H	-7.17564800	-0.79538100	-4.46335500
H	-8.44766800	-1.89015200	-3.89987700
H	-6.77120900	-2.15112400	-3.38647900
C	-6.86606900	-2.15566900	2.39380700
H	-5.97033100	-1.54947300	2.22663700
C	-7.59945500	-1.54951900	3.60552400
H	-7.89942900	-0.51664400	3.40024400
H	-6.94821600	-1.55259300	4.48803500
H	-8.50137800	-2.12029700	3.85618800
C	-6.38969400	-3.59377100	2.67340400
H	-5.84207800	-3.99554700	1.81443800
H	-7.23239900	-4.26389900	2.88025000
H	-5.72697500	-3.61429300	3.54706100

Table S17. Cartesian coordinates of geometry-optimized structure of the transition state of the racemization of **1g** (B3LYP/6-31G(d)).

Sum of electronic and thermal Free Energies = –2549.391244

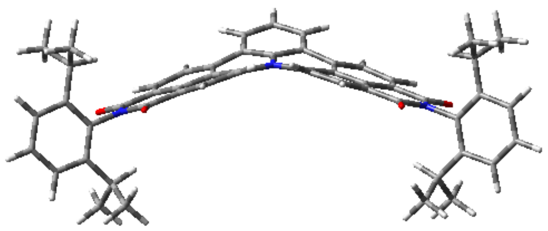

O	-5.17613200	-2.13534600	1.48117300
O	-7.06587900	0.95978300	-1.31730400
O	7.07456000	0.97910100	-1.28960100
O	5.16513700	-2.15620000	1.45020700
N	0.00040800	1.67554000	0.40336400
N	-6.12485800	-0.60650800	0.06189100
N	6.12456200	-0.60505900	0.06256900
C	-1.49799300	-0.03957600	1.49914400
H	-0.76193600	-0.42104900	2.16670100
C	-2.72049400	-0.72378600	1.54454900
H	-2.82254500	-1.58837500	2.19211700
C	-3.79785100	-0.33361900	0.78222100
C	-3.68162500	0.85296100	0.02399600
C	-2.44650000	1.57863600	-0.00569000
C	-4.81080600	1.33949200	-0.67868100
C	-4.73932800	2.54392100	-1.36014100
H	-5.61448500	2.88862900	-1.90053700
C	-3.56636800	3.29767100	-1.32720900
H	-3.53352100	4.23929400	-1.86232200
C	-2.43690400	2.84404100	-0.64231900
C	-1.20955200	3.61650400	-0.55595300
C	0.00025100	2.96085500	-0.21400400
C	-1.19244200	4.98052700	-0.88492900
H	-2.13448300	5.49072700	-1.04821000
C	-0.00281000	5.68519300	-0.98786000
H	-0.00459300	6.74952900	-1.20007300
C	1.18938900	4.98715800	-0.87145000
H	2.13040200	5.50222100	-1.02521500
C	1.21000300	3.62298400	-0.54332300
C	2.44118500	2.85586900	-0.62035300
C	2.44853700	1.58448500	0.00526500
C	3.57561400	3.31781700	-1.29136600
H	3.54580400	4.26479500	-1.81712200
C	4.74899400	2.56512300	-1.32459500
H	5.62769300	2.91671800	-1.85474200
C	4.81620400	1.35188300	-0.65859700
C	3.68309600	0.85707800	0.03196400
C	3.79503300	-0.33893000	0.77619300
C	2.71752700	-0.73096200	1.53741000
H	2.81817100	-1.60024600	2.17894800
C	1.49755900	-0.04237300	1.50052500
H	0.77626100	-0.39609500	2.20051400
C	1.27702800	1.05888600	0.66456300
C	-1.27647000	1.05423000	0.65621700
C	-5.05514900	-1.11050500	0.82490600
C	-6.08545100	0.58343300	-0.69055400
C	6.09040700	0.59528900	-0.67331800
C	5.04978400	-1.11982300	0.81125600
C	-7.36707000	-1.35911000	0.06506600
C	-8.32825900	-1.07652800	1.05106900
C	-9.51594800	-1.81664800	1.03243900
H	-10.27721400	-1.62141300	1.78273200
C	-9.73681800	-2.79743200	0.07018400
H	-10.66593500	-3.36153200	0.07230900
C	-8.76795300	-3.05602000	-0.89465600
H	-8.94820300	-3.82350100	-1.64234600
C	-7.56317400	-2.34397100	-0.91862700
C	-8.11700000	-0.01095200	2.12026000
H	-7.14133500	0.45409400	1.94819200
C	-8.07688600	-0.63234000	3.52951400
H	-7.29823300	-1.39950000	3.59254000
H	-9.03408900	-1.09936400	3.78990200
H	-7.86706200	0.13777200	4.28176700
C	-9.17133100	1.10740800	2.01711100
H	-8.96857500	1.89318100	2.75489600
H	-10.18194900	0.72710900	2.20680300
H	-9.16210300	1.55901400	1.01955900
C	-6.52253600	-2.65250200	-1.98892100
H	-5.66451100	-1.99229000	-1.82848100
C	-6.00551300	-4.09887100	-1.86965300
H	-6.80566200	-4.82759900	-2.04496700
H	-5.59342800	-4.28385100	-0.87210300
H	-5.21788900	-4.28578600	-2.60958700
C	-7.06274800	-2.35293000	-3.40028900
H	-7.39409000	-1.31206500	-3.47637100
H	-7.91334000	-2.99791100	-3.65034400
H	-6.28328200	-2.52439000	-4.15253600
C	7.36614700	-1.35861200	0.06201900
C	8.32230100	-1.09040800	1.05699500

C	9.50943700	-1.83131000	1.03452200
H	10.26683400	-1.64725000	1.79152300
C	9.73464900	-2.79907300	0.06013400
H	10.66327700	-3.36398400	0.05954100
C	8.77068500	-3.04367300	-0.91319600
H	8.95425900	-3.80097600	-1.67041400
C	7.56666000	-2.33024800	-0.93378200
C	8.10539400	-0.04062500	2.14059600
H	7.13276000	0.43027700	1.96727800
C	9.16411800	1.07577400	2.06479300
H	9.16602900	1.54297000	1.07441800
H	10.17175500	0.68957700	2.25842300
H	8.95655000	1.85062600	2.81272500
C	8.05093400	-0.68413100	3.53944000
H	7.83674600	0.07451400	4.30205500
H	9.00453600	-1.15806000	3.80050600
H	7.26966400	-1.44997800	3.58344500
C	6.53194400	-2.62274700	-2.01420500
H	5.67212500	-1.96644500	-1.84761700
C	7.07929300	-2.29911000	-3.41747300
H	7.40900600	-1.25649800	-3.47441200
H	6.30437600	-2.45954500	-4.17683700
H	7.93262100	-2.93820400	-3.67334700
C	6.01593900	-4.07133300	-1.92108500
H	5.60093700	-4.27315800	-0.92800600
H	6.81698000	-4.79663700	-2.10624200
H	5.23061100	-4.24627000	-2.66636600

Table S18. Cartesian coordinates of geometry-optimized structure of *P*-**1h** (B3LYP/6-31G(d)).

Sum of electronic and thermal Free Energies = –2565.463289

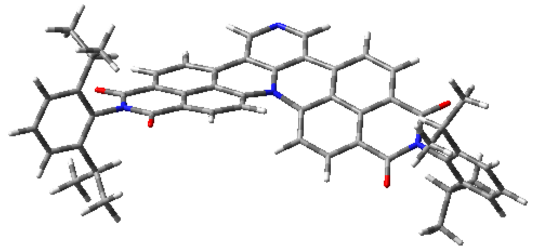

O	5.03549500	-2.43174900	-0.80508000
O	7.20022600	1.24655300	0.86627600
O	-7.20022700	1.24655100	-0.86628100
O	-5.03549500	-2.43174700	0.80508300
N	0.00000000	5.74419300	-0.00001100
N	0.00000000	1.53263700	-0.00000300
N	6.12166900	-0.59625300	0.03476000
N	-6.12167100	-0.59625300	-0.03475800
C	1.29840100	-0.44064600	-0.71222700
H	0.40082900	-0.94088100	-1.05101800
C	2.52431200	-1.11353400	-0.79380900
H	2.56816900	-2.12253100	-1.19035800
C	3.69858100	-0.52112700	-0.35872400
C	3.67019200	0.81769600	0.09747100
C	2.43882300	1.53628700	0.13308100
C	4.86281000	1.46303200	0.50610000
C	4.82714200	2.78685900	0.91599900
H	5.75280200	3.25199600	1.23854200
C	3.63182000	3.51692200	0.88160600
H	3.64719600	4.56032900	1.17611000
C	2.44297600	2.92183800	0.46301100
C	1.19367300	3.64831300	0.24339900
C	0.00000000	2.93383900	-0.00000600
C	1.11866900	5.04819600	0.20549600
H	2.02051200	5.63923800	0.34076700
C	-1.11866900	5.04819500	-0.20551500
H	-2.02051100	5.63923700	-0.34078900
C	-1.19367400	3.64831200	-0.24341300
C	-2.44297700	2.92183600	-0.46302200
C	-2.43882400	1.53628700	-0.13308700
C	-3.63182100	3.51692000	-0.88161900
H	-3.64719700	4.56032600	-1.17612600
C	-4.82714300	2.78685700	-0.91600900
H	-5.75280300	3.25199300	-1.23855300
C	-4.86281100	1.46303100	-0.50610700
C	-3.67019300	0.81769600	-0.09747600
C	-3.69858200	-0.52112500	0.35872300
C	-2.52431400	-1.11353100	0.79381000
H	-2.56817000	-2.12252700	1.19036100
C	-1.29840200	-0.44064300	0.71222600
H	-0.40083000	-0.94087800	1.05101800
C	-1.23021300	0.85181300	0.19456700
C	1.23021200	0.85181200	-0.19457200
C	4.96857200	-1.27777200	-0.40693400
C	6.15350900	0.73452000	0.49662800
C	-6.15351000	0.73452000	-0.49663200
C	-4.96857300	-1.27777100	0.40693600
C	7.37899200	-1.32388200	0.00734100
C	8.16747100	-1.26905900	-1.15509800
C	9.37383600	-1.97866600	-1.15640700
H	10.00455600	-1.95551500	-2.04081300
C	9.77763400	-2.71227900	-0.04496700
H	10.71814200	-3.25673800	-0.06547400
C	8.97710000	-2.74880400	1.09271900
H	9.29970300	-3.32388700	1.95627200
C	7.76175900	-2.05661000	1.14425500
C	7.75481000	-0.47593600	-2.38966200
H	6.79236700	0.00148600	-2.18077400
C	7.54407800	-1.39898900	-3.60500200
H	6.80255800	-2.17279400	-3.38061300
H	8.47561700	-1.89782000	-3.89722900
H	7.19176500	-0.82104200	-4.46796600
C	8.75893900	0.65195200	-2.69459600
H	8.41668800	1.24808100	-3.54918600
H	9.74913300	0.25266000	-2.94365400
H	8.87051400	1.31680000	-1.83166300
C	6.90901100	-2.11773100	2.40620800
H	6.01039400	-1.51587700	2.23832900
C	6.43905600	-3.55572000	2.69737100
H	5.77980800	-3.57286700	3.57370300
H	7.28525700	-4.22098700	2.90547800
H	5.88990600	-3.96553500	1.84320000
C	7.64434900	-1.50071700	3.61121300
H	6.99593000	-1.49984200	4.49575500
H	7.94043700	-0.46826200	3.39820600
H	8.54888800	-2.06669700	3.86309600
C	-7.37899400	-1.32388000	-0.00733600
C	-7.76175800	-2.05661700	-1.14424600
C	-8.97709900	-2.74880800	-1.09270900
H	-9.29970000	-3.32390000	-1.95625700

C	-9.77763900	-2.71227300	0.04497400
H	-10.71814700	-3.25673000	0.06548200
C	-9.37384300	-1.97865200	1.15640900
H	-10.00456700	-1.95549100	2.04081300
C	-8.16747800	-1.26904600	1.15510000
C	-6.90900100	-2.11775400	-2.40619200
H	-6.01039600	-1.51588000	-2.23832300
C	-7.64433900	-1.50078400	-3.61122000
H	-8.54886600	-2.06678600	-3.86309800
H	-6.99591200	-1.49992000	-4.49575500
H	-7.94044700	-0.46832900	-3.39824200
C	-6.43902000	-3.55574400	-2.69731200
H	-7.28521000	-4.22102900	-2.90540500
H	-5.88987000	-3.96552500	-1.84312600
H	-5.77976700	-3.57290500	-3.57364000
C	-7.75482000	-0.47591500	2.38966000
H	-6.79237900	0.00151000	2.18076900
C	-8.75895300	0.65197000	2.69458800
H	-8.87053000	1.31681500	1.83165300
H	-8.41670400	1.24810400	3.54917600
H	-9.74914500	0.25267600	2.94364800
C	-7.54408500	-1.39896200	3.60500300
H	-7.19177400	-0.82101000	4.46796600
H	-6.80256200	-2.17276500	3.38061800
H	-8.47562200	-1.89779500	3.89723200

Table S19. Cartesian coordinates of geometry-optimized structure of *M*-**1h** (B3LYP/6-31G(d)).

Sum of electronic and thermal Free Energies = –2565.463285

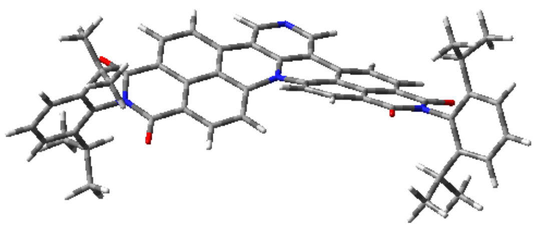

O	-5.03540900	-2.43204400	-0.80427000
O	-7.20029900	1.24679900	0.86566400
O	7.20031600	1.24676700	-0.86578500
O	5.03542600	-2.43199500	0.80432700
N	0.00000200	5.74424400	-0.00018400
N	0.00000700	1.53269000	-0.00007500
N	-6.12169400	-0.59621900	0.03469400
N	6.12171900	-0.59619600	-0.03468200
C	-1.29842700	-0.44063400	-0.71238100
H	-0.40089000	-0.94098000	-1.05108400
C	-2.52434200	-1.11358700	-0.79379400
H	-2.56813700	-2.12264800	-1.19018500
C	-3.69860200	-0.52115200	-0.35878200
C	-3.67020100	0.81774900	0.09721900
C	-2.43884000	1.53636100	0.13273900
C	-4.86283900	1.46315000	0.50567100
C	-4.82719700	2.78702500	0.91544800
H	-5.75288800	3.25219200	1.23785200
C	-3.63186600	3.51705500	0.88110300
H	-3.64719500	4.56047900	1.17555900
C	-2.44300700	2.92190600	0.46262500
C	-1.19370200	3.64836900	0.24304800
C	0.00000600	2.93389600	-0.00011100
C	-1.11871100	5.04825200	0.20509000
H	-2.02061200	5.63927700	0.34007100
C	1.11871700	5.04824500	-0.20542200
H	2.02061600	5.63926400	-0.34043300
C	1.19371100	3.64836000	-0.24330600
C	2.44301900	2.92188900	-0.46284200
C	2.43885600	1.53636100	-0.13288300
C	3.63187700	3.51701900	-0.88134900
H	3.64720300	4.56042900	-1.17585900
C	4.82721000	2.78699200	-0.91565300
H	5.75290000	3.25214500	-1.23808000
C	4.86285600	1.46313900	-0.50580400
C	3.67021800	0.81775600	-0.09732200
C	3.69862300	-0.52112200	0.35874800
C	2.52436300	-1.11354000	0.79378300
H	2.56816000	-2.12258200	1.19022400
C	1.29844600	-0.44059500	0.71233200
H	0.40091000	-0.94092800	1.05105600
C	1.23021000	0.85190800	0.19478000
C	-1.23019300	0.85189500	-0.19489300
C	-4.96856600	-1.27785800	-0.40671400
C	-6.15357600	0.73470800	0.49609400
C	6.15359600	0.73470400	-0.49618200
C	4.96859000	-1.27782000	0.40672700
C	-7.37897500	-1.32392700	0.00755700
C	-7.76141100	-2.05674200	1.14452900
C	-8.97668000	-2.74907600	1.09322700
H	-9.29900500	-3.32427900	1.95680300
C	-9.77748800	-2.71256000	-0.04426900
H	-10.71794200	-3.25711700	-0.06458800
C	-9.37402600	-1.97883900	-1.15575800
H	-10.00497500	-1.95566200	-2.04000100
C	-8.16771600	-1.26913700	-1.15470300
C	-6.90831100	-2.11793100	2.40624000
H	-6.01014300	-1.51538300	2.23845100
C	-6.43735000	-3.55578900	2.69645700
H	-5.88815400	-3.96472100	1.84189800
H	-7.28310000	-4.22169600	2.90435400
H	-5.77788800	-3.57303800	3.57262800
C	-7.64368900	-1.50205900	3.61179700
H	-6.99498300	-1.50121600	4.49612500
H	-8.54776800	-2.06876100	3.86369900
H	-7.94053900	-0.46968000	3.39946100
C	-7.75550300	-0.47575900	-2.38925100
H	-6.79254700	0.00087900	-2.18093900
C	-7.54639300	-1.39824300	-3.60528100
H	-7.19434100	-0.82001100	-4.46816000
H	-8.47846800	-1.89627600	-3.89715100
H	-6.80521400	-2.17269700	-3.38197400
C	-8.75919100	0.65295000	-2.69267700
H	-8.41740200	1.24929300	-3.54730400
H	-8.86951000	1.31739900	-1.82928300
H	-9.74985700	0.25433600	-2.94095400
C	7.37901400	-1.32387500	-0.00747700
C	8.16780800	-1.26886000	1.15473800
C	9.37414600	-1.97851400	1.15585600
H	10.00513600	-1.95516800	2.04006400

C	9.77757900	-2.71241500	0.04447400
H	10.71805300	-3.25693600	0.06484300
C	8.97671700	-2.74915700	-1.09297600
H	9.29901000	-3.32451300	-1.95646300
C	7.76142600	-2.05686500	-1.14434300
C	7.75558500	-0.47535300	2.38920100
H	6.79270600	0.00138900	2.18077600
C	8.75936100	0.65325200	2.69269900
H	9.74995700	0.25455600	2.94112000
H	8.41752700	1.24966500	3.54725900
H	8.86985300	1.31765900	1.82929400
C	7.54625200	-1.39778500	3.60523800
H	8.47825000	-1.89591800	3.89718600
H	6.80500800	-2.17216100	3.38188000
H	7.19419300	-0.81949700	4.46807700
C	6.90822500	-2.11837100	-2.40597100
H	6.01023000	-1.51550900	-2.23838500
C	7.64363500	-1.50325100	-3.61188600
H	7.94078400	-0.47085100	-3.40006100
H	6.99482000	-1.50263200	-4.49613400
H	8.54752900	-2.07030200	-3.86365900
C	6.43687800	-3.55625400	-2.69546100
H	5.77735700	-3.57377400	-3.57158300
H	5.88764500	-3.96462600	-1.84066000
H	7.28246500	-4.22245800	-2.90307800

Table S20. Cartesian coordinates of geometry-optimized structure of the transition state of the racemization of **1h** (B3LYP/6-31G(d)).

Sum of electronic and thermal Free Energies = –2565.439210

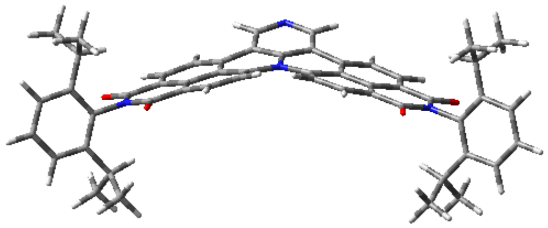

O	5.21398000	-2.26624800	1.26701300
O	7.17401100	1.12386800	-1.10818700
O	-7.16569400	1.10363500	-1.14369400
O	-5.22097300	-2.25170100	1.29293200
N	-0.00294000	5.60977700	-0.89969900
N	-0.00087700	1.53450100	0.30535400
N	6.20324900	-0.58551900	0.06408100
N	-6.20273700	-0.58744300	0.06121500
C	1.48378800	-0.27314600	1.25517700
H	0.71770500	-0.73960800	1.83060000
C	2.72012600	-0.93177600	1.30440100
H	2.80713400	-1.85961000	1.86008400
C	3.82938900	-0.43732300	0.65903100
C	3.72065300	0.81225000	0.00845700
C	2.46830000	1.50936100	-0.02875600
C	4.87369600	1.38480300	-0.58256400
C	4.80616400	2.63709000	-1.17014700
H	5.70108800	3.04752200	-1.62541700
C	3.60868900	3.35228300	-1.15744600
H	3.57910300	4.33002700	-1.62300000
C	2.45726500	2.81669200	-0.57864500
C	1.19892300	3.53651300	-0.52052000
C	-0.00002000	2.84468300	-0.23121900
C	1.12456300	4.90965300	-0.80003900
H	2.04238400	5.47622900	-0.92533500
C	-1.12814300	4.90409600	-0.81442700
H	-2.04759400	5.46551400	-0.95107600
C	-1.19852100	3.53061700	-0.53616600
C	-2.45359100	2.80632400	-0.60404700
C	-2.46844800	1.50648200	-0.03712000
C	-3.59923300	3.33140900	-1.20334900
H	-3.56553600	4.30198900	-1.68358500
C	-4.79623100	2.61502200	-1.21512800
H	-5.68734300	3.01752700	-1.68474800
C	-4.86880800	1.37259800	-0.60735900
C	-3.72035000	0.80917700	0.00100900
C	-3.83235200	-0.43145800	0.66809900
C	-2.72642300	-0.91705100	1.32613300
H	-2.81617300	-1.83753300	1.89347800
C	-1.48940800	-0.25999700	1.27264000
H	-0.72583900	-0.71347700	1.86276000
C	-1.28531100	0.91083000	0.53725500
C	1.28169300	0.90407800	0.52954500
C	5.10509300	-1.18554000	0.70583100
C	6.17099500	0.66579400	-0.58022400
C	-6.16615100	0.65378300	-0.60238000
C	-5.10849100	-1.17891400	0.71747200
C	7.46622800	-1.30316000	0.07762900
C	7.74672300	-2.19679500	-0.97054500
C	8.96963800	-2.87661200	-0.93501600
H	9.21487700	-3.57391000	-1.73132700
C	9.87483300	-2.67411700	0.10233000
H	10.81941100	-3.21177700	0.11207400
C	9.57067700	-1.78329200	1.12729300
H	10.28300600	-1.63117700	1.93348900
C	8.36141500	-1.07873300	1.13789300
C	6.77719300	-2.44262400	-2.12081000
H	5.89299200	-1.81803000	-1.95953900
C	6.29543400	-3.90578400	-2.14535500
H	5.83527100	-4.17931700	-1.19009300
H	7.12418200	-4.59887300	-2.33185800
H	5.55482500	-4.05156600	-2.94098300
C	7.38698300	-2.01792700	-3.47030400
H	6.65615800	-2.14690600	-4.27779200
H	8.26827600	-2.62004800	-3.72071500
H	7.69244200	-0.96667600	-3.44498800
C	8.05974100	-0.11004700	2.27547400
H	7.07511600	0.33192100	2.09359600
C	7.98069600	-0.84109700	3.62927500
H	7.70586200	-0.14035300	4.42693500
H	8.94295500	-1.29140900	3.89988100
H	7.23164600	-1.63913900	3.59640500
C	9.07329700	1.04965500	2.30906700
H	8.80815900	1.76677200	3.09536600
H	9.09043900	1.57820200	1.35022600
H	10.08844700	0.69074200	2.51548300
C	-7.46678000	-1.30318100	0.07987500
C	-7.74297400	-2.21430100	-0.95422400
C	-8.96692200	-2.89199200	-0.91329300

H	-9.20875100	-3.60295000	-1.69850000
C	-9.87750400	-2.67009400	0.11534600
H	-10.82289000	-3.20623900	0.12931100
C	-9.57768600	-1.76184000	1.12620200
H	-10.29416000	-1.59463800	1.92571200
C	-8.36734300	-1.05913300	1.13123500
C	-6.76770500	-2.48123700	-2.09489400
H	-5.88581900	-1.85114200	-1.94258500
C	-7.37214500	-2.08594000	-3.45566800
H	-8.25028600	-2.69574600	-3.69841900
H	-6.63684000	-2.22894900	-4.25668700
H	-7.68093100	-1.03533600	-3.45330000
C	-6.28239000	-3.94347300	-2.08720400
H	-7.10908700	-4.64209000	-2.26183100
H	-5.82469500	-4.19608000	-1.12501100
H	-5.53890400	-4.10412500	-2.87727700
C	-8.06958900	-0.07226000	2.25404700
H	-7.08380300	0.36581500	2.06900500
C	-9.08216300	1.08877900	2.26513100
H	-9.09563100	1.60173600	1.29779500
H	-8.81900200	1.81836000	3.04054600
H	-10.09834200	0.73422800	2.47402100
C	-7.99660300	-0.78146400	3.61976400
H	-7.72397800	-0.06831900	4.40713100
H	-7.24839700	-1.58079900	3.60256300
H	-8.96039800	-1.22637700	3.89383400

Table S21. Cartesian coordinates of geometry-optimized structure of *P*-**1i** (B3LYP/6-31G(d)).

Sum of electronic and thermal Free Energies = –2565.466682

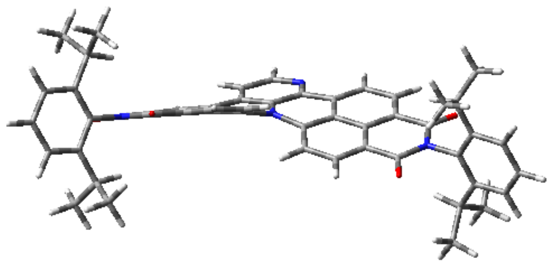

O	5.04096100	-2.46660400	-0.71799200
O	7.21309400	1.28565200	0.76782000
O	-7.19946700	1.24726100	-0.89155600
O	-5.05963100	-2.42105300	0.83314600
N	1.22854200	4.97727800	0.25937100
N	-0.00343800	1.51051600	-0.00307200
N	6.12882800	-0.59413300	0.03366100
N	-6.13342900	-0.59093200	-0.03397300
C	1.29610500	-0.48500600	-0.65706700
H	0.40021700	-0.99736600	-0.98156100
C	2.52364100	-1.15654900	-0.72539700
H	2.56734900	-2.17716000	-1.09107900
C	3.70059300	-0.54668400	-0.32245200
C	3.67164200	0.80665400	0.08896900
C	2.43539400	1.51711600	0.12338800
C	4.86707300	1.47774900	0.44599700
C	4.83590800	2.82262500	0.78865900
H	5.76900100	3.30727600	1.05645700
C	3.63572500	3.54324800	0.75051600
H	3.61788800	4.60693200	0.95678700
C	2.44487000	2.90857300	0.41093600
C	1.19174200	3.63796200	0.22570400
C	-0.00268200	2.91840200	-0.01727200
C	0.09913300	5.65129600	0.04283800
H	0.16910000	6.73584000	0.08778400
C	-1.11788300	5.03442900	-0.22441100
H	-2.00454500	5.63900600	-0.37686900
C	-1.19375000	3.63611400	-0.26153300
C	-2.44139900	2.90540700	-0.48240900
C	-2.43989200	1.52405900	-0.13951100
C	-3.62620600	3.50347400	-0.90660800
H	-3.63502600	4.54604500	-1.20515000
C	-4.82355100	2.77736000	-0.94289200
H	-5.74706200	3.24171000	-1.27246200
C	-4.86305200	1.45723100	-0.52173300
C	-3.67483400	0.81077900	-0.10102900
C	-3.71190200	-0.52208200	0.37045700
C	-2.54127800	-1.11255500	0.82017100
H	-2.59125400	-2.11537200	1.23147400
C	-1.31285100	-0.44663900	0.73592400
H	-0.41997000	-0.94568300	1.08825600
C	-1.23333300	0.83918000	0.19998700
C	1.22547000	0.82240500	-0.17651800
C	4.97304500	-1.29830800	-0.36325700
C	6.16148800	0.75511100	0.44034500
C	-6.15760100	0.73334000	-0.51056900
C	-4.98423800	-1.27231900	0.42169400
C	7.38926400	-1.31621000	0.01558300
C	8.16501500	-1.29555100	-1.15647900
C	9.37490100	-1.99907800	-1.14812700
H	9.99620900	-2.00091400	-2.03949800
C	9.79416200	-2.69497000	-0.01829700
H	10.73704900	-3.23557400	-0.03158200
C	9.00603300	-2.69849300	1.12859400
H	9.34050100	-3.24428900	2.00655300
C	7.78817700	-2.01010100	1.17116100
C	7.73636000	-0.54223900	-2.41034200
H	6.76815900	-0.07287200	-2.21025400
C	7.53389200	-1.49944400	-3.60025200
H	8.47098500	-1.99266200	-3.88418600
H	7.16977500	-0.94896700	-4.47618400
H	6.80419500	-2.27753100	-3.35286400
C	8.72408700	0.59081200	-2.74834900
H	9.71896200	0.19804600	-2.98929300
H	8.82840500	1.27951400	-1.90349700
H	8.37162500	1.15935500	-3.61750500
C	6.94888700	-2.03492900	2.44333000
H	6.04906600	-1.43709100	2.26803900
C	6.48117200	-3.46399900	2.77878900
H	5.92277500	-3.89611400	1.94181100
H	5.83127900	-3.45671900	3.66226100
H	7.32934200	-4.12396800	2.99579700
C	7.69668700	-1.38534300	3.62320300
H	8.60335300	-1.94476000	3.88207500
H	7.05701400	-1.35910100	4.51370700
H	7.99126900	-0.35940300	3.37878500
C	-7.39377700	-1.31323700	-0.00389400
C	-7.77520900	-2.05604000	-1.13469200
C	-8.99372700	-2.74245900	-1.08077500

H	-9.31542900	-3.32507000	-1.93959800
C	-9.79839700	-2.69095200	0.05340100
H	-10.74124500	-3.23126300	0.07587500
C	-9.39558400	-1.94796400	1.15896100
H	-10.02930300	-1.91361300	2.04085500
C	-8.18626200	-1.24343100	1.15502200
C	-6.91763300	-2.13421800	-2.39242900
H	-6.01665100	-1.53521900	-2.22699300
C	-7.64501900	-1.52598700	-3.60669800
H	-6.99303600	-1.53740200	-4.48853600
H	-7.93692100	-0.48996100	-3.40551900
H	-8.55130500	-2.09011200	-3.85645500
C	-6.45365600	-3.57741100	-2.66697200
H	-5.79089000	-3.60670900	-3.54030500
H	-7.30228000	-4.24059600	-2.87181800
H	-5.91002100	-3.98119300	-1.80643500
C	-7.77401500	-0.44104000	2.38373000
H	-6.81096400	0.03392200	2.17201100
C	-7.56496300	-1.35545400	3.60592500
H	-6.82384300	-2.13131900	3.38756000
H	-8.49719300	-1.85148400	3.90071600
H	-7.21296400	-0.77163300	4.46506300
C	-8.77717300	0.69000700	2.67982200
H	-8.43457400	1.29214600	3.53005200
H	-9.76785000	0.29372700	2.93168100
H	-8.88796000	1.34858400	1.81195400

Table S22. Cartesian coordinates of geometry-optimized structure of *M*-**1i** (B3LYP/6-31G(d)).

Sum of electronic and thermal Free Energies = –2565.466683

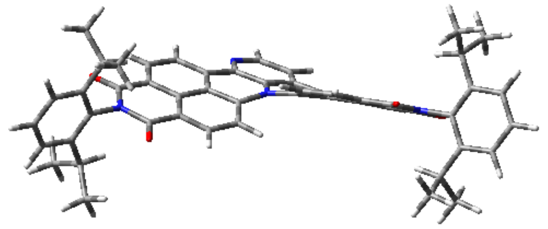

O	-5.04098500	-2.46655600	-0.71818500
O	-7.21310300	1.28555200	0.76802900
O	7.19940900	1.24717000	-0.89169700
O	5.05967700	-2.42100200	0.83344500
N	-1.22854000	4.97720900	0.25972000
N	0.00342600	1.51045400	-0.00288400
N	-6.12883900	-0.59415500	0.03366300
N	6.13340000	-0.59098500	-0.03399100
C	-1.29609300	-0.48503900	-0.65690300
H	-0.40018500	-0.99735600	-0.98141400
C	-2.52363600	-1.15655600	-0.72533200
H	-2.56736100	-2.17713100	-1.09111300
C	-3.70058900	-0.54671000	-0.32236300
C	-3.67164900	0.80659500	0.08917100
C	-2.43540200	1.51704400	0.12365700
C	-4.86707500	1.47766300	0.44626700
C	-4.83589600	2.82251200	0.78903100
H	-5.76898400	3.30714600	1.05688400
C	-3.63571200	3.54314000	0.75091600
H	-3.61788400	4.60681100	0.95725700
C	-2.44486300	2.90849300	0.41126300
C	-1.19173900	3.63789200	0.22603400
C	0.00267100	2.91834000	-0.01705200
C	-0.09914400	5.65123100	0.04312700
H	-0.16909900	6.73577400	0.08813400
C	1.11785600	5.03437400	-0.22421500
H	2.00450000	5.63896600	-0.37671600
C	1.19372300	3.63605800	-0.26139400
C	2.44136200	2.90535000	-0.48232900
C	2.43987400	1.52399300	-0.13945500
C	3.62615100	3.50340600	-0.90659400
H	3.63496900	4.54597700	-1.20513300
C	4.82349000	2.77728000	-0.94294600
H	5.74698400	3.24162000	-1.27258100
C	4.86301200	1.45715700	-0.52177800
C	3.67481400	0.81072000	-0.10099000
C	3.71188300	-0.52212400	0.37054400
C	2.54127200	-1.11259100	0.82030000
H	2.59128000	-2.11538600	1.23165500
C	1.31283700	-0.44670200	0.73602300
H	0.41994400	-0.94571900	1.08836900
C	1.23333000	0.83909700	0.20005600
C	-1.22548300	0.82233100	-0.17626100
C	-4.97305200	-1.29831100	-0.36328300
C	-6.16148400	0.75501600	0.44059700
C	6.15754300	0.73323400	-0.51072400
C	4.98423100	-1.27234100	0.42181100
C	-7.38929900	-1.31618800	0.01538600
C	-7.78837900	-2.01014000	1.17087000
C	-9.00626500	-2.69846500	1.12811700
H	-9.34086600	-3.24429600	2.00600300
C	-9.79426000	-2.69483000	-0.01886800
H	-10.73717200	-3.23538700	-0.03229600
C	-9.37483300	-1.99888600	-1.14860300
H	-9.99603300	-2.00063800	-2.04004900
C	-8.16491400	-1.29541000	-1.15676400
C	-6.94924500	-2.03507400	2.44313800
H	-6.04930700	-1.43738200	2.26794100
C	-6.48178800	-3.46420400	2.77869700
H	-7.33007900	-4.12404500	2.99562200
H	-5.83200400	-3.45699300	3.66224900
H	-5.92334800	-3.89643100	1.94180500
C	-7.69709800	-1.38533800	3.62289500
H	-8.60389000	-1.94460600	3.88165100
H	-7.99147900	-0.35935900	3.37840400
H	-7.05754500	-1.35917800	4.51348800
C	-7.73606400	-0.54206600	-2.41054000
H	-6.76798900	-0.07253700	-2.21022400
C	-7.53315300	-1.49931400	-3.60034400
H	-6.80339100	-2.27726500	-3.35272200
H	-7.16892100	-0.94884100	-4.47623200
H	-8.47010600	-1.99270000	-3.88444900
C	-8.72386000	0.59082400	-2.74886900
H	-9.71861900	0.19792500	-2.99007200
H	-8.37123500	1.15937300	-3.61795500
H	-8.82850500	1.27956200	-1.90408400
C	7.39375100	-1.31328800	-0.00388200
C	8.18607900	-1.24371400	1.15515700
C	9.39539300	-1.94825900	1.15912500
H	10.02898400	-1.91409700	2.04111800

C	9.79836400	-2.69101500	0.05346600
H	10.74121000	-3.23132700	0.07595900
C	8.99385600	-2.74228100	-1.08083600
H	9.31569800	-3.32468200	-1.93974900
C	7.77533500	-2.05586900	-1.13477400
C	7.77367300	-0.44155900	2.38396500
H	6.81072300	0.03356900	2.17215900
C	8.77689100	0.68930900	2.68053500
H	8.43416800	1.29128900	3.53082700
H	8.88794900	1.34808300	1.81285000
H	9.76747200	0.29287300	2.93252400
C	7.56427800	-1.35624200	3.60590200
H	7.21219000	-0.77258600	4.46511600
H	8.49640100	-1.85244900	3.90074000
H	6.82310900	-2.13196100	3.38720000
C	6.91803900	-2.13361700	-2.39272900
H	6.01656700	-1.53545600	-2.22692900
C	6.45517100	-3.57686500	-2.66878100
H	5.91166200	-3.98192200	-1.80875900
H	7.30426800	-4.23926900	-2.87417800
H	5.79256800	-3.60574600	-3.54224900
C	7.64529800	-1.52369900	-3.60624400
H	6.99359500	-1.53480900	-4.48829600
H	8.55209400	-2.08692100	-3.85620000
H	7.93635100	-0.48764500	-3.40400500

Table S22. Cartesian coordinates of geometry-optimized structure of the transition state of the racemization of **1i** (B3LYP/6-31G(d)).

Sum of electronic and thermal Free Energies = –2565.442412

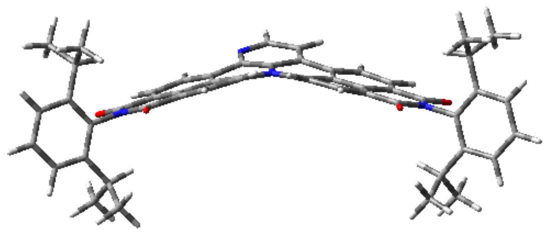

O	-5.18992900	-2.26535300	1.28123200
O	-7.13333000	1.09171400	-1.15484900
O	7.17816700	1.16853700	-1.02164600
O	5.16362600	-2.31730300	1.16206800
N	-1.25724300	4.86326600	-0.80789900
N	0.00225500	1.56733300	0.36339000
N	-6.17097300	-0.59287500	0.05964800
N	6.17837500	-0.59013300	0.04957000
C	-1.46844600	-0.24622700	1.31100000
H	-0.70401800	-0.68103900	1.91773200
C	-2.70189200	-0.91199900	1.34993100
H	-2.78897600	-1.84033700	1.90476900
C	-3.80712400	-0.42988400	0.68646000
C	-3.70223900	0.81672900	0.02937300
C	-2.45521300	1.52024800	0.00442600
C	-4.84737400	1.38440700	-0.58170800
C	-4.78207600	2.64265200	-1.16209700
H	-5.67708200	3.04900200	-1.62104700
C	-3.59044900	3.36761700	-1.13180000
H	-3.53300300	4.36829200	-1.54245600
C	-2.44796400	2.82092100	-0.55255200
C	-1.20195000	3.56262600	-0.48977600
C	0.00331000	2.88927500	-0.16151000
C	-0.12345000	5.55432800	-0.88113800
H	-0.21201400	6.61890500	-1.08568500
C	1.11676200	4.95098300	-0.75481500
H	2.01531000	5.53654100	-0.90445000
C	1.20176400	3.58708800	-0.44028600
C	2.46427600	2.86679800	-0.48093300
C	2.46099600	1.53523600	0.00815000
C	3.63516900	3.43009500	-0.98955000
H	3.62743000	4.43750600	-1.38740900
C	4.82753900	2.70729000	-1.02220100
H	5.73550500	3.13786600	-1.43055700
C	4.87300300	1.42039800	-0.51301800
C	3.70739300	0.82633100	0.03000900
C	3.80109900	-0.44737300	0.63413900
C	2.68714600	-0.94981500	1.26894000
H	2.76745500	-1.89368800	1.79802600
C	1.46222500	-0.27184200	1.25002800
H	0.68241400	-0.73591000	1.81191600
C	1.26993200	0.91351000	0.53324400
C	-1.27785800	0.94325100	0.60216500
C	-5.07829300	-1.18546900	0.71866900
C	-6.13722400	0.65235200	-0.59879700
C	6.16555900	0.69117300	-0.52996700
C	5.06769100	-1.20932000	0.65412100
C	-7.42935700	-1.31862600	0.05750200
C	-8.31696900	-1.13812500	1.13254500
C	-9.52212100	-1.84935700	1.10679500
H	-10.22597700	-1.73510400	1.92658400
C	-9.83217700	-2.70063500	0.05049800
H	-10.77424200	-3.24282200	0.04794800
C	-8.93659600	-2.85674600	-1.00308100
H	-9.18836300	-3.51964800	-1.82631100
C	-7.71605400	-2.17201800	-1.02210500
C	-8.00442300	-0.22034800	2.30876800
H	-7.06884800	0.30425000	2.09103700
C	-7.78140600	-1.03281200	3.59919900
H	-8.69119800	-1.57053700	3.89177500
H	-7.50409900	-0.36981400	4.42788400
H	-6.98327500	-1.76895900	3.46023600
C	-9.08615200	0.86022000	2.49393600
H	-10.05697800	0.42368200	2.75571700
H	-9.21539100	1.44425400	1.57641900
H	-8.80415000	1.54614900	3.30173800
C	-6.76498000	-2.35366700	-2.19940900
H	-5.84300200	-1.80653600	-1.97915100
C	-6.37067700	-3.82924400	-2.39612900
H	-5.92667700	-4.24052400	-1.48320900
H	-5.63833800	-3.92225800	-3.20703300
H	-7.23513800	-4.44955400	-2.65976900
C	-7.35815400	-1.74366900	-3.48465100
H	-8.27902100	-2.25985800	-3.78139500
H	-6.64564000	-1.82741700	-4.31437600
H	-7.59624200	-0.68554600	-3.33602200
C	7.43427000	-1.32011500	0.03988900
C	8.31747800	-1.16349400	1.12212400
C	9.52020600	-1.87839100	1.08785500

H	10.22299300	-1.77856200	1.91044500
C	9.82980000	-2.71391900	0.01887100
H	10.76918500	-3.26063000	0.01074900
C	8.93653500	-2.84973700	-1.03946500
H	9.18571400	-3.50445800	-1.86996700
C	7.72039000	-2.15706800	-1.05259800
C	8.00866400	-0.25799900	2.30872300
H	7.03543000	0.21124800	2.13400800
C	9.03981500	0.87983500	2.43170700
H	8.76846500	1.55466700	3.25256300
H	9.08720600	1.46283300	1.50595700
H	10.04420600	0.49237200	2.63903300
C	7.88951900	-1.06697600	3.61447800
H	7.61098100	-0.41031900	4.44761400
H	8.83825700	-1.55055100	3.87532300
H	7.12751700	-1.84745500	3.51894000
C	6.76279300	-2.33105000	-2.22577600
H	5.88526500	-1.70259400	-2.04431300
C	6.26136200	-3.78424800	-2.32864400
H	5.78487900	-4.09800700	-1.39397600
H	7.08320100	-4.47888700	-2.53857900
H	5.52945500	-3.88014700	-3.13975000
C	7.39535100	-1.84926600	-3.54524100
H	6.67246800	-1.92652700	-4.36635500
H	8.27038200	-2.45185900	-3.81561800
H	7.71621500	-0.80543700	-3.46417200

Table S23. Cartesian coordinates of geometry-optimized structure of *P*-**1j** (B3LYP/6-31G(d)).

Sum of electronic and thermal Free Energies = –2581.520631

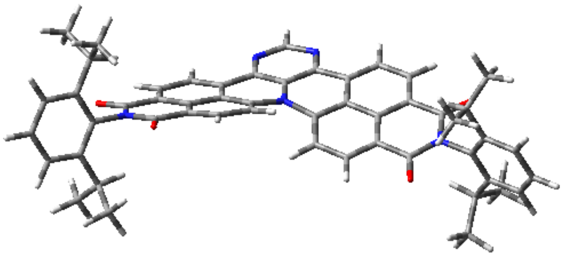

O	-5.09895800	-2.46665000	0.71569000
O	-7.22286300	1.31650800	-0.76088300
O	7.22286300	1.31650800	0.76088200
O	5.09895800	-2.46665100	-0.71569000
N	-6.16452100	-0.57986200	-0.03192400
N	6.16452100	-0.57986200	0.03192400
C	-1.32957000	-0.53421000	0.65745200
H	-0.44304100	-1.05747300	0.98912300
C	-2.56642600	-1.18876500	0.72584800
H	-2.62382100	-2.20795000	1.09356300
C	-3.73462700	-0.56422200	0.32123500
C	-3.68669300	0.78890800	-0.08876300
C	-2.44122500	1.48292900	-0.12477400
C	-4.87488100	1.47709200	-0.44034400
C	-4.83312000	2.82310000	-0.77553000
H	-5.76237500	3.31813200	-1.03709300
C	-3.62472500	3.53036500	-0.73581400
H	-3.59422500	4.59550600	-0.93433800
C	-2.44205700	2.87681800	-0.40665400
C	-1.18034700	3.58433600	-0.22352100
C	0.00000000	2.84691700	0.00000000
C	0.00000000	5.52261900	0.00000000
H	0.00000000	6.61004400	-0.00000100
C	1.18034700	3.58433600	0.22352000
C	2.44205700	2.87681800	0.40665300
C	2.44122500	1.48292900	0.12477300
C	3.62472500	3.53036500	0.73581300
H	3.59422500	4.59550600	0.93433700
C	4.83312000	2.82310000	0.77552900
H	5.76237500	3.31813200	1.03709200
C	4.87488100	1.47709200	0.44034400
C	3.68669300	0.78890800	0.08876300
C	3.73462700	-0.56422200	-0.32123500
C	2.56642600	-1.18876500	-0.72584700
H	2.62382100	-2.20795000	-1.09356300
C	1.32956900	-0.53421000	-0.65745100
H	0.44304100	-1.05747300	-0.98912300
C	1.23795800	0.77058800	-0.17375300
C	-1.23795800	0.77058800	0.17375300
C	-5.01699500	-1.29926000	0.36254500
C	-6.17983000	0.76913400	-0.43514300
C	6.17983000	0.76913400	0.43514300
C	5.01699500	-1.29926100	-0.36254500
C	-7.43450700	-1.28589200	-0.01344500
C	-8.20870000	-1.25572200	1.15943300
C	-9.42753000	-1.94362800	1.15129400
H	-10.04817900	-1.93823300	2.04306000
C	-9.85643600	-2.63322900	0.02123600
H	-10.80617500	-3.16161800	0.03484700
C	-9.06927100	-2.64632700	-1.12623600
H	-9.41135400	-3.18732500	-2.00418200
C	-7.84263300	-1.97375200	-1.16937800
C	-7.76946500	-0.50953100	2.41393600
H	-6.79605400	-0.05100600	2.21354000
C	-7.57652500	-1.47123800	3.60185800
H	-6.85649400	-2.25759300	3.35228300
H	-8.51899200	-1.95379100	3.88616400
H	-7.20480700	-0.92660900	4.47820500
C	-8.74347200	0.63423300	2.75576700
H	-8.38289100	1.19694900	3.62533000
H	-9.74226200	0.25244900	2.99792600
H	-8.84175500	1.32583700	1.91254700
C	-7.00453200	-2.00929200	-2.44211700
H	-6.09691300	-1.42291800	-2.26777000
C	-6.55529900	-3.44429200	-2.77769600
H	-7.41203600	-4.09326200	-2.99401200
H	-6.00189300	-3.88366800	-1.94118400
H	-5.90610100	-3.44536100	-3.66166100
C	-7.74484300	-1.35069200	-3.62175200
H	-8.02684500	-0.32109800	-3.37778400
H	-8.65841900	-1.89894700	-3.88001400
H	-7.10535800	-1.33269800	-4.51254900
C	7.43450700	-1.28589200	0.01344500
C	7.84263400	-1.97375200	1.16937800
C	9.06927100	-2.64632700	1.12623600
H	9.41135500	-3.18732400	2.00418200
C	9.85643600	-2.63322900	-0.02123600
H	10.80617500	-3.16161800	-0.03484600
C	9.42753000	-1.94362900	-1.15129300
H	10.04817800	-1.93823400	-2.04306000

C	8.20870000	-1.25572300	-1.15943300
C	7.00453300	-2.00929100	2.44211800
H	6.09691300	-1.42291800	2.26777000
C	7.74484300	-1.35069000	3.62175100
H	8.02684500	-0.32109600	3.37778300
H	8.65841900	-1.89894500	3.88001400
H	7.10535900	-1.33269600	4.51254900
C	6.55530000	-3.44429200	2.77769700
H	5.90610200	-3.44536000	3.66166300
H	7.41203700	-4.09326100	2.99401400
H	6.00189400	-3.88366800	1.94118600
C	7.76946500	-0.50953100	-2.41393600
H	6.79605300	-0.05100700	-2.21354000
C	8.74347200	0.63423200	-2.75576800
H	8.84175500	1.32583700	-1.91254800
H	8.38289000	1.19694700	-3.62533100
H	9.74226200	0.25244800	-2.99792600
C	7.57652400	-1.47123900	-3.60185800
H	6.85649300	-2.25759400	-3.35228300
H	8.51899100	-1.95379200	-3.88616400
H	7.20480600	-0.92661000	-4.47820500
N	0.00000000	1.44266000	0.00000000
N	1.16813800	4.92617200	0.23437400
N	-1.16813800	4.92617200	-0.23437500

Table S24. Cartesian coordinates of geometry-optimized structure of *M*-**1j** (B3LYP/6-31G(d)).

Sum of electronic and thermal Free Energies = –2581.520631

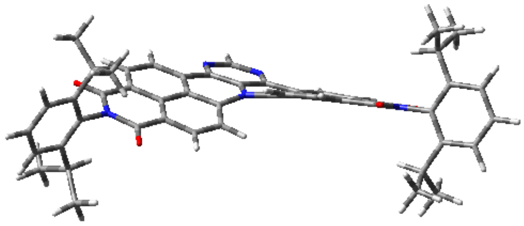

O	-5.09909500	-2.46645400	-0.71615600
O	-7.22283600	1.31645700	0.76129000
O	7.22287700	1.31653800	-0.76064800
O	5.09889100	-2.46678700	0.71538000
N	-1.16810200	4.92616500	0.23479800
N	-0.00002400	1.44264300	-0.00009500
N	-6.16457100	-0.57979800	0.03191500
N	6.16449400	-0.57991200	-0.03195600
C	-1.32967500	-0.53409300	-0.65780400
H	-0.44317900	-1.05731100	-0.98963300
C	-2.56655200	-1.18861500	-0.72622600
H	-2.62399500	-2.20773600	-1.09411300
C	-3.73471200	-0.56411900	-0.32143300
C	-3.68673700	0.78894900	0.08877000
C	-2.44124900	1.48294600	0.12480100
C	-4.87489100	1.47710200	0.44051600
C	-4.83309600	2.82307300	0.77585500
H	-5.76233300	3.31808100	1.03752800
C	-3.62469600	3.53032700	0.73614300
H	-3.59417300	4.59544400	0.93478600
C	-2.44205100	2.87679500	0.40685100
C	-1.18035500	3.58432500	0.22375900
C	-0.00001600	2.84690000	0.00011200
C	0.00003700	5.52260400	0.00042300
H	0.00008800	6.61003700	0.00060600
C	1.18033700	3.58431200	-0.22331500
C	2.44205000	2.87681300	-0.40650300
C	2.44121000	1.48288900	-0.12478500
C	3.62474500	3.53038700	-0.73554600
H	3.59425500	4.59555300	-0.93393300
C	4.83312700	2.82312400	-0.77528900
H	5.76239800	3.31817200	-1.03676400
C	4.87487500	1.47707200	-0.44023900
C	3.68667500	0.78886000	-0.08879200
C	3.73457700	-0.56432100	0.32105600
C	2.56635500	-1.18889800	0.72554200
H	2.62372000	-2.20812200	1.09315400
C	1.32950200	-0.53432900	0.65717200
H	0.44296600	-1.05763700	0.98875300
C	1.23791600	0.77052100	0.17361900
C	-1.23801500	0.77061900	-0.17388900
C	-5.01709600	-1.29913300	-0.36277400
C	-6.17983800	0.76914600	0.43535500
C	6.17982300	0.76912700	-0.43501900
C	5.01694200	-1.29936100	0.36235500
C	-7.43457600	-1.28579800	0.01346600
C	-7.84255400	-1.97389600	1.16931000
C	-9.06923000	-2.64640500	1.12620700
H	-9.41119200	-3.18760300	2.00407700
C	-9.85658300	-2.63299400	-0.02113200
H	-10.80635300	-3.16132900	-0.03470900
C	-9.42782600	-1.94315300	-1.15109900
H	-10.04861500	-1.93752600	-2.04276600
C	-8.20895800	-1.25531500	-1.15927800
C	-7.00420100	-2.00985800	2.44187100
H	-6.09679400	-1.42311500	2.26766200
C	-6.55449400	-3.44493400	2.77652200
H	-7.41102800	-4.09424900	2.99261000
H	-5.90512600	-3.44635200	3.66036400
H	-6.00114000	-3.88366000	1.93963800
C	-7.74442100	-1.35215100	3.62205200
H	-8.65776100	-1.90081900	3.88026400
H	-8.02679600	-0.32250100	3.37874500
H	-7.10470900	-1.33446000	4.51269100
C	-7.76980800	-0.50899600	-2.41373500
H	-6.79657800	-0.05014100	-2.21321900
C	-7.57638000	-1.47077000	-3.60153800
H	-6.85612500	-2.25685600	-3.35176600
H	-7.20473800	-0.92612500	-4.47790700
H	-8.51866200	-1.95366800	-3.88587300
C	-8.74409700	0.63443300	-2.75583000
H	-9.74273200	0.25236600	-2.99817800
H	-8.38350300	1.19719500	-3.62535700
H	-8.84275000	1.32607700	-1.91268400
C	7.43447400	-1.28595100	-0.01345100
C	8.20858800	-1.25589000	1.15948200
C	9.42740000	-1.94382700	1.15137200
H	10.04799900	-1.93849700	2.04317300
C	9.85635300	-2.63337500	0.02130000
H	10.80607200	-3.16179800	0.03493900

C	9.06926000	-2.64637600	-1.12622200
H	9.41138300	-3.18732800	-2.00418100
C	7.84265000	-1.97375200	-1.16940200
C	7.76931900	-0.50972500	2.41399000
H	6.79579500	-0.05140800	2.21366300
C	8.74316500	0.63424000	2.75562200
H	8.38260800	1.19695300	3.62519800
H	8.84121700	1.32579500	1.91233700
H	9.74204800	0.25262300	2.99766300
C	7.57666600	-1.47136200	3.60201100
H	7.20490200	-0.92671700	4.47832700
H	8.51923800	-1.95370800	3.88631600
H	6.85676500	-2.25788100	3.35257400
C	7.00471100	-2.00904400	-2.44225400
H	6.09686900	-1.42305700	-2.26776900
C	6.55598900	-3.44401400	-2.77859900
H	6.00259900	-3.88397700	-1.94238400
H	7.41293600	-4.09262800	-2.99514300
H	5.90690500	-3.44483700	-3.66264900
C	7.74501400	-1.34962800	-3.62144900
H	7.10570400	-1.33143300	-4.51236800
H	8.65883200	-1.89745900	-3.87976300
H	8.02662400	-0.32005700	-3.37693400
N	1.16816200	4.92616100	-0.23401900

Table S25. Cartesian coordinates of geometry-optimized structure of the transition state of the racemization of **1j** (B3LYP/6-31G(d)).

Sum of electronic and thermal Free Energies = –2581.489212

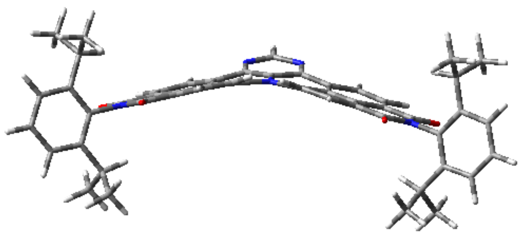

O	-5.18678000	-2.26465400	1.28378700
O	-7.13172800	1.08884900	-1.15596400
O	7.17974500	1.17173000	-1.02380500
O	5.16678800	-2.31246600	1.16399000
N	-1.25717500	4.86321700	-0.81367800
N	0.00376100	1.56913200	0.36125900
N	-6.16859500	-0.59396600	0.06037500
N	6.18075000	-0.58613900	0.04946900
C	-1.46612900	-0.24396000	1.31102000
H	-0.70148100	-0.67776900	1.91819300
C	-2.69929700	-0.91019700	1.35078700
H	-2.78596100	-1.83794200	1.90668200
C	-3.80477200	-0.42929000	0.68684400
C	-3.70044500	0.81662200	0.02833900
C	-2.45371100	1.52062700	0.00251200
C	-4.84585500	1.38313500	-0.58330800
C	-4.78111500	2.64074800	-1.16512500
H	-5.67631900	3.04620900	-1.62447500
C	-3.58978500	3.36623900	-1.13572900
H	-3.53278000	4.36647200	-1.54752200
C	-2.44703600	2.82067100	-0.55593900
C	-1.20132400	3.56296100	-0.49408600
C	0.00423500	2.89047900	-0.16513800
C	-0.12367300	5.55466300	-0.88777500
H	-0.21269000	6.61897100	-1.09352100
C	1.20238300	3.58847100	-0.44478400
C	2.46518900	2.86865600	-0.48470000
C	2.46249100	1.53764800	0.00589100
C	3.63581600	3.43186000	-0.99403300
H	3.62763400	4.43881700	-1.39303200
C	4.82848200	2.70951100	-1.02594500
H	5.73624300	3.13999900	-1.43484900
C	4.87451100	1.42321500	-0.51530800
C	3.70918200	0.82928200	0.02846900
C	3.80345400	-0.44369800	0.63403500
C	2.68975100	-0.94588000	1.26947900
H	2.77048400	-1.88912000	1.79962800
C	1.46454900	-0.26843500	1.24988100
H	0.68496700	-0.73218800	1.81234600
C	1.27171900	0.91602500	0.53176800
C	-1.27607900	0.94479300	0.60082600
C	-5.07562700	-1.18536200	0.71999400
C	-6.13540400	0.65052800	-0.59948100
C	6.16736700	0.69450500	-0.53151800
C	5.07036200	-1.20509900	0.65479500
C	-7.42668000	-1.32023800	0.05913500
C	-8.31429500	-1.13888600	1.13403300
C	-9.51915500	-1.85064400	1.10916900
H	-10.22300300	-1.73575400	1.92887500
C	-9.82892900	-2.70324600	0.05385700
H	-10.77077100	-3.24582400	0.05198400
C	-8.93335400	-2.86018000	-0.99960400
H	-9.18490200	-3.52411800	-1.82206600
C	-7.71309600	-2.17497000	-1.01948500
C	-8.00204900	-0.21964900	2.30919500
H	-7.06670600	0.30508800	2.09080700
C	-7.77861100	-1.03055900	3.60053000
H	-8.68816200	-1.56832800	3.89377600
H	-7.50152300	-0.36650900	4.42844500
H	-6.98018600	-1.76653300	3.46234700
C	-9.08421200	0.86068100	2.49321100
H	-10.05484000	0.42404000	2.75555100
H	-9.21375300	1.44362300	1.57504200
H	-8.80244000	1.54764100	3.30021700
C	-6.76202500	-2.35756000	-2.19664700
H	-5.84025800	-1.80979900	-1.97707000
C	-6.36712600	-3.83319600	-2.39172200
H	-5.92289600	-4.24325900	-1.47836600
H	-5.63480300	-3.92682600	-3.20256900
H	-7.23134800	-4.45416100	-2.65460100
C	-7.35553600	-1.74926300	-3.48253900
H	-8.27621000	-2.26616700	-3.77863700
H	-6.64304300	-1.83365600	-4.31221600
H	-7.59405100	-0.69107000	-3.33509200
C	7.43694600	-1.31561300	0.04053100
C	8.32016100	-1.15740200	1.12252900
C	9.52318100	-1.87184100	1.08898900
H	10.22598200	-1.77079000	1.91141800
C	9.83304900	-2.70845100	0.02093100

H	10.77265900	-3.25478300	0.01336500
C	8.93977000	-2.84583600	-1.03719100
H	9.18916400	-3.50139400	-1.86696800
C	7.72333900	-2.15368400	-1.05102700
C	8.01105200	-0.25069100	2.30812200
H	7.03761200	0.21795600	2.13294100
C	9.04174100	0.88770700	2.42974900
H	8.77016700	1.56335600	3.24985800
H	9.08883000	1.46967600	1.50333600
H	10.04630600	0.50089400	2.63744600
C	7.89232700	-1.05823800	3.61480000
H	7.61357400	-0.40075300	4.44721100
H	8.84128200	-1.54112600	3.87612900
H	7.13064100	-1.83913900	3.52019700
C	6.76573600	-2.32939000	-2.22394300
H	5.88796000	-1.70109100	-2.04313300
C	6.26489800	-3.78291000	-2.32513200
H	5.78860600	-4.09580700	-1.39007800
H	7.08700900	-4.47744700	-2.53433500
H	5.53297600	-3.88003000	-3.13608000
C	7.39800700	-1.84883900	-3.54399500
H	6.67510200	-1.92732800	-4.36497300
H	8.27326900	-2.45137700	-3.81374800
H	7.71844600	-0.80478700	-3.46413000
N	1.11679700	4.95197400	-0.76085200

Table S26. Cartesian coordinates of geometry-optimized structure of *P*-[4]helicene (B3LYP/6-31G(d)).

Sum of electronic and thermal Free Energies = –692.968364

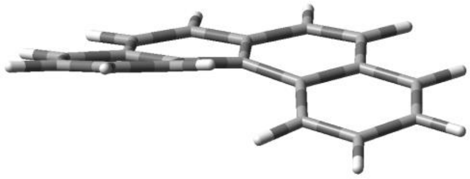

C	-3.85812400	-1.51427800	0.10432200
C	-2.70442900	-2.24084200	0.45582600
C	-1.46020800	-1.63936700	0.43549500
C	-1.28904500	-0.28025800	0.05669700
C	-2.48367200	0.47602700	-0.18329600
C	-3.74269900	-0.17230700	-0.18953200
C	0.00000000	0.39510700	-0.00000300
C	0.00000000	1.81355500	-0.00000100
C	-1.21784700	2.53872600	-0.18858800
C	-2.40676400	1.89318300	-0.34170900
C	1.28904500	-0.28025700	-0.05670000
C	2.48367100	0.47602700	0.18329500
C	2.40676300	1.89318400	0.34171000
C	1.21784500	2.53872600	0.18858900
C	1.46020900	-1.63936700	-0.43549600
C	2.70443100	-2.24084100	-0.45582500
C	3.85812500	-1.51427700	-0.10432000
C	3.74269800	-0.17230600	0.18953400
H	-4.83165400	-1.99667800	0.10565500
H	-2.79103200	-3.27901600	0.76519800
H	-0.60670100	-2.21079500	0.77483800
H	-4.62787400	0.42267600	-0.40244500
H	-1.16732100	3.62395400	-0.23241500
H	-3.32310300	2.44855200	-0.52592000
H	3.32310100	2.44855200	0.52592400
H	1.16731900	3.62395400	0.23241800
H	0.60670300	-2.21079600	-0.77484100
H	2.79103400	-3.27901500	-0.76519700
H	4.83165500	-1.99667600	-0.10565000
H	4.62787300	0.42267800	0.40245000

Table S27. Cartesian coordinates of geometry-optimized structure of *M*-[4]helicene (B3LYP/6-31G(d)).

Sum of electronic and thermal Free Energies = –692.968364

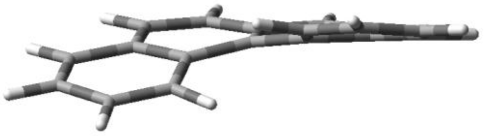

C	3.85811200	-1.51431000	0.10408200
C	2.70443300	-2.24089000	0.45564200
C	1.46023900	-1.63937900	0.43552800
C	1.28905600	-0.28024300	0.05679200
C	2.48368500	0.47603500	-0.18316300
C	3.74269900	-0.17230800	-0.18959700
C	0.00000000	0.39513500	0.00000000
C	0.00000000	1.81354700	0.00000100
C	1.21788600	2.53875600	-0.18828600
C	2.40680000	1.89322800	-0.34132100
C	-1.28905500	-0.28024300	-0.05679200
C	-2.48368500	0.47603500	0.18316200
C	-2.40680000	1.89322800	0.34132000
C	-1.21788600	2.53875600	0.18828700
C	-1.46023900	-1.63937900	-0.43552700
C	-2.70443300	-2.24089000	-0.45564100
C	-3.85811200	-1.51431000	-0.10408300
C	-3.74269900	-0.17230800	0.18959600
H	4.83160300	-1.99679700	0.10513300
H	2.79107900	-3.27907600	0.76495700
H	0.60672800	-2.21079800	0.77486800
H	4.62786000	0.42268700	-0.40253900
H	1.16737100	3.62399100	-0.23193400
H	3.32319500	2.44860700	-0.52526600
H	-3.32319500	2.44860700	0.52526400
H	-1.16737100	3.62399100	0.23193500
H	-0.60672800	-2.21079800	-0.77486700
H	-2.79107900	-3.27907600	-0.76495600
H	-4.83160300	-1.99679700	-0.10513400
H	-4.62786000	0.42268700	0.40253700

Table S28. Cartesian coordinates of geometry-optimized structure of the transition state of the racemization of [4]helicene (B3LYP/6-31G(d)).

Sum of electronic and thermal Free Energies = –692.961126

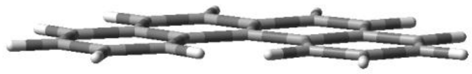

C	0.00000000	3.98653600	-1.44457500
C	-0.00021200	2.84690500	-2.26409000
C	-0.00014300	1.57597800	-1.71995200
C	0.00012900	1.32278500	-0.31859400
C	0.00034200	2.51118900	0.49819100
C	0.00031400	3.80386300	-0.08134800
C	0.00000000	0.00000000	0.33481100
C	0.00000000	0.00000000	1.76139600
C	0.00029700	1.20878600	2.52246400
C	0.00050900	2.42215200	1.91920000
C	-0.00012900	-1.32278500	-0.31859400
C	-0.00034200	-2.51118900	0.49819100
C	-0.00050900	-2.42215200	1.91920000
C	-0.00029700	-1.20878600	2.52246400
C	0.00014300	-1.57597800	-1.71995200
C	0.00021200	-2.84690500	-2.26409000
C	0.00000000	-3.98653600	-1.44457500
C	-0.00031400	-3.80386300	-0.08134800
H	-0.00008400	4.98351600	-1.87589800
H	-0.00043100	2.95401200	-3.34562100
H	-0.00010900	0.77404700	-2.43203900
H	0.00052100	4.65969700	0.58953800
H	0.00034600	1.12754100	3.60630600
H	0.00072500	3.34014500	2.50130900
H	-0.00072500	-3.34014500	2.50130900
H	-0.00034600	-1.12754100	3.60630600
H	0.00010900	-0.77404700	-2.43203900
H	0.00043100	-2.95401200	-3.34562100
H	0.00008400	-4.98351600	-1.87589800
H	-0.00052100	-4.65969700	0.58953800

10. References

S1. J. R. Lakowicz, *Principles of Fluorescence Spectroscopy*, Kluwer Academic/Plenum Publishers, New York, **1999**.

S2. Gaussian 16, Revision B.01, M. J. Frisch, G. W. Trucks, H. B. Schlegel, G. E. Scuseria, M. A. Robb, J. R. Cheeseman, G. Scalmani, V. Barone, G. A. Petersson, H. Nakatsuji, X. Li, M. Caricato, A. V. Marenich, J. Bloino, B. G. Janesko, R. Gomperts, B. Mennucci, H. P. Hratchian, J. V. Ortiz, A. F. Izmaylov, J. L. Sonnenberg, D. Williams-Young, F. Ding, F. Lipparini, F. Egidi, J. Goings, B. Peng, A. Petrone, T. Henderson, D. Ranasinghe, V. G. Zakrzewski, J. Gao, N. Rega, G. Zheng, W. Liang, M. Hada, M. Ehara, K. Toyota, R. Fukuda, J. Hasegawa, M. Ishida, T. Nakajima, Y. Honda, O. Kitao, H. Nakai, T. Vreven, K. Throssell, J. A. Montgomery, Jr., J. E. Peralta, F. Ogliaro, M. J. Bearpark, J. J. Heyd, E. N. Brothers, K. N. Kudin, V. N. Staroverov, T. A. Keith, R. Kobayashi, J. Normand, K. Raghavachari, A. P. Rendell, J. C. Burant, S. S. Iyengar, J. Tomasi, M. Cossi, J. M. Millam, M. Klene, C. Adamo, R. Cammi, J. W. Ochterski, R. L. Martin, K. Morokuma, O. Farkas, J. B. Foresman, and D. J. Fox, Gaussian, Inc., Wallingford CT, **2016**.

S3. W. Kabsch, *Acta Crystallogr. D*, **2010**, *66*, 125–132.

S4. G. M. Sheldrick, *Acta Crystallogr. A*, **2015**, *71*, 3–8.

S5. G. M. Sheldrick, *Acta crystallogr. C*, **2015**, *71*, 3–8.

S6. A. L. Spek, *J. Appl. Crystallogr.* **2003**, *36*, 7–13.

S7. Z. Sun, K.-W. Huang, J. Wu, *Org. Lett*. **2010**, *12*, 4690–4694.

S8. S. M. Kim, S. Y. Byeon, S. H. Hwang, J. Y. Lee, *Chem. Commun*. **2015**, *51*, 10672–10675.

S9. K. Shoyama, D. Schmidt, M. Mahl, F. Würthner, *Org. Lett*. **2017**, *19*, 5328–5331.

S10. S. Seifert, D. Schmidt, F. Würthner, *Org. Chem. Front.* **2016**, *3*, 1435–1442.

S11. J. Cremer, E. Mena-Osteritz, N. G. Pschierer, K. Müllen and P. Bäuerle, *Org. Biomol. Chem.*, **2005**, *3*, 985–995.
